# 73rd Congress of the Italian Society of Pediatrics: Naples, Italy. 30th May - 1st June 2017

**DOI:** 10.1186/s13052-017-0427-z

**Published:** 2017-12-20

## A1 The protection of the child: the role of the Italian ombudsperson for childhood and adolescence

### Filomena Albano (segreteria@garanteinfanzia.org)

#### Autorità Garante Nazionale per l'Infanzia e l'Adolescenza, Rome, Italy

The Italian Ombudsperson for Childhood and Adolescence was established by Law no. 112 of 12 July 2011, with a view to ensuring the full implementation and protection of the rights and interests of persons of minor age, in accordance with the provisions of international conventions, with particular reference to the Convention on the Rights of the Child, signed in New York on 20 November 1989, and implemented in Italy through Law no. 176 of 27 May 1991.

The task of the Italian Ombudsperson for Childhood and Adolescence is to promote and protect the rights of all young people in Italy, irrespective of their nationality, their residence and their links with our Country.

This clarification is all the more necessary in the light of the current historical moment, marked not only by an economic and cultural crisis, but also by a crisis of values and solidarity. Social inequalities still mark today the gap between the condition of children and young people’s well-being and unease in Italy, not only for those born in Italy but also for those living in our Country.

Defeating the existing inequalities between the various areas of the country is a necessity, not only in terms of economic and educational poverty, but also in terms of the right to “live”, to health, to care, to quality of services, to the same rights to access to services that are often translated into a denied right.

Health does not only depend on the absence of biological agents that accidentally cause the disease but is the result of a harmonious, natural and complete development of the individual in every aspect of his/her existence.

In this respect, the Ombudsperson for Childhood and Adolescence carries out preventative and promotional interventions, rather than curative-repairing ones, and in this sense, the interventions planned and realized according to a network approach between services and professions are very important.

The Italian Ombudsperson for Childhood and Adolescence’s commitment is to act responsibly and with the strength of great motivation to ensure that all children and young people have access to equitable care conditions and to an adequate socio-emotional support, irrespective of which part of Italy they were born or they come from, and regardless of their Country of origin.

## A2 Oxygen therapy low and high flow

### Fabio Antonelli, Barbara Borrelli, Fulvio Esposito

#### Pediatric Pneumology AORN Santobono–Pausilipon, Naples, Italy

##### **Correspondence:** Fabio Antonelli (fabantonelli65@gmail.com)

Oxygen therapy remains the first line intervention in acute hypoxemic respiratory failure. The choice of a specific oxygen delivery device is based on the patient's oxygen requirements in terms of flow and desired oxygen concentration, as well as the type of device and its acceptance by the patient [1]. Several medical devices for oxygen therapy, which range from simple nasal cannula to non-rebreathing face masks, have been used in the management of acute hypoxemic respiratory failure. Nasal cannula/prongs is inserted into the patient’s anterior nares. The fractional concentration of inspired O2 (FiO2) varies with the patient’s inspiratory flow. Benefit of this device is here the child can move, sit up, and eat, but there are some limitations also. Maximum flow rates through nasal prongs are 0.5–1 L/min for neonates, 1–2 L/min for infants, 1–4 L/min for older children and there is chance of unpredictable concentration of O2 with excessive mucus drainage.

An air-entrainment mask contains a jet orifice and air entrainment port which is designed to fit over the patient’s nose and mouth. The mask contains Venturi valves which use the principle of jet mixing. This system delivers about 40 L/min of gas through the mask and here breathing pattern will not affect FiO2. The disadvantage is that high flows are noisy and create quite breeze that is cooling. The traditional oxygen therapy devices are constrained by flow limitation, with flows < 15 L/min, by sub-optimal-humidity, by poor tolerance, and by inaccurate and inconsistent F_IO2_. In patients with hypoxemic respiratory failure the patient's inspiratory flow requirements are usually high and very often exceed the oxygen flow delivered by the traditional oxygen devices.

High-flow nasal cannula (HFNC) oxygen therapy represents a new alternative to conventional oxygen therapy [2]. In contrast to the traditional schemes for oxygen therapy, HFNC generates flows up to 60 L/min, yet using a nasal cannula as an interface to the patient. These high flows necessitate the optimal conditioning of the breathing gas in terms of humidification and heating to improve patient comfort. An active form of humidification is generally used during HFNC to condition the high flow gas to optimal heat and humidity (37°C and 44 mg H_2_O/L). Also, an incorporated air-oxygen blender allows the delivery of consistent and accurate oxygen concentrations in the range of 21% to 100% to ensure efficient initial management of hypoxemia in patients with hypoxemic respiratory failure.

References

1. Oxygen therapy for children. World Health Organization. 2016. Available in: http://apps.who.int/iris/bitstream/10665/204584/1/9789241549554_eng.pdf. Accessed in July 10 2017.

2. Dysart K, Miller TL, Wolfson MR, Shaffer TH. Research in high flow therapy: mechanisms of action. Respir Med. 2009; 103:1400-1405.

## A3 Transitional care in respiratory illnesses

### Ermanno Baldo (ermanno@baldo.tn.it)

#### Department of Pediatrics, Rovereto Hospital, Health Care Services-Province of Trento, Rovereto, Italy

The essential elements of "transition" are defined as a planned and intentional process to support teenage patients while they're moving from child to adult health services.

Transition doesn't begin at the same age for everyone and depends on the patient's needs, the current laws and partly on what the patient/client or their family hold to be right [1].

Young adults might indeed slip away from the healthcare system if they get discouraged by the cure and care differences they notice between child and adult health services. This is one of the reasons why the process of moving from child to adult health services is considered to be particularly sensitive [2].

Although health care may be influenced by the training of care workers, teenage patients should be prepared to face transition to adult health services through support, education, orientation and the learning of specific skills allowing a responsible and effective self-management.

A lot of consensus documents [3] suggest the necessity of a transition program for teenage patients suffering from chronic diseases, although this kind of situations are not easy to evaluate and hence the transition process remains hard to manage.

Age, health condition, complex needs, availability of healthcare services (equivalent to those offered to adults) and the creation of a good relationship between teen patients and the team that is going to look after them [4] are key factors proven to have an impact on transition.

Sometimes transition can take place all of a sudden, sometimes teen patients choose to remain longer under child health services or they abandon, more or less voluntarily, the medical control program. When the healthcare system fails to meet the needs of teenage patients and their families during the transitional process, this can lead to patients' health worsening or to movingaway from healthcare, with lasting negative consequences. This has been specifically studied in cases of CF, cystic fibrosis, where children need long-term mechanical ventilation [5] and in cases of housing assistance, where the transitional process should be carried out with non-standard [7] care and attention from patients, doctors and families [6], considering prolonged survival and pathological heterogeinity.

The same complexity and the role played by social, ethnic, familiar and gender differences can also be observed in the studies concerning transitional care of teenage patients suffering from asthma. Here research has found out how the female gender and an inadequate adherence to therapy can have a negative effect leading to a persistent bronchial hyperreactivity [8].

**References**

1. Transition to adult care. Nurs Stand. 2016; 30: 17.

2. Sharma N, O'Hare K, Antonelli RC, Sawicki GS. Transition care: future directions in education, health policy, and outcomes research. Acad Pediatr. 2014; 14:120-7.

3. Acuña Mora M, Moons P, Sparud-Lundin C, Bratt EL, Goossens E. Assessing the level of evidence on transfer and transition in young people with chronic conditions: protocol of a scoping review. Syst Rev. 2016; 5:166.

4. Aldiss S, Cass H, Ellis J, Gibson F. "We Sometimes Hold on to Ours" - Professionals' Views on Factors that both Delay and Facilitate Transition to Adult Care. Front Pediatr. 2016 Nov 24;4:125.

5. Chau SK, Yung AW, Lee SL. Long-Term Management for Ventilator-Assisted Children in Hong Kong: 2 Decades' Experience. Respir Care. 2017; 62:54-64.

6. Anderson DL, Flume PA, Hardy KK, Gray S. Transition programs in cystic fibrosis centers: perceptions of patients**.** Pediatr Pulmonol.2002;33:327-31.

7. Flume PA, Anderson DL, Hardy KK, Gray S.Transition programs in cystic fibrosis centers: perceptions of pediatric and adult program directors. Pediatr Pulmonol. 2001;31:443-50.

8. Yawn BP, Rank MA, Cabana MD, Wollan PC, Juhn YJ. Adherence to Asthma Guidelines in Children, Tweens, and Adults in Primary Care Settings: A Practice-Based Network Assessment. Mayo Clin Proc. 2016; 91:411-21.

## A4 Group B vitamins complex in pediatric age

### Giuseppe Banderali, Federica Betti, Alice Re Dionigi, Valentina Sottili, Elvira Verduci

#### Department of Clinical Paediatric, San Paolo Hospital, University of Milan, Milan, Italy

##### **Correspondence:** Valentina Sottili (valentina.sottili@unimi.it)

Group B vitamin complex includes eight water soluble substances considered essential nutrients playing a key role in cellular, metabolic and enzymatic functioning. Their deficiency has a pleiotropic manifestation, ranging from minor effects to very severe consequences like cardiopathy, encephalopathy, anemia, convulsions, cytopenia, etc. [1, 2, 3].

The reference values of B vitamins dose requirement are defined by LARNs, differing from pregnancy to breastfeeding and among different age group. According to scientific evidence, if a varied diet is followed, B vitamin supplementation is not indicated [4]. In the first six months of life the intake of B vitamins depends on the assumed milk. In case of breastfeeding the maternal dietary intake is important for pyridoxine, niacin, pantothenic acid and cobalamin [5, 6, 7, 8], that present a dynamic milk concentration after the first weeks of lactation (increase in pyridoxine and decrease in cobalamine amount). On the contrary milk thiamine level is not influenced by maternal diet [9], while folates one just only a little for the presence in mammary glands of protein uptaking them from circulation [10]. The use and choice of hypoallergenic/hydrolisate formulas, expecially in not evidenced based diet exclusion, should be as accurate as possible, for the risk of biotin deficiency in case of inadequate product [11]. After six months of age or after weaning, food starts being the main source of B vitamin intake. A balanced diet is generally enough not to have B vitaminic deficiency, but there are some factors to consider besides age and ethnicity to identify risk categories with increased needed of B vitamin supplementation. Genetic polymorphisms of vitamin metabolism like mutation of the gene SCL19A3, coding tiamine type 2 transportant [12], chronic pathologies such as obesity, intestinal malabsorption and tumors [13], drugs (antituberculosis, pump inhibitors, anticonvulsants and antimalarials) and special not well supplemented dietary regimes as vegan diet [14, 15], may increase the risk of group complex B deficiency. Recurrent infections, frequent use of antibiotics, physical activity (that increases B2 requirements up to 20%) and methods of preparation/storage of food (thermolability of vitamin B2, B3, partially B12 and photosensitivity of B2, B6) also influence nutritional assessment. Finally, it is useful to remember the importance of microbiota, as commensal human bacteria are able to produce cobalamin, folates, thiamine, playing a critical role in energy metabolism of the host [16].

**References**

1. Sechi GP, Serra A. Wernicke’s encephalopathy: new clinical settings and recent advances in diagnosis and management. Lancet Neurol. 2007; 6:44255.

2. Smedts HP, Rakhshandehroo M, Verkleij Hagoort AC, et al. Maternal intake of fat, riboflavin and nicotinamide and the risk of having offspring with congenital heart defects. Eur J Nutr .2008; 47:35765.

3. Kliegman R, Stanton B, St. Geme J, Schor N. Nelson Textbook of Pediatrics, 2 Volume Set, 20th Edition. Elsevier, 2015.

4. LARN–Livelli di Assunzione di Riferimento di Nutrienti ed energia per la popolazione italiana. IV Revisione 2014.

5. EFSA Panel on Dietetic Products, Nutrition and Allergies (NDA). Scientific Opinion on Dietary Reference Values for niacin. EFSA Journal. 2014; 12:3759.

6. EFSA Panel on Dietetic Products, Nutrition and Allergies (NDA). Scientific Opinion on Dietary Reference Values for cobalamin (vitamin B12). EFSA Journal. 2015; 13:4150.

7. EFSA Panel on Dietetic Products, Nutrition and Allergies (NDA). Dietary Reference Values for vitamin B6. EFSA Journal. 2016; 14:4485.

8. EFSA Panel on Dietetic Products, Nutrition and Allergies (NDA). Scientific Opinion on Dietary Reference Values for pantothenic acid. EFSA Journal. 2014; 12:3581.

9. EFSA Panel on Dietetic Products, Nutrition and Allergies (NDA). Dietary Reference Values for thiamin. EFSA Journal. 2016; 14:4653.

10. EFSA Panel on Dietetic Products, Nutrition and Allergies (NDA). Scientific Opinion on Dietary Reference Values for folate. EFSA Journal. 2014;12:3893.

11. Hayashi H, Tokuriki S, Okuno T, Shigematsu Y, Yasushi A, Matsuyama G et al. Biotin and carnitine deficiency due to hypoallergenic formula nutrition in infants with milk allergy. Pediatr Int. 2014; 56:286-8.

12. Kono S, Miyajima H, Yoshida K, et al. Mutations in a thiamine transporter gene and Wernicke’s like encephalopathy. N Engl J Med. 2009; 360:17924.

13. PinhasHamiel O, DoronPanush N, Reichman B, et al. Obese children and adolescents: a risk group for low vitamin B12 concentration. Arch Pediatr Adolesc Med. 2006; 160:9336.

14. Rodà D, Rozas L, Fortuny C, et al. Impact of the Increased Recommended Dosage of Isoniazid on Pyridoxine Levels in Children and Adolescents. Pediatr Infect Dis J. 2016; 35:5869.

15. Sieri S, Agnoli C, Baroni L, Bertini I et al. Diete vegetariane: documento SINU 2015.

16. LeBlanc JG, Chain F, Martín R, Courau S, Langella P. Beneficial effects on host energy metabolism of short-chain fatty acids and vitamins produced by commensal and probiotic bacteria. Microb Cell Fact. 2017; 16:79.

## A5 Therapy of pediatric multiple sclerosis

### Angelo Ghezzi, Damiano Baroncini

#### Multiple Sclerosis Study Center of Gallarate, Gallarate, Italy

##### **Correspondence:** Angelo Ghezzi (angelo.ghezzi@asst-valleolona.it)

Pediatric multiple sclerosis (ped-MS) represents about 3-10% of all cases of MS. Compared with the adult form, ped-MS is more likely to have cerebellum and brainstem involvement, a polysymptomatic presentation and a high relapse rate, with >90% of cases being relapsing-remitting [1]. In some patients, the evolution is aggressive since onset. Disease progression in ped-MS is slower than in adult, but disability is greater at a younger age due to the earlier onset.

To date, there have been no randomised controlled clinical trials of drugs in ped-MS. However, results from observational studies have shown that immunomodulant drugs (beta-interferon and glatiramer acetate) are well tolerated and significantly reduce the relapse rate and disease progression in this population [2-3]. European guidance recommends early initiation of immunomodulant therapy for relapsing ped-MS [2]. The International Pediatric MS Study Group recommends the use of first-line therapies (beta-interferon or glatiramer acetate) for all patients with ped-MS [3]. However, about 30% of patients will continue to progress and relapse and may require second-line therapy.

Among second-line treatments, natalizumab has been shown to be highly effective in ped-MS in many observational studies [4], representing a useful option for active MS patients. The limit of natalizumab treatment is the risk of viral encephalitis due to JC virus (JCV) infection (progressive multifocal leucoencephalopathy -PML-). The risk of PML can now be stratified according to presence and titre of anti-JCV antibodies, previous use of immunosuppressors and number of natalizumab infusions [5].

Immunosuppressors have been evaluated in small cohorts of ped-MS patients. Cyclophosphamide significantly reduced disease activity in a retrospective study of 17 patients with ped-MS, but was associated with high incidence of adverse events [6]. Mitoxantrone has shown a beneficial effect in 19 patients with highly active ped-MS, but the safety profile (risk of leukaemia and cardiomyopathy) is discouraging [7].

International randomised controlled studies are ongoing to better define safety and efficacy of newly available drugs for MS treatment (e.g. fingolimod, teriflunomide, alemtuzumab) in the pediatric population.

**References**

1. Waldman A, Ghezzi A, Bar-Or A, Mikaeloff Y, Tardieu M, Banwell B. Multiple sclerosis in children: an update on clinical diagnosis, therapeutic strategies, and research. Lancet Neurol. 2014; 13:936-48.

2. Ghezzi A, Banwell B, Boyko A, Amato MP, Anlar B, Blinkenberg M et al. The management of multiple sclerosis in children: a European view. Mult Scler. 2010; 16:1258-1267.

3. Chitnis T, Tenembaum S, Banwell B, Krupp L, Pohl D, Rostasy K et al. Consensus statement: evaluation of new and existing therapeutics for pediatric multiple sclerosis. Mult Scler. 2012; 18:116-127.

4. Ghezzi A, Moiola L, Pozzilli C, Brescia-Morra V, Gallo P, Grimaldi LM et al. Natalizumab in the pediatric MS population: results of the Italian registry. BMC Neurol. 2015; 15:174.

5. Plavina T, Subramanyam M, Bloomgren G, Richman S, Pace A, Lee S et al. Anti-JC virus antibody levels in serum or plasma further define risk of natalizumab-associated progressive multifocal leukoencephalopathy. Ann Neurol. 2014; 76:802-12.

6. Makhani N, Gorman MP, Branson HM, Makhani N, Gorman MP, Branson HM. Cyclophosphamide therapy in pediatric multiple sclerosis. Neurology. 2009; 72:2076-82.

7. Etemadifar M, Afzali P, Abtahi SH, Ramagopalan SV, Nourian SM, Murray RT et al. Safety and efficacy of mitoxantrone in pediatric patients with aggressive multiple sclerosis. Eur J Paediatr Neurol. 2014; 18:119-25.

## A6 To be born today in Italy: the role of the Ministry of Health

### Serena Battilomo (s.battilomo@sanita.it)

#### General Directorate for Prevention, Ministry of Health, Rome, Italy

The health and wellbeing of children and adolescents are a priority for national and international health policies, as stated in the Minsk Declaration of October 2015. The Italian Ministry of Health devotes a lot of attention, not only to the period of birth, but also to the actions in the first 1000 days of life: from the conception to two years of age. Believing that timely preventive interventions made in this period lead to positive short, medium and long-term health outcomes. In this perspective and having as primary goal the overall health protection, as confirmed also by the provisions about the Essential Care Level (DPCM of January 2017), the Ministry of Health is investing:

- in pre-conception health and support for the couple before and after birth (art. 24),
- in protection of pregnancy and maternity (art.59)
- in early diagnosis of congenital illness, hereditary metabolic diseases, deafness and hypo-vision (art. 38),
- in vaccination (art.4),
- in ensuring the equity of care for foreign children present on national territory too (art. 62).

Special attention has been dedicated to the policies regarding healthcare for mother and child through the December 2010 Agreement between the Central Government and Regional Governments (titled “Guidelines for the promotion and improvement of the quality, safety and appropriateness of care interventions during the birth pathway and for the reduction of caesarean sections”). The agreement established an organizational model of assistance, which accompanies women or couples and their new-born before, during and after the birth under the continued monitoring of the Ministry of Health by the National Birth Pathway Committee.

Another agreement titled “Guidelines for the promotion and improvement of the quality, safety and appropriateness of care interventions in paediatric and adolescent field” is ongoing. The empowerment of parents is another field in which to invest for health through preventive actions useful to create the “health packages” that will always accompany the new-born. For this purpose, the Ministry of Health has supported preventive interventions for couples, women and new-borns (www.pensiamociprima.net), education and training for healthcare professionals and future parents (www.genitoripiu.it) and communication campaigns on breastfeeding. In addition to promoting programs and actions, the Ministry of Health monitors their implementation with annual reports (birth pathway monitoring, essential levels of care monitoring) and their efficacy through specific surveillance systems on maternal mortality, perinatal mortality, “first years” - surveillance for children aged 0-2 years, Okkio for children school aged and HBSC for adolescents.

## A7 Telemonitoring home program in patients with cystic fibrosis: our 15 years’ experience

### S. Bella, F. Murgia

#### Department of Specialist Pediatrics, Integrated Home Care for Chronic Diseases, Bambino Gesù Pediatric Hospital–IRCCS, Piazza S. Onofrio 4, 00165, Rome, Italy

##### **Correspondence:** S. Bella (telemedicina@opbg.net)

The natural history of Cystic Fibrosis (CF) is characterized by recurrent episodes of respiratory infection that causes a progressive pulmonary damage, with decay of long-term lung function leading to death. In CF patients, spirometry shows a 2% reduction every year of Forced Expiratory Volume in the first second (FEV1) over time. In case of pulmonary infection, an early antibiotic treatment helps to prevent more serious complications, limiting consequently the long-term pulmonary damage. Since 2001, in CF Centre of the Pediatric Hospital Bambino Gesù in Rome, we tested the possibility of using Telemedicine (TM) to facilitate the home follow-up of patients with CF. FEV1 was monitored at home, with a view to early recognition of pulmonary relapses. The study has involved 50 patients affected by CF, followed at our Unit with THC in addition to the usual therapeutic protocol, for a total period of 15 years. The balance of enrolment showed a drop-out of 36%, the main cause was poor adherence (68%). We used various and different equipment in this period, also following the progress of technology in this field. The trend of both quantitative and qualitative parameters of our work has been positive for all the equipment. The data are encouraging about the possible role of TM in the homecare organization of chronic diseases. In the current state, however, reliable data on the long-term direct effectiveness of the use of Telehomecare in CF are lacking. The major benefits of using telemedicine would seem to be indirect effects as a stronger and better doctor-patient relationship and an increase in the quality of life for the patient, which could ultimately contribute to an increase in life expectancy.

## A8 Human milk fortifiers for preterm infants

### Enrico Bertino^1^, Guido Eugenio Moro^4^, Paola Tonetto^1^, Chiara Peila^1^, Elena Spada^1^, Giulia Ansaldi^1^, Sonia Deantoni^1^, Laura Cavallarin^2^, Marzia Giribaldi^2,3^, Alessandra Coscia^1^

#### ^1^Neonatology and Neonatal Intensive Care Unit, University Hospital, Città della Salute e della Scienza, Turin, Italy; ^2^Institute of Sciences of Food Production, CNR, Grugliasco (TO), Italy; ^3^Agricultural Engineering and Food Technology Research Center, Turin Laboratory, CREA, Turin, Italy; ^4^Presidente AIBLUD, Milano, Italy

##### **Correspondence:** Enrico Bertino (enrico.bertino@unito.it)

Early postnatal period corresponds to a critical window for development, during which undernutrition can have permanent effects on the development of the central nervous system. Given its unique nutritional and functional advantages, human milk (HM) should be considered as the first choice for the nutrition of all infants, including preterm newborns. Since its protein, mineral and energy contents are not suitable to meet the high needs of very-low-birth-weight (VLBW) infants, HM should be fortified for these components [1;4].

Fortification of HM is an important nutritional intervention in order to provide appropriate nutritional intake and appropriate growth [2].

The standard fortification strategy has yielded inadequate protein intakes, resulting in slower growth as compared to preterm formulas. The main factor responsible for limited success is based on routine assumptions about the composition of HM: the common practice is to add a fix amount of fortifier, assuming that HM has an average protein content and the infant has an average protein requirement. But the protein concentration of preterm milk is variable and decreases with the duration of lactation.

Improvement of outcomes depends on new fortification strategies, considering the large variability of HM composition. Individualized fortification, either targeted or adjustable, has been shown to be effective and practical in attaining adequate protein intakes and growth [3].

The optimal qualitative composition of fortifiers is also a critical issue. Most commercially available multi-nutrient fortifiers and protein concentrates are derived from bovine milk (BM), which has a protein composition very different from that of HM. The use of BM proteins has been recently questioned for possible association with intestinal inflammation in VLBW infants.

Recently, HM-based fortifiers were shown to be associated with lower necrotizing enterocolitis rates and lower mortality in extremely premature infants, compared to BM-based products. However, available data are limited, and its use it’s still debated [5].

Other milk sources are currently under evaluation: donkey and human milk diet integration was shown to be associated with a decrease of inflammatory status and with the improvement of lipid and glucose metabolism in a murine model, when compared to a diet integration with BM. The functional similarity of human and donkey milk is probably due to their closeness in quantitative and qualitative protein, glucidic and lipid fractions composition, that differ to that of BM.

Currently, a randomized, controlled, single-blind clinical trial, coordinated by the Neonatal Unit of the University of Turin is being carried out to evaluate the adequacy of new fortifiers derived from donkey milk for the nutrition of preterm infants.

References

1. Lucas A, Morley R, Isaacs E. Nutrition and mental development. Nutr Rev. 2001; 59:S24-32.

2. Ziegler EE. Breast-milk fortification. Acta Paediatr. 2001; 90:720-3.

3. Arslanoglu S, Moro GE, Ziegler EE. Preterm infants fed fortified human milk receive less protein than they need. J Perinatol. 2009; 29:489-92.

4. Moro GE, Arslanoglu S, Bertino E, Corvaglia L, Montirosso R, Picaud JC, et al. Human milk in feeding premature infants: consensus statement. J Pediatr Gastroenterol Nutr. 2015; 61:S16-19.

5. Mimouni FB, Nathan N, Ziegler EE, Lubetzky R, Mandel D. The Use of Multinutrient Human Milk Fortifiers in Preterm Infants, A systematic Rewies of Unasswered Questions. Clin Perinatol. 2017; 44:173-178.

## A9 Pediatric nurse: training programme and rules

### Gianni Bona^1^, Simonetta Bellone^1^, Marisa Bonino^2^, Maria Donis^1^

#### ^1^Division of Pediatrics, Department of Health Sciences, Università del Piemonte Orientale, Novara, 28100, Italy; ^2^ Course of Paediatric Nursing Degree, Department of Health Sciences, Università del Piemonte Orientale, Novara, 28100, Italy

##### **Correspondence:** Gianni Bona (gianni.bona@maggioreosp.novara.it)

Recently in Italy we have attended to a very controversial debate about the need to change the course of Pediatric Nursing degree, transforming it in a postgraduate master of the General Nursing degree. This argument is yet well known in other european countries in which the importance of keeping the distinction of the two courses has been recognized.

Until 1997, the general nurse in Italy cared for patients of all ages, and although there was a 1 year post qualifying course for certification in children’s nursing it wasn’t required by low.

In 1997, a government decree defined the profile of children’s nurse and in 2001, specific education for children’s nursing (3 years at university level) was started. Despite that currently there is still a large overlap of the two positions and, while a Children Nurse cannot provide care to an adult, a General Nurse can legally be assigned to a Neonatal Intensive Care Unit without ever being trained in this assignment.

Currently over 10 million citizen in Italy are in pediatric age, they represent the 18% of the entire population, thus they constitute a priority in the landscape of Italian health planning.

The Paediatric Nursing Associations of Europe specifies that a course of study should prepare the pediatric nurse to be able to: deliver rights-based, holistic child and family-centred care, promotes physical and mental health and well-being, provide nursing care of infant, child and adolescent with acute/chronic/life threatening/limiting physical and mental conditions, disability or impairment.

The improvements in medical care has carry to increased survival and best prognosis in pediatrics departments, with a strong need of highly qualifying staff for the assistance of ill children.

Italian general hospitals with pediatric departments almost always prefer to hire General Nurses rather than Children Nurses, because the former, albeit insufficiently prepared, make staff management easier for nursing directors. Italian Children Nurses are penalized by this situation, in addition, they must defend themselves from a part of the Italian nursing leadership, which periodically tries to eliminate the pediatric nursing profession.

The Paediatric Nursing Associations of Europe have recognized the need for maintence of the pediatric nurse body and asked to the Parliament and Commission that children and their families can always been assisted by staff whose skills are guaranteed by a specific core curriculum.

## A10 The adolescent with delay of puberty

### Mauro Bozzola (mauro.bozzola@unipv.it)

#### Department of Internal medicine, Pediatric and Adolescentology Unit, University of Pavia, Fondazione IRCCS Policlinico San Matteo, Pavia, Italy

Delayed puberty (DP) is defined as the absence of breast development by 13 years in girls and the lack of testicular enlargement by 14 years in boys. Constitutional delay of growth and puberty (CDGP) is the most common cause of DP, mainly in boys, and is characterized by short stature and delayed skeletal maturation. A family history including pubertal onset of parents and a careful physical examination comprising height, weight, growth velocity and sexual maturity by Tanner staging may provide clues about the cause of DP. It is not a disease, but generally represents a common normal variant in pubertal timing, with good prognosis about final height and future reproductive capacity. In adolescents with CDGP a growth delay (slowing down) occurs until just before the start of puberty, then the growth rate rapidly increases (pubertal growth spurt). Bone age, that is a useful measurement allowing assessment of remaining growth potential, is delayed. CDGP is a diagnosis of exclusion, and alternative causes of DP need to be considered. Functional hypogonadotropic hypogonadism may be observed in patients with transient delay in hypothalamic-pituitary-gonadal axis maturation due to associated conditions such as celiac disease, inflammatory bowel diseases, kidney insufficiency and anorexia nervosa. Permanent hypogonadotropic hypogonadism characterized by low testosterone or estradiol values and blunted FSH and LH levels can be caused by central nervous system abnormalities and can be isolated such as in Kallmann syndrome, or associated with other hormone deficiencies such as multiple pituitary hormone deficiency. Magnetic resonance imaging is necessary to exclude morphological abnormalities and neoplasia. Neither baseline nor stimulated gonadotrophins by GnRH injection can easily differentiate CDGP from permanent hypogonadotropic hypogonadism. In patients with low testosterone in males and estradiol values in females associated with high FSH and LH levels (hypergonadotropic hypogonadism) karyotype can reveal a chromosomal abnormality such as Turner syndrome in girls and Klinefelter syndrome in boys. If the adolescent with CDGP is experiencing psychological difficulties (particularly bullying) a treatment should be offered. In boys more than 14 years of age without pubertal signs can be treated with low dose testosterone in tablets or i.m. injection over 6-9 month period to gently induce puberty. In females treatment is not common, but in girls more than 13 years of age very small amount of estradiol (approximately one eight of adult dose) may be started as oral tablets or transdermal patches for up to 12 months to induce breast development. Once puberty has started, treatment is stopped.

## A11 Gastroesophageal reflux and cow’s milk allergy: treatment and feeding

### Sandra Brusa, Barbara Battistini

#### Department of Medicine and Oncology, Pediatrics and Neonatology Unit, S.Maria della Scaletta Hospital, Imola (BO), Italy

##### **Correspondence:** Sandra Brusa (s.brusa@ausl.imola.bo.it)

Gastroesophageal reflux disease (GERD) and cow’s milk allergy (CMA) are common conditions in pediatric patients, especially infants. They are difficult to diagnose, as there is a lack of a validated diagnostic test and they may be confused with many other conditions. The simultaneous treatment of both conditions often causes exaggerations, resulting in unnecessary pharmacological treatment or elimination diet. The purpose of this presentation is to establish the current scientific evidence for the diagnosis and treatment of GERD secondary to CMA.

Several studies support the hypothesis that there is a causal relationship between GERD and CMA, suggesting that there is a subgroup of infants in whom GERD is attributable to CMA. In 2009 the consensus of the NASPGHAN/ESPGHAN on GERD advises a therapeutic trial of two to four weeks with an extensively hydrolyzed (eHF) or aminoacid formula (AAF), and for infants who are breastfed with a maternal strict cow’s milk protein elimination diet [1]. In 2015, the NICE guideline on GERD evaluates the effectiveness and cost effectiveness of a trial of hydrolyzed formula in formula-fed infants with frequent regurgitation associated with marked distress and concludes that hydrolyzed formula is more expensive than cow’s milk formula and there is no evidence on the clinical or cost effectiveness of this approach [2]. On the other hand, the most practical test in routine practice when there is the suspicion of GERD secondary to CMA is a trial of cow’s milk protein elimination diet for 2-4 weeks [3]. A good practice could be to do this test only in the subgroups of patients, in general infants < 6 months with GERD, in whom there is a high index of suspicion for CMA: infants with other atopic conditions, infants with a strong family history of atopy and finally infants who have not responded to the initial management of GERD with conservative treatment and alginates. A randomised controlled trial is required to explore this question. If there is a clear response to the elimination diet and the oral food challenge confirms the diagnosis of CMA, the infant should be maintained on an elimination diet using a therapeutic formula for at least 6 months or until 9 to 12 months of age. Infants should grow and thrive normally when treated with either eHF or AAF formula with proven efficacy [4].

The relationship between GERD and CMA remains unclear and there are exaggerations in the diagnosis and treatment, which need to be corrected [5].

References

1. Vandenplas Y, Rudolph CD, Di Lorenzo C, Hassall E, Liptak G, Mazur L, et al. Pediatric gastroesophageal reflux clinical practice guidelines: joint recommendations of the North American Society for Pediatric Gastroenterology, Hepatology, and Nutrition (NASPGHAN) and the European Society for Pediatric Gastroenterology, Hepatology, and Nutrition (ESPGHAN). J Pediatr Gastroenterol Nutr. 2009; 49:498-547.

2. National Institute of Health and Care Excellence (NICE). Clinical knowledge summaries on gastroesophageal reflux disease in children. Available on www.nice.org.uk/guidance/NG1.

3. Berni Canani R, Di Costanzo M, Troncone R. The optimal diagnostic workup for children with suspected food allergy. Nutrition. 2011; 27:983-987.

4. Koletzko S, Niggemann B, Arato A, Dias JA, Heuschkel R, Husby S, et al. Diagnostic approach and management of cow’s-milk protein allergy in infants and children: ESPGHAN GI Committee Practical Guidelines. J Pediatr Gastroenterol Nutr. 2012; 55:221-229.

5. Ferreira CT, de Carvalho E, Sdepanian VL, de Morais MB, Vieira MC, Silva LR. Gastroesophageal reflux disease: exaggerations, evidence and clinical practice. J Pediatr (Rio J). 2014; 90:105-118.

## A12 Vaccines: science and lies

### Roberto Burioni, Nicola Clementi, Nicasio Mancini

#### Laboratorio di Microbiologia e Virologia Medica, Università “Vita-Salute” San Raffaele, Milano, Italia

##### **Correspondence:** Roberto Burioni (burioni.roberto@hsr.it)

Incorrect and tendentious campaigns on the potential risks associated to vaccinations still contribute to the spread of false theories associating vaccinations with a plethora of diseases. Obviously, none of these grotesque antivax theories is ground on objective data generated and analyzed following the scientific method. The aversion to vaccination is as old as vaccines themselves, but it is now exponentially amplified by the capillary worldwide diffusion of the internet and the social networks. The approach of science tells us that vaccines are safe, reliable and shield children, and the whole of society, from very dangerous diseases. Nonetheless, if you browse the Internet you find an exceptional number of “doctors” and members of the chattering classes who say exactly the opposite and who want to fool the whole community. In other words, anyone, even without scientific expertise, can spread false theories through the web, instigating people to support conspiracy theories such those against the vaccine practices. Unfortunately, this put the dishonest campaigns of self-proclaimed “free-thinkers” at the same level of the scientific evidences on vaccines widely shared and accepted by the scientific community. The Internet is a place where facts and opinions mingle and blend, a place where all voices – respectable or not – are on the same level; a place where there are no filters and where you can find both the firefighter and the pyromaniac talking about fire prevention! Therefore, the new challenges for the medical community are represented by the need of sensitizing everybody once more to the need of vaccines as pivotal weapon against microbial infections. This main goal cannot be achieved without a mandatory regular training and correct information of medical doctors on scientific evidences related to vaccines currently in use. In fact, only the formation of true professionals on vaccination practices and risks will allow containing the ever increasing number of false experts on the web and their antisocial behaviors putting at risk our community.

## A13 Expanded neonatal screening: state of art and future prospective

### Alberto Burlina (alberto.burlina@unipd.it)

#### Division of Inherited Metabolic Diseases, Reference Centre Expanded Newborn Screening, Department of Woman's and Child's Health, University Hospital, Padova, Italy

In the 1980s, MS-MS methods were introduced for the detection of acylcarnitines in plasma and urine. This technique has been extended to newborn screening (NBS) because of the development of automated methods of sample preparation and injection into the instrument. This allows for multiple biochemical parameters to be tested in a single dried blood spot (10).

The advantages of expanded NBS are that it allows a large number of presymptomatic diagnosis presenting severe disability for some and offers a genetic counseling for the couple of parents; it also decreases the risk of delayed diagnosis, quite always associated to disabilities, of relatively treatable diseases. Recently (October 2016) a law has been approved by the Italian Government. This includes a fixed panel of 36 inborn errors of metabolism and the time course of the entire process. Notably, the law defined withdrawal, testing and processing time that the screening center has to operate. Briefly, expanded neonatal screening in Italy includes the following disorders:

Amino acid disorders and urea cycle defects (argininemia, Argininosuccinic aciduria Citrullinemia I and II, Homocystinuria and Cobalamin deficiency, MSUD, phenylketonuria Biopterin cofactor biosynthesis defect, Biopterin cofactor regeneration defect Tyrosinemia I and II; Organic acidemias Beta-ketothiolase deficiency Multiple carboxylase deficiency HMG-CoA lyase deficiency 3-methylcrotonyl-CoA carboxylase deficiency Glutaric acidemia Type 1 Isovaleric acidemia Malonic acidemia Methylmalonic acidemias Propionic Acidemia; Fatty acid oxidation defects Carnitine uptake deficiency, Carnitine Palmitoyltransferase type I and II deficiency, MCAD, VLCAD, LCHAD, glutaric aciduria type II).

All these disorders are diagnosed by tandem mass spectrometry. Moreover, galactosemia and biotinidase have been included in the panel but they need different analytical techniques.

Future directions of expanded neonatal screening will include lysosomal disorders (LSD) and peroxisomal defects. Recently, LSDs have become strong candidates for inclusion in future mandatory screening panels due to new effective therapeutic options available and to the development of new analytical methods to test enzyme activity on DBS specimens. These conditions include Pompe disease, Niemann-Pick type A/B disease, Fabry disease, Krabbe disease, Mucopolysaccharidoses type I, and Gaucher disease. An additional argument for inclusion of LSDs in NBS programs is the relative prevalence of these conditions. Neonatal screening for LSDs needs some ethical considerations including pre-symptomatic individuals with positive screening results, about the best way to inform parents of the potential outcomes of the affected individual and risks for future pregnancies.

## A14 The Editorial Process: behind the scenes

### Carlo Caffarelli, Dora Di Mauro, Carla Mastrorilli

#### Clinica Pediatrica, Dipartimento di Medicina e Chirurgia, Università degli Studi di Parma, Parma, Italy

##### **Correspondence:** Carlo Caffarelli (carlo.caffarelli@unipr.it)

The responsibilities of the Editor include ensuring the smooth running of the editorial process, which is facilitated by the Publisher, overseeing the article submission process and, for accepted articles, assisting Authors in preparing their final versions so they are ready for copyediting. Editors are responsible for identifying important ‘hot topics’, sourcing high quality manuscripts, handling day-to-day paperwork, and organizing the flow of manuscripts (i.e. from author to referees and back and finally to the publisher). In the publisher service, the Journal Development Editor constantly communicates with the Editors for routine work and specific issues. Editors’ work must be arranged through a letter of agreement with the publisher on management modalities. After submission, Editor reads the manuscript and evaluates the submission and makes an initial decision whether the manuscript is likely to be a strong candidate for peer review or not. Submission department verifies that the manuscript does not have plagiarisms or has not been previously published elsewhere. The Editor may send Authors a decision letter, with reasons for rejection. This often happens when paper does not fit the scopes of the journal or does not provide significant conceptual advances. Conversely, when the manuscript is suitable, Editor selects and invites appropriate reviewers. Peer review process is important to provide second opinions on papers and give Editors means to take a decision. After reviewers’ evaluation, the Editor and, if necessary, the Editorial team assess peer reviews and make a decision on the manuscript. Three outcomes are possible: acceptance, further revisions, or rejections. Editor communicates the judgment in a decision letter. After this step, when the manuscript has been accepted, the Production Team manages the process of type-setting, proof-reading and incorporating Authors’ correction. The proofed article is ready for online publication.

**References**

1. Wager E, Parkin EC, Tamber PS. Are reviewers suggested by authors as good as those chosen by editors? Results of a rater-blinded, retrospective study. BMC Medicine. 2006; 4:13.

2. Duff JM, Leather H, Walden EO, LaPlant KD, George TJ. Adequacy of published oncology randomized controlled trials to provide therapeutic details needed for clinical application. J Natl Cancer Inst. 2010; 102:702–705.

## A15 Parenting and adolescence with a rare disease

### Giulietta Angelelli Cafiero (presidente@aidel22.it)

#### AIdel22 Onlus, Rome, Italy

Through adolescence every teenager continuously swings from one to another of four worlds: the family, within which the teenager always finds support and rescue; the adults, by whom the teenager continually strives to be accepted and recognized; his/her psychological reality, the individual world in which the adolescent takes refuge when confronted with conflicting emotions; and the peer group, in which the young man/woman finds a group support [1]. Unfortunately, the oscillations back and forth between these four worlds are very limited for young people with disabilities, because they tend never to leave their family; consequently, other realities - the other "worlds" - can become unattainable [2,3,4]. On the other hand, parents tend not to give a teenage child with a rare disability the opportunity to assert his will on the outside world and make sense of the reality surrounding him/her, as they are always ready to step in and make choices for the child at times in his/her life when making these choices would be fundamental for building an independent self [5]. It is important for a parent to know the degree of acceptance that the teenager has achieved with regard to his condition and how the teenager recognizes and manages the emotional aspects of life, because a teenager with rare illness always has a past characterized by hospitalizations, learning, nutritional or language disorders or psychomotor development issues [6], which surely led him/her to devise his/her own ways of dealing with each situation. In addition to having to deal with a specific clinical field that will test the teenager’s ability to accept his/her condition as a rare disease patient, in the school context the adolescent will be faced with the evidence of his/her diversity, revealed by the presence of a special education teacher at his/her side. When involved in social, sports and recreational activities the teenager will have to come to terms with the peculiar physical features determined by his/her rare illness, which, if s/he is not adequately supported, might lead to a high degree of insecurity and which might jeopardize his/her relationships with the world. Parents need to think of their child with a rare illness as a person who is growing and changing, and who will play a role in his/her own future. It is crucial that parents maintain the ability to plan for their child's future, but they must look for and work with other adults - from mental and physical health professionals to family associations - who are able to dream a future for children with disabilities, to invest in their potential and to help them conceive their own life projects [7].

**References**

1. Meltzer D, Harris M. Psicopatologia dell’adolescenza. In: Quaderni di psicoterapia Infantile Vol.1. Roma: Borla; 1993. p. 49-75.

2. Bowlby J. Attachment. In: Attachment and loss. Volume 1. London: Hogarth Press; 1969. (trad. It. Attaccamento e perdita. Volume 1. Torino: Bollati Boringhieri; 1971).

3. Bowlby J. Separation: anxiety and anger. In: Attachment and loss. Volume 2. London: Hogarth Press; 1973. (trad. It. Separazione dalla madre: ansietà e rabbia. Torino: Bollati Boringhieri; 1975).

4. Bowlby J. Loss: Sadness and Depression. In: Attachment and loss. Volume 3. London: Hogarth Press; 1980. (trad. It. La perdita della madre. Torino: Bollati Boringhieri; 1981).

5. Henninger NA. Family perspectives on a successful transition to adulthood for individuals with disabilities. Intellect Dev Disabil. 2014; 52:98-111.

6. Robertson J. Bambini in ospedale. Milano: Feltrinelli; 1973.

7. Montobbio E, Lepri C. Chi sarei se potessi essere. La condizione adulta del disabile mentale. Tirrenia: Edizioni del Cerro; 2000.

## A16 Refusal of treatment in adolescents

### Maria Teresa Carbone, Antonio Correra

#### UO Malattie Metaboliche e Rare, AORN Santobono Pausilipon, Naples, 80126, Italy

##### **Correspondence:** Maria Teresa Carbone (carbonemariateresa247@gmail.com)

Adolescent is peculiar time of the life, characterized by deep change of the body and of the behavior. These problems are more evident in adolescent with chronic or rare disease [1]. Many studies reported that 50%-55% of adolescents with chronic disease drop the treatment [2]. The disease is perceived by an affected adolescent as a treat of the independence and increase the conflicts with the parents. Reasons given in the literature for refusal, noncompliance, and abandonment of treatment by the adolescent include also the patient’s physical discomfort, misunderstanding and uncertainty about the merits of medication [3]. Professional caregivers should acknowledge and respect adolescents' emerging autonomy and values and should understand the reason of adolescent behavior if affected by a chronic illness [4]. The patient centered medicine can be used to avoid the refusal of the treatment [5]. In patient centered medicine the patient take active part of the treatment and he has the privilege to define his needs. The mainstream is the empathy. Sometimes adolescent patients are unable to realize the advantage of the treatment but on the contrary, they understand very well costs, failures and adverse effects [5]. It is very important during periodical medical checkup that the physician understand the main difficulties of the adolescent to maintain the correct treatment. In this way the pediatrician must try to reinforce the positive behavior when present [5]. To gain this goal the only way is a team approach: group of discussion and self-help both for affected children and parents. In this way is possible a psychological share of the problems to produce a less instinctual behavior [2]. The treatment of phenylketonuria is an example. In this metabolic disease, the treatment is only made by special foods, basically for the entire life. The normal foods are forbidden or heavily reduced. The food limitation is badly tolerated by adolescents, since group eating is part of the teenager parties. Even a patient in correct dietary treatment gradually or suddenly stop diet and start eat normal food. High blood levels of phenylalanine can determine irritability, bad feelings, losing of concentration and lack of memory. In pregnancy, the diet with low phenylalanine foods in fundamental to avoid the Phe embriopathy [6]. In this way, a main role is done by regional centers for diagnosis and treatment of chronic illness, where adolescents can find all the supportive skills to induce a correct medical compliance of adolescent patients.

**References**

1. Rapoff MA. Management of adherence and chronic rheumatic disease in children and adolescents. Best Pract Res Clin Rheumatol. 2006; 20:301-14.

2. Shaw JE, Chisholm DJ. Epidemiology and prevention of type 2 diabetes and the metabolic syndrome. Med J Aust. 2003; 179:379-83.

3. Pai AL, Drotar D. Treatment adherence impact: the systematic assessment and quantification of the impact of treatment adherence on pediatric medical and psychological outcomes. J Pediatr Psychol. 2010; 35:383-93.

4. Shemesh E, Annunziato RA, Arnon R, Miloh T, Kerkar N. Adherence to medical recommendations and transition to adult services in pediatric transplant recipients. Curr Opin Organ Transplant. 2010; 15:288-92.

5. Bardes CL. Defining "patient-centered medicine". N Engl J Med. 2012; 366:782-3.

6. Van Spronsen FJ, van Wegberg AM, Ahring K, Bélanger-Quintana A, Blau N, Bosch AM, et al. Key European guidelines for the diagnosis and management of patients with phenylketonuria. Lancet Diabetes Endocrinol. 2017. DOI: 10.1016/S2213-8587(16)30320-5

## A17 The management of hypoglycemia

### Francesca Cardella (cardellafrancesca@gmail.com)

#### ISMEP Pediatric Department, Children Hospital G. Di Cristina, Palermo, 90134, Italy

In patients with diabetes mellitus hypoglycaemia is defined, "any episode where low plasma glucose levels are present, which may be harmful to the patient", thus underlining the high risk of these events at any age but especially during childhood. Despite the advances in insulin therapy and glycemic monitoring, hypoglycaemia remains the leading acute complication of type 1 diabetes [1] and causes anxiety and fear for both patients and their families [2], representing the main limiting factor in achieving good glycemic control [3,4]. The Group of Study on Diabetes of the Italian Society of Endocrinology and Pediatric Diabetes (GdS of ISPED) has therefore decided to draft the "Recommendations on the Prevention and Treatment of Hypoglycemia in Type 1 Diabetes Pediatric Age". To this end, a systematic review of the available scientific evidence has been made.

Given the lack of recommendations or guidelines, it was decided to consult all members of the GdS and, through macro-regional meetings, during which the Metaplan methodology was used, the critical areas were identified. The aim of these recommendations is therefore to achieve uniform behavior among all Italian pediatric diabetologists in prevention, and in the treatment of hypoglycaemia, with particular attention to the psycho-physical well-being of patients and their families. The work with Metaplan, involving more than one hundred diabetologist pediatricians on the three thematic macro-areas, focused on:

1. the factors that most affect hypoglicemia;

2. how to improve the prevention of hypoglycaemia;

3. the barriers to the treatment of hypoglycaemia.

The ISPAD guidelines [5] report the most accepted value for hypoglycemia as 65 mg / dl (3.6 mmol /l), while recognizing the threshold of 70 mg/dl (3.9 mmol/L) as the one to start treatment in diabetic children, just to prevent the risk of a subsequent severe hypoglycaemia. The ADA working group detects threshold value in any subject with diabetes at 70 mg/dl (3.9 mmol/L) [6].

In many diabetes manuals, hypoglycemia is arbitrarily subdivided in mild if the blood glucose is between 60 and 70 mg / dl, moderate if between 50 and 60 mg / dl, and severe if less than 50 mg/dl. In fact, any blood glucose <70 mg/dl should be considered at risk for severe hypoglycaemia as the phenomenon is rapidly variable. Hypoglycemic crisis requiring third-party intervention for the resolution is severe in adults, whereas in the child the onset of seizures or coma is considered necessary for the severity of the hypoglicemia. In my report, I will try to emphasize the various physiopathological, clinical and therapeutic aspects of hypoglycemia management, stressing how today's continuous therapeutic education is required to prevent this phenomenon.

References

1. The Diabetes Control and Complications Trial Research Group, Nathan DM, Genuth S, Lachin J, Cleary P, Crofford O, et al. The effect of intensive treatment of diabetes on the development and progression of long-term complications in insulin-dependent diabetes mellitus. N Engl J Med. 1993; 329:977-986.

2. Pramming S, Thorsteinsson B, Bendtson I, Binder C. Symptomatic hypoglycaemia in 411 type 1 diabetic patients. Diabet Med 1991; 8:217-222.

3. Seaquist ER, Anderson J, Childs B, Cryer P, Dagogo-Jack S, Fish L, et al. Hypoglycemia and diabetes: a report of a workgroup of the American Diabetes Association and the Endocrine Society. Diabetes Care 2013; 36:1384-95.

4. Cryer PE. Hypoglycaemia: the limiting factor in the glycaemic management of Type I and Type II diabetes. Diabetologia. 2002; 45:937-948.

5. Ly TT, Maahs DM, Rewers A, Dunger D, Oduwole A, Jones TW. Assessment and management of hypoglycemia in children and adolescents with diabetes. Pediatr Diabetes. 2014; 15:180-192.

6. ADA, Standards of Medical Care in Diabetes 2017; Diabetes Care. 2017; 40:1-139.

## A18 Acute respiratory failure: upper airway obstruction

### Anna R Cappiello^1^, Violetta Mastrorilli^2^, Paola Passoforte^2^, Giorgia Borrelli^2^, Giuseppina Mongelli^2^, Doriana Amato^3^, Maria F Mastrototaro^3^, Fabio Cardinale^3^

#### ^1^UOC Terapia Intensiva Neonatale e Neonatologia, Ospedale “SS. Annunziata”, Taranto, Italy; ^2^Scuola di Specializzazione in Pediatria, Università degli Studi di Bari “A. Moro”, Bari, Italy; ^3^UOC di Pediatria Generale e Allergo-Pneumologia, Azienda Ospedaliero-Universitaria Consorziale Policlinico-Ospedale Pediatrico Giovanni XXIII, Bari, Italy

##### **Correspondence:** Fabio Cardinale (fabiocardinale@libero.it)

Pediatric respiratory emergency is among the most common reason for hospital admission and result in a significant number of deaths. Acute respiratory failure is a significant mortality cause without an appropriate intervention [1]. Respiratory failure can be due to a variety of causes such as upper and lower airway obstruction, lung disease, impairment of ventilation or failure of NCS ventilation control. Major causes of upper lower airway obstruction (UOA) are croup, foreign body aspiration, epiglottitis, deep neck infection, angioedema and congenital abnormalities [2].

Croup is an inflammatory condition of subglottic airway that typically affects children under 3 year of age. Is typically caused by respiratory viruses with parainfluenza accounting for up to 80% of cases. Children with croup presents with hoarse voice, barking cough and variable degrees of stridor and respiratory distress [3]. Symptoms usually resolves in 48 hours but severe UAO can lead to respiratory failure [4]. The diagnosis should be made clinically and other investigation are only rarely required [5]. A simple treatment algorithm from the TOP guideline [6] based on assessment of severity of respiratory distress can be used to guide management of croup. There is clear evidence that corticosteroids benefit children with croup from mild to severe. Nebulized epinephrine provides rapid short term relief of severe respiratory distress [7].

Inhalation of foreign body (FBI) is a potentially life-threatening emergency [8]. Toys or food particles are responsible for accidental deaths in children especially under 4 years of age [9]. FBI may produce a wide range of clinical symptoms such as coughing, choking and acute dyspnea. Because of undiagnosed and retained FB injury may result in severe early and late complication including asphyxia, pneumonia, atelectasis and bronchiectasis. Timely removal maneuvers are mandatory to prevent complications [10].

Epiglottitis is an acute inflammation of the epiglottis that may lead to the rapid onset of life-threatening airway obstruction. Epiglottitis was most commonly caused by Hib and primarily reported in children aged 2 to 7 years. Introduction of Hib vaccine dramatically changes the epidemiology and today a variety of causative pathogens have been identified [3]. Signs and symptoms of epiglottitis include acute onset of fever, sore throat, dysphagia, drooling, dysphonia and respiratory distress (four Ds) [11]. All patient with epiglottitis should be admitted in ICU for observation and definitive treatment. Securing airway is the initial step in management associated with a broad spectrum antibiotic coverage [3]. Acute UAO from many cause can be life-threatening emergency and require stabilize airway, even if diagnosis is unclear.

**References**

1. Hammer J. Acute Respiratory Failure in Children. Pediatr Respir Rev. 2013;14:64-69.

2. Pfleger A, Eber E. Management of acute severe upper airway obstruction in children. Pediatr Respir Rev. 2013; 14: 70-77.

3. Sobol SE, Zapata S. Epiglottitis and croup. Otolaryngol Clin North Am. 2008; 41: 551-566.

4. Johnson DW. Croup. Systematic review 321. BMJ Clinical Evidence. http://clinicalevidence.bmj.com/x/systematic-review/0321/overview.html. 2014 September. Accessed 21 June 2017.

5. Mazza D, Wilkinson F, Turner T, Harris C; Health for Kids Guideline Development Group. Evidence based guideline for the management of croup. Aust Fam Physician. 2008; 37:14-20.

6. Toward Optimized Practice (TOP) Working Group for Croup Guideline for the Diagnosis and Management of Croup Edmonton (AB): Toward Optimized Practice, 2003 (revised 2008).

7. Bjornson CL, Johnson DW. Croup in children. CMAJ 2013;185:1317-1323.

8. Alfaki M, Alam-Elhuda DM. Airway foreign bodies: A critical review for a common pediatric emergency. World J Emerg Med. 2016; 7:5-12.

9. Sahin A, Meteroglu F, Eren S, Celik Y. Inhalation of foreign bodies in children: experience of 22 years. J Trauma Acute Care Surg. 2013; 74:658-663.

10. Gregori D, Salerni L, Scarinzi C, Morra B, Berchialla P, Snidero S, et al. Foreign bodies in the upper airways causing complications and requiring hospitalization in children aged 0-14 years: results from the ESFBI study.Eur Arch Otorhinolaryngol. 2008; 265:971-978.

11. Losek JD, Dewitz-Zink BA, Melzer-Lange M, Havens PL. Epiglottitis: comparison of signs and symptoms in children less than 2 years old and older. Ann Emerg Med. 1990; 19:55-58.

## A19 Menstrual disorders in adolescents

### Alessandra Cassio, Rita Ortolano, Valentina Assirelli, Giulio Maltoni, Federico Baronio

#### Unit of Pediatrics, Program of Pediatric Endocrinology, Department of Medical & Surgical Sciences, University of Bologna, Bologna, Italy

##### **Correspondence:** Alessandra Cassio (alessandra.cassio@unibo.it)

A trend of reducing the age of menarche in all industrialized countries including Italy was observed from the late 1800s until the second half of 1900. Over the last decades, age of menarche has stabilized around 12-12.5 years [1,2]. A normal menstrual cycle represents the completion of the hypothalamus-hypophysis-ovary (HPO) axis pathway and requires the balance of a complex feedback system. From 1 to 3 years after menarche, about 50% of the menstrual cycles can be anovulatory. Therefore, the phenomenon of menstrual irregularities both as a number of cycles or as a menstrual flow entity is a common phenomenon in the adolescent girl and it is generally characterized by kindness [3]. However, it is important to correctly classify the different menstrual disorders and their main causes, so that pathological forms on the organic basis can be excluded. Currently, according to latest indications of the International Federation of Ginecology and Obstetrics (FIGO), the terminology to classify these disorders has been uniformed and simplified as follows [4]:

1) Absence of menses (primary or secondary amenorrhea)

2) Menses at irregular intervals (unpredictable intervals between episodes of menstrual bleeding)

3) Excessive menstrual bleeding (prolonged > 7 days or > 80 ml/cycle)

4) Intermenstrual bleeding.

It may be difficult to classify the menstrual disorder into a particular pattern for many reasons. The categories may overlap, quantification of menstrual flow can be difficult and the causative conditions may have more than one pattern [5]. The initial triage must include assessment of hemodinamic and hematologic stability in cases of excessive bleeding and the pregnancy status, if needed. History, physical examination and initial evaluation should be focused on identifying the predominant bleeding pattern and the clinical picture associated with the specific causes of abnornmal bleeding. The diagnosis of anovulatory uterine bleeding that is the most common cause of menstrual disorder in the adolescents must be a diagnosis of exclusion.

**Table 1:** **(abstract A19).** Main causes of menstrual disorders in adolescents with their possible clinical manifestations

Causes	Amenorrhea	Irregular bleeding	Excessive bleeding
HP AXIS DISORDERS			
Delayed maturation	X	X	X
Disorders of weight or energy expenditure	X	X	
CNS tumors	X	X	
Hyperprolactinemia	X	X	
Hypogonadism	X		
OVARIAN DISORDERS			
PCOS	X	X	X
Ovarian insufficiency (genetic, iatrogenic, autoimmune)	X	X	
Ovarian tumors		X	X
BLEEDING DISORDERS			X
MISCELLANEOUS CONDITIONS			
Pregnancy	X		X
IUD		X	X
Hypothyroidism	X	X	X
Uterine/vaginal malformations	X		
Androgen insensitivity syndrome	X		

References

1. Parents AS, Franssen D, Fudvoy J et al. Developmental variations in environmental influences including endocrine disruptors on pubertal timing and neuroendocrine control.Revision of human observations and mechanism insight from rodents. Frontiers Neuroendocrinol 2015; 38:12-36.

2. Russo G, Brambilla P, Della Beffa F et al. Early onset of puberty in young girls: an Italian cross-sectional study. J Endocrinol Invest 2012; 35:804-808.

3. Hillard PJA. Menstuation in adolescents: What do we know? And what do we do with the information?J Pediatr Adolesc Gynecol 2014; 27:309-319.

4. Munro MGCritchley HO, Broder MS et al. FIGO classification system for causesof abnormal uterine bleeding in nongravid womenof reproductive age. Int J Gynaecol Obstet 2011; 113:3-13.

5. Mitan LA, Slap GB. Adolescent menstrual disorders.Update. Med Clin North Am 2000; 84:851.

## A20 FED in DSM-5 and the role of the Family Pediatrician in the early diagnosis of Feeding and Eating Disorders in pediatric age

### Serenella Castronuovo^1^, Giampaolo De Luca^2^

#### ^1^Family pediatrician, Gruppo di Studio Nazionale Adolescenza della SIP, Nettuno (RM), 00048, Italy; ^2^Family pediatrician, Gruppo di Studio Nazionale Adolescenza della SIP, Cosenza, 87100, Italy

##### **Correspondence:** Serenella Castronuovo (serenella.castronuovo@gmail.com)

The onset of disorders of feeding behaviour is starting more and more at an early age. DSM-5 [1] changed the nosography of food behaviour disorders here named “Feeding and Eating Disorders” (FED). FED are persistent disorders of the feeding or a behaviour that determine an adulterated food consumption or assimilation and that significantly threaten physical health or social behaviour. They are categorised as follow:

1. Pica
2. Rumination disorder
3. Avoidant/restrictive food intake disorder
4. Anorexia nervosa
5. Bulimia nervosa
6. Binge eating disorder
7. Feeding and nutrition disorder with specification
8. Feeding and nutrition disorder without specification

The first three categories are especially related to eating disorders in childhood. Pica and Rumination disorder are now considered as FED: according to this new criteria the age limit previously used to make the diagnosis has now been cancelled.

It has been noted that the onset of Anorexia nervosa, Bulimia nervosa and Binge eating disorder, already included among eating disorders in DSM IV TR, is taking place earlier and earlier in children.

FED are multifactorial diseases [2, 3], whose frequency is sharply increasing in adolescence [4, 5, 6], characterised by:

- Alteration of the feeding behavior
- Extreme concern for the physical fitness
- Wrong perception of the body appearance

There is a close correlation between these factors and the levels of self-esteem.

Pathogenetics hypothesis of FED: there is often an instability of the symptomatic manifestation related to a trans diagnostics migration, therefore nowadays the conviction is that the various FED are the same entity with different symptomatic manifestations depending on the age and the clinic evolution.

The diagnosis is complex, especially in early adolescence (8-12 years) [3, 7, 8, 9]. The Family Pediatrician usually knows the child since his/her birth and follows his/her growth, both physically and psychologically, and is, therefore, the first who can intercept, through simple diagnostic tests (such as EAT 26), the first signs of these conditions, and from this depends on the subsequent diagnosis, therapy and prognosis [10,11,12,13,14,15,16,17,18].

Subsequently, the role of the family pediatrician is:

- To evaluate the gravity of the disorder through a careful medical examination and eventually laboratory analyses. Blood test is not diagnostic but it is useful to define the gravity of the disease [19,20,21,22] .
- To evaluate the psychological situation, in order to identify the presence of high risk situations, such as severe depression, anxiety, self-mutilations, abuse of drugs or other substances.
- To make differential diagnosis with other organic diseases (endocrine, gastro-intestinal, neuropsychiatric, contagious)

It is important that the family Pediatrician recognize the necessity to send the patient to a second level structure, and in order to do this the diagnostic suspect is essential.

**References**

1. American Psychiatric Association. Diagnostic and Statistical Manual of Mental Disorders. 5. Washington: American Psychiatric Publishing; 2013. Feeding and Eating Disorders; p. 329–354. DSM-5.

2. Brooks SJ, Rask-Andersen M, Benedict C, Schiöth HB. A debate on current eating disorders diagnoses in light of neurobiological findings: is it time for a spectrum model? BMC Psychiatry. 2012. p. 12–76.

3. Dalla Ragione L. I disturbi del comportamento alimentare: un’epidemia della modernità. In: Presidenza del Consiglio dei Ministri, Dipartimento della Gioventù. Il coraggio di guardare: prospettive ed incontri per la prevenzione dei disturbi del comportamento alimentare. Eye 03 Roma. 2012. p. 19–34.

4. Powers PS, Santana CA. Eating disorders: a guide for the primary care physician. Prim Care. 2002; 29:81–98.

5. Favero A, Caregaro L, Tenconi E, Bosello R, Santonastaso P. Time trends in age at onset of anorexia nervosa and bulimia nervosa. J Clin Psychiatry. 2009; 70:1715–21.

6. Dalle Grave R. Eating disorders: progress and challenges. Eur J Int Med. 2001; 22:153–60.

7. Lask B, Bryant-Waugh R, Wright F, Campbell M, Willoughby K, Waller G. Family physician consultation patterns indicate high risk for early-onset anorexia nervosa. Int J Eat Disord. 2005; 38:269–72.

8. Sigel E. Eating disorders. Adolesc Med. 2008; 19:547–72.

9. Centers for Disease Control and Prevention (CDC) Eaton DK, Kann L, Kinchen S, Shanklin Flint KH, Hawkins J, et al. Youth risk behavior surveillance - United States, 2011. MMWR Surveill Summ. 2012; 61:1–162.

10. American Academy of Pediatrics; Committee on Adolescence. Identification and management of eating disorders in children and adolescents. Pediatrics. 2010; 126:1240–53. doi: 10.1542/peds.2010-2821.

11. Nicholls D, Hudson D, Mahomed F. Managing anorexia nervosa. Arch Dis Child. 2011; 96:977–82.

12. Maestro S, Baroncelli GI, Ghione S, Bertelloni S. I disturbi del comportamento alimentare in adolescenza. Prospettive in pediatria. 2013; 170:74–83.

13. Martin H, Ammerman SD. Adolescents with eating disorders. Primary care screening, identification, and early intervention. Nurs Clin North Am. 2002; 37:537–551.

14. Yamamoto C, Uemoto M, Shinfuku N, Maeda K. The usefulness of body image tests in the prevention of eating disorders. J Med Sci. 2007; 53:79–91.

15 .Johnston O, Fornai G, Cabrini S, Kendrick T. Feasibility and acceptability of screening for eating disorders in primary care. Fam Pract. 2007; 24:511–7.

16. Engelsen BK, Hagtvet KA. A generalizability study of the Eating Attitudes Test (EAT-12) in non clinical adolescents. Eating and Weight Disord. 1999; 4:179–186.

17. Anstine D, Grinenko D. Rapid screening for disordered eating in college-aged females in the primary care setting. J Adolesc Health. 2000; 26:338–342.

18. Garner DM, Olmsted MP, Bohr Y, Garfinkel PE. The eating attitudes test: psychometric features and clinical correlates. Psychol Med. 1982; 12:871–8.

19. Winston AP, Barnard D, D'Souza G, Shad A, Sherlala K, Sidhu J, et al. Pineal germinoma presenting as anorexia nervosa: Case report and review of the literature. Int J Eat Disord. 2006; 39:606–8.

20. Crawford JR, Santi MR, Vezina G, Myseros JS, Keating RF, LaFond DA, et al. CNS germ cell tumor (CNSGCT) of childhood: presentation and delayed diagnosis. Neurology. 2007; 68:1668–73.

21. Andreu Martínez FJ, Martínez Mateu JM. Intracranial germ cell tumor mimicking anorexia nervosa. Clin Transl Oncol. 2006; 8:915–8.

22. Cole TJ, Flegal KM, Nicholls D, Jackson AA. Body mass index cut offs to define thinness in children and adolescents: international survey. BMJ. 2007; 28:194.

## A21 The “Accoglienza” therapy

### Lucia Celesti, Massimiliano Raponi

#### Medical Direction, Bambino Gesù Children’s Hospital (OPBG), 00165 Rome, Italy

##### **Correspondence:** Lucia Celesti (lucia.celesti@opbg.net)

Being sick is tough. It gets even tougher when a child is sick, because there are not only his own needs, but also the ones of his family. However, in Italy very few hospitals are organized with Family Services as essential factor in treatment along with Clinical and Research units, while OPBG Medical Direction offers diverse services specifically dedicated to answer the health needs by taking care of the families.

For starters, it is necessary to praise the all too often burdened front line service. The first problem for those who come from far away, is having a roof. The principal network is the one dedicated to needy families: the hospital offers free accommodation integrated with the Guardian Angel’s program: the family is followed by the same person during the entire process.

The Cultural mediation service has a double system: one that guarantees the presence of interpreters and one for immediate translation through a free phone number.

The psychosocial treatment of the child by Child life services is not an optional measure, but a primary part of the treatment process: during hospitalization, the help we give the child to cope with their fears also supports families and the health operators. Continuity of the scholastic itinerary must be guaranteed: inside the Hospital primary and secondary school lessons of every grade are provided.

Parents associations are a precious resource, both because of their direct knowledge of the assistance and psychological problems of the patients, and because of their capability to find empathic and trusted links with customers. The opinion of patients and families, which were already participating in all the Committees of the hospital, is taken into greater consideration thanks to the Councils composed of both relatives and teenagers.

In the last years the number of families supported by Social Services has enormously increased. The primary goal is to help families when they most need; also the hospital Counselors, specialized in supporting and listening to the family during difficult times, build relationships to help them.

The protective circle around the patient and his family is concluded with the analysis and verification of the effectiveness of our system. Customer satisfaction is systematically measured through different tools: surveys, complaints, and daily Speak-Up visits.

Family Services are meant to take care of the child and his family for all the non-clinical aspects of assistance, whatever fundamental: a real “therapy of care”[1].

**Reference**

1. Celesti L. Family Centered Care: the “Accoglienza” Therapy. MEDIC 2015; 23:24-33.

## A22 Fever management in children

### Elena Chiappini (echiappini@unifi.it)

#### Meyer University Hospital, Florence, Italy

Despite efforts for reducing fever phobia among physicians and parents/tutors, the management of the febrile child is still a major issue [1,2]. The Italian guidelines for managing fever in children for health care providers and parents/caregivers drafted by the Italian Pediatric Society have been updated with the aim to review the recent literature looking for new scientific evidences about challenging topics [1]. The main messages are: in children younger than 4 weeks: axillary temperature measurement using a digital thermometer is recommended. In children older than 4 weeks, in the hospital or ambulatory care setting, axillary temperature measurement using a digital thermometer or an infrared thermometer (tympanic or with or without skin contact) is recommended while at home axillary temperature measurement performed by tutors using a digital thermometer is recommended. Paracetamol and ibuprofen are the only antipyretic drugs recommended for use in children. Combined or alternate use of ibuprofen and paracetamol is not recommended. Ibuprofen should not be used in case of dehydration. Use of paracetamol or ibuprofen is not recommended to reduce the incidence of fever and local reactions in children undergoing vaccination. Preventive use of paracetamol or ibuprofen is not recommended for the prevention of febrile convulsions in children [1].

**References**

1. Chiappini E, Venturini E, Remaschi G, Principi N, Longhi R, Tovo PA, et al. Italian Pediatric Society Panel for the Management of Fever in Children. Update of the Italian Pediatric Society Guidelines for Management of fever in Children. J Pediatr 2017; 180:177-183.

2. Bertille N, Purssell E, Corrard F, Chiappini E, Chalumeau M. Fever phobia 35 years later: did we fail? Acta Pediatr 2016; 105:9-10.

## A23 New autoinflammatory diseases

### Rolando Cimaz, Maria Costanza Caparello

#### UOC Reumatologia, Ospedale A. Meyer, Università di Firenze, Florence, Italy

##### **Correspondence:** Rolando Cimaz (r.cimaz@meyer.it)

Autoinflammatory diseases were first defined in 1999 by Michael McDermott and Daniel Kastner as “conditions characterized by seemingly unprovoked episodes of inflammation, without high-titer of autoantibodies or antigen-specific T-cells” [1]. The concept of autoinflammatory diseases has evolved over the past years [2-4], with knowledge advancements on the pathogenic mechanisms underlying many autoinflammatory diseases and their genetic basis. The autoinflammatory syndromes are mainly monogenic diseases but also include some genetically complex disorders. Current knowledge underlines that autoinflammatory diseases comprise many disorders with high clinical variability and features of recurrent fever attacks, prevalence of innate immune system activation, in the absence of high titer of autoantibodies or antigen specific T cells. The classification of autoinflammatory diseases comprises: hereditary periodic fever syndromes (the most common and the most studied) and cryopyrinopathies, inflammasomopathies, NF-kB mediated disorders, interferonophaties, and others. Beside the best known FMF, HIDS, TRAPS, Muckle-Wells and CINCA, new entities have been recently discovered. These include STING associated vasculopathy with onset in infancy (SAVI), proteasome associated autoinflammatory syndromes (PRAAS), deficiency of adenosine deaminase 2 (DADA 2) [5,6], PLCG2 associated antibody deficiency and immune dysregulation (PLAID), PLCγ2-associated antibody deficiency and immune dysregulation (APLAID), haploinsufficiency of A20, Otulipenia [7], CANDLE, and autoinflammatory bone diseases eg CRMO and SAPHO syndrome [8]. Several of these disorders have features of immune deficiency associated with autoinflammation. Recent advances in the molecular and pathophysiological basis of autoinflammatory diseases have provided new treatment strategies. While most autoinflammatory disorders respond exceptionally well to IL-1 inhibition, exceptions exist such as anti-TNF for DADA2 and Jak inhibition for SAVI syndrome [9]. The field is advancing quickly and basic research is essential for elucidating the molecular basis and paving way for new treatments.

**References**

1. McDermott MF, Aksentijevich I, Galon J, McDermott EM, Ogunkolade BW, Centola M, et al. Germline mutations in the extracellular domains of the 55 kDa TNF receptor, TNFR1, define a family of dominantly inherited autoinflammatory syndromes. Cell 1999; 97:133–144.

2. Galon J, Aksentijevich I, McDermott MF, O’Shea JJ, Kastner DL. TNFRSF1A mutations and autoinflammatory syndromes. Curr Opin Immunol 2000; 12:479–486.

3. McGonagle D, McDermott MF. A proposed classification of the immunological diseases. PLoS Med 2006; 3:297.

4. Kastner DL, Aksentijevich I, Goldbach-Mansky R. Autoinflammatory disease reloaded: a clinical perspective. Cell 2010; 140:784–790.

5. Navon Elkan P, Pierce SB, Segel R, Walsh T, Barash J, Padeh S, et al. Mutant adenosine deaminase 2 in a polyarteritis nodosa vasculopathy. N Engl J Med 2014; 370:921-931.

6. Zhou Q, Yang D, Ombrello AK, Zavialov AV, Toro C, Zavialov AV, et al. Early-onset stroke and vasculopathy associated with mutations in ADA2. N Engl J Med 2014; 370:911-20.

7. Zhou Q, Yu X, Demirkaya E, Deuitch N. Biallelic hypomorphic mutations in a linear deubiquitinase define otulipenia, an early-onset autoinflammatory disease. Proc Natl Acad Sci USA 2016; 113:10127-32.

8. Taddio A, Zennaro F, Pastore S, Cimaz R. An update on the pathogenesis and treatment of Chronic Recurrent Multifocal Osteomyelitis in children. Paediatr Drugs 2017; 19:165-172.

9. Frémond ML, Rodero MP, Jeremiah N, Belot A, Jeziorski E, Duffy D, et al. Efficacy of the Janus kinase 1/2 inhibitor ruxolitinib in the treatment of vasculopathy associated with TMEM173-activating mutations in 3 children. J All Clin Immunol 2016; 138:1752-1755.

## A24 Luigi Somma at the Annunziata Sacred House in Naples

### Patrizia Cincinnati (patnati@mclink.net)

#### Study Group of the Italian Society of Pediatrics on the History of Pediatrics, Rome, Italy

In the second half of the nineteenth century nascent pediatrics in Italy is marked by the figure of Luigi Somma (1836-1884), physician at the Foundling Hospital of the Annunziata Sacred House in Naples. A review of his writings enabled us to emphasize what makes him a pioneer of the discipline. Luigi Somma’s publications have been sought through the National Library System’s catalog. Two large monographs have been so identified: Treaty of Hygiene for Foundling Hospitals (1871) and Pediatric Clinic at the Annunziata Foundling Hospital (1875), both entirely examined. Also the first Italian edition of the pediatric treaties by Roger (1873), Gerhardt (1880) and Stössl (1881) commented by Somma with several notes were examined. The research was completed by an exam of Archives of infant pathology, the first pediatric national journal founded in 1883 by Somma. Treaty of Hygiene consists of 8 chapters on microclimate, feeding, clothing, cleaning, sleep and physical activity suitable for children. Interestingly, the work is composed of hygienic issues associated with discussion on numerous infant diseases. Appendix II contains the first request to the Italian Government for setting up Clinics dedicated to children diseases. Pediatric Clinic consists of two parts: a Statistic one with 48 tabulated clinical cases and a second one consisting of Lectures starting from them and given in 1874 at the Pediatric Clinic just established at the Annunziata Foundling Hospital. The monograph is characterized by a practical approach to the sick infants and deals mainly with neonatal diseases, such as sclerema, trismus, and jaundice. Both the works prove the author’s notable attention for an exact semeiotics and an unequivocal nomenclature. Also his interest in pathophysiological mechanisms is evident together with consequent therapeutic options, such as warming of sclerematous newborns or milk administration by a small esophageal probe in infants with poor or absent sucking. The author's discussion of literature always moves from a personal and skilful experience. Lastly, the Archive’s exam shows the support of Somma to vaccination. His article "On the infant splenic anemia" (1883) is remarkable and starts a very fruitful line of pediatric research in Italy. Luigi Somma deeply changed contemporary approach to the sick child. A proponent of an accurate semeiotics and a supporter of pathophysiological studies, he was the first one to teach Pediatrics in Italy thus promoting the discipline in the country.

## A25 New Italians: citizenship and everyday multiculturalism among adolescent children of immigrants

### Enzo Colombo (enzo.colombo@unimi.it)

#### Department of Social and Political Sciences, Università degli studi di Milano, Milano, Italy

The growing presence of children of immigrants suggests a structural change in host societies. After 40 years of immigration, the children of immigrants – born and raised in Italy – constitute a significant share of 'new Italians'. It is not helpful to our understanding of the phenomenon to look at these young teenagers as 'immigrants'. They are an important and innovative part of a new generation of young people fully involved in the transformations created by globalization processes.

Based on multi-year research data on the subject, my presentation focuses on identification processes and the idea of belonging and citizenship of young children of immigrants attending high schools in Lombardy.

These young people, in large part, share the same daily experiences with their autochthonous companions, the same patterns of consumption, and the same expectations for the future. They consider the possibility of obtaining Italian citizenship a fundamental prerequisite to have equal opportunities and to play their own cards well. They consider the current Italian citizenship law – rigidly based on a principle of ius sanguinis – to be unjustifiably restrictive and discriminatory because it does not recognize them the right to participate in social and political life.

Adolescent children of immigrant – at least those among them who have high social and cultural capital and actively invest in their professional education – seem to represent a more widespread generational condition. They face the everyday experience of living the complexity and ambivalence of social contexts in which recognizing other’s difference, being able to translate from one language to another, finding mediations for avoiding exclusion, and relativizing rules, value and codes become necessary skills. In this way, they learn to manage ambivalence, multiplicity and change and are committed to finding new solutions to address the experience of growing globalization.

## A26 Humanization of the pediatric care: the experience of Campania Region

### Anna Giulia Elena De Anseris (annagiulia.deanseris@sangiovannieruggi.it)

#### Pediatrics, AOU “S. Giovanni di Dio e Ruggi d’Aragona”, 84100, Salerno, Italy

Humanization of the care has been shown to improve care quality, parental satisfaction level, and healthcare costs [1]. In a number of Italian Regions, difficult economic contexts have hitherto prevented:

1) the implementation of organic programs able to strengthen the chronically deficient and/or poorly distributed medical/nursing personnel and updated instrumental resources;

2) humanization of care policies, particularly in pediatric facilities.

These shortcomings are contributing also to increase the extra-regional health migration, as we have recently highlighted [2]. In order to overcome these disparities, in 2013 the State-Region Agreement issued a call for Regional Programs focusing on the "Development of Humanization Processes in Care Pathways" ( Section 8^th^), to reduce the variability between Regions and different Structures in the same Region, through the elaboration and adoption of national criteria.

In this context, the Campania Region started a Project in the pediatric field, namely called: "Analysis and Implementation of the Process of Humanization of the Care Pathways in the Pediatric Hospital Structures of the Campania Region".

In its pilot stage, the project involves a limited number of pediatric wards of the 7 main regional Hospitals, and is dealing with a number of actions (Figure 1) such as:

- assessment and monitoring of the degree of humanization of the pediatric care, by means of several measurement tools (AGENAS, LPCP tool, HPH-CA Health Promoting Hospitals & Health Services (HPH)- Child and Adolescents)

- creation and diffusion of medical and nursing staff training courses (eg pain, humanization, …),

- improvement of care strategies in hospital (eg, “born to read” sub-project, Child-friendly signage)

- creation of a dedicated web site (http://pedianetcampania.it/)and a web based platform, reciprocally interfacing pediatric wards with general pediatricians and patients’ families;

- implementation of a common teleconference/tele-consultation system;

The different phases of the Regional Project are supposed to be able:

* To identify the improvable areas of welcome, hospitalization and discharge of the little patients,

* To plan and produce measurable strategies of action through staff training

* To verify the effectiveness of short-, medium- and long-term measures.

Generally, this project had the ambition to change structurally the pediatric staff duties, to produce durable virtuous changes and serve as an exportable and reproducible model for future actions in pediatric humanization of care.

**References**

1. Aragon SJ, McGuinn L, Bavin SA, Gesell S. Does pediatric patient-centeredness affect family trust? J Healthc Qual. 2010; 32:23-31.

2. Vajro P, Paolella G, Celentano E, Longo S, Saccheri T, Pinto C, et al. Characterization and burden of Campania children health migration across Italian regions during years 2006-2010: chance and/or necessity? Ital J Pediatr. 2012; 38:58.

## A27 Being born in Italy today

### Mario De Curtis, Viviana Cardilli, Fabio Natale

#### Department of Pediatrics, La Sapienza University of Rome, Rome, Italy

##### **Correspondence:** Mario De Curtis (mario.decurtis@uniroma1.it)

**Background**

The analysis of demographic changes in a country is really important to provide more adequate maternal and child care.

**Materials and methods**

The data of the National Institute for Statistics (ISTAT), Certificate of attendance at birth (CeDAP) and Public Health Institute (ISS) were retrieved and analysed.

**Results**

In 2016 there were about 474.00 births, about 100,000 less than in 2008. Newborns born to foreign women were about 92,000 (19.4% of all births). Of these, 61.000 had a foreign father and 31.000 an Italian father. Foreign mothers who give birth in Italy come more frequently from Romania, Morocco, Albania, Ukraine and Poland. In recent years, there has been a significant increase in births from multiple pregnancies, which now account for about 3% of all births. The most recent data on infant mortality (deaths in the first year of life/1000 live births) shows in 2014 a value of 2.8 per 1000. Despite the satisfactory statistics on average, in Italy there is a significant disparity in mortality between northern and southern regions. The worst prognosis, recorded in southern regions (infant mortality rate is on average 30% higher than in central and northern regions) has not changed in the last 65 years. Equally, some diagnostic tests at birth, such as the expanded newborn screening with tandem mass spectrometry (MS/MS), are performed today only on about half of Italian newborns. The decrease in birth rate is driven by many factors, but economic considerations, related to the increase in poverty and youth unemployment, undoubtedly plays an important role. Linked to this aspect and also to the different role of women in the workforce and society, the last decades have seen an increase in the age of women at childbirth. Today more than a third of Italian women has her first child at an average age of 35 years and more than 8% over the age of 40. This phenomenon, associated with the increasing use of assisted reproductive technologies, has led to an increase in multiple pregnancies and preterm births. Despite the significant reduction in infant and child mortality rates, there are significant regional differences.

**Conclusions**

In Italy, perinatal organization needs to be improved in southern regions. It is essential to ensure that all women and their children have absolutely equal access to medical services during pregnancy and childbirth, with equal dignity and safety, with no differences in ethnicity and social status.

## A28 Investigation on the premonitory symptoms of FED in a school population (8-10 years old)

### Giampaolo De Luca^1^, Matteo Napoletani^2^, Vita Cupertino^3^, Riccardo De Lorenzo^3^

#### ^1^Family pediatrician, Gruppo di Studio Nazionale Adolescenza SIP, Cosenza, 87100, Italy; ^2^Medical student, Sapienza Università di Roma, Roma, 00185, Italy; ^3^Community pediatrician, Gruppo di Studio Adolescenza SIP, ASP Cosenza, 87100, Italy

##### **Correspondence:** Giampaolo De Luca (giampaolo60@libero.it)

**Background**

Feed and Eating Disorders (FED) are an expanding "social epidemic" [1]. The literature highlights a decrease in the onset of symptoms at 8 to 12 years [2]. A delay in diagnosis can worsen the prognosis of these disorders [3]. An investigation was conducted on the perception of the body's own image and on the use of low calorie diets in the first adolescence by providing a questionnaire with 4 questions extracted from diagnostic tests (EAT 26), usable by the Pediatrician to "alert" the FED [3]. On the other hand, the alarming rise in weight gain in evolutionary age leads to low-calorie diets, as therapeutic intervention, at an age where the onset of pathological behaviors can be favored in predetermined subjects.

**Materials and methods**

The survey was conducted on 783 pupils (397F-386M) of the fourth and fifth class of primary school, aged 8-10. The administration of the questionnaire (1. Do you feel dissatisfied with your body? 2. Does weight influence the idea that you have of yourself? 3. Do you think you should be dieting? 4. How many diets did you start in the last year?) was supported by the pediatrician along with the teacher, in a written and anonymous form, simplifying the questions but avoiding influencing the answers.

**Results**

The answers of the sample were in percentage: 35.50% declare dissatisfaction with their body weight, 36.65% declare that weight affects the idea of themselves, 33.71% believe they have to diet, 23.49% say they have started one or more diets over the past year. By analyzing the answers by age, no significant differences were observed for all four questions among the children of the fourth and fifth class of primary school, while observing percentages of slightly higher positive responses in the fifth class children (Fig. 1). Unlike expectations, there are no real differences between male and female responses at this age (Fig. 2).

**Fig. 1:**
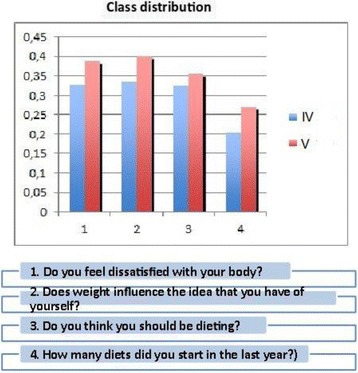
**(abstract A28).** Class distribution

**Fig. 2:**
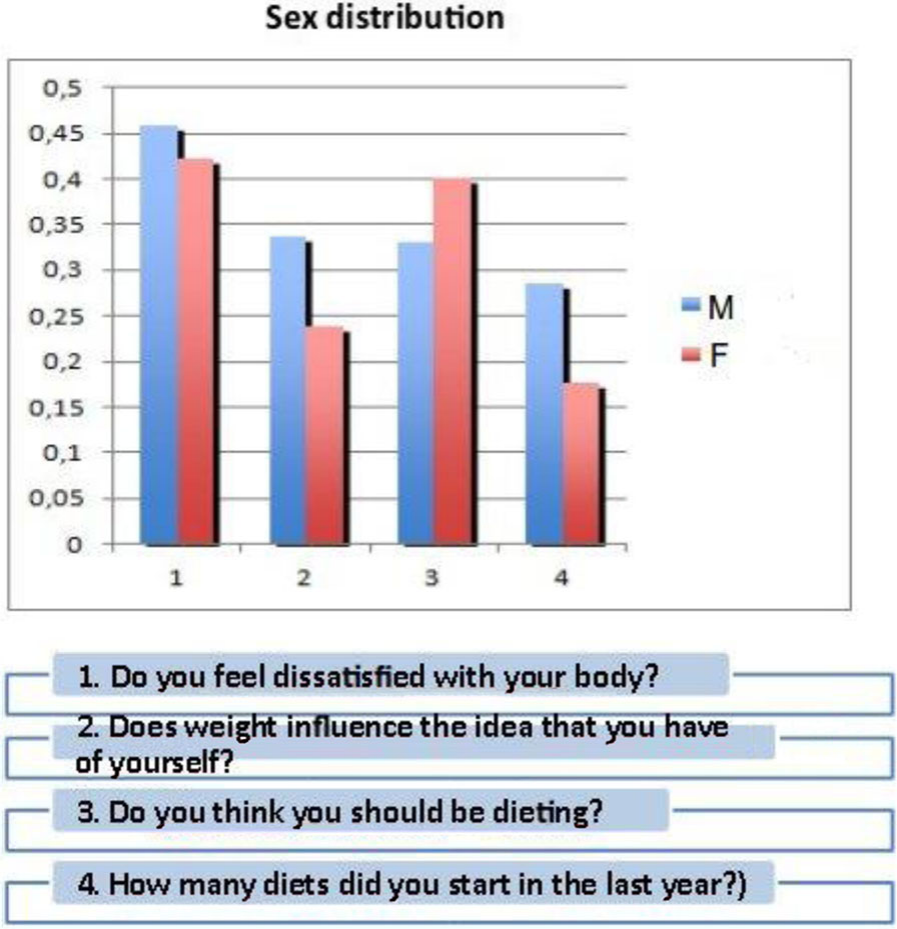
**(abstract A28).** Sex distribution

**Conclusions**

FED are particularly insidious at a time when contrast against weight excess can lead to the emphasis on less productive and often counterproductive therapeutic interventions. The school's survey can guide children's eating habits and perception of their own body image in early adolescence, which, if they are altered, can represent a premonition to FED.

**References**

1. Maestro S, Baroncelli GI, Ghione S, Bertelloni S. I disturbi del comportamento alimentare in adolescenza. Prospettive in pediatria. 2013; 170:74–83.

2. American Academy of Pediatrics; Committee on Adolescence. Identification and management of eating disorders in children and adolescents. Pediatrics. 2010; 126:1240–53.

3. De Luca G, Napoletani M. Premonitory symptoms of feeding and eating disorders in pediatric age. Ital J Pediatr. 2015; 41 (Suppl 2): A25.

## A29 The history of paediatrics in Naples: academic paediatrics

### Roberto Del Gado^1^, Antonio Navarra^1^, Paolo Montaldo^2^

#### ^1^Università degli studi della Campania "Luigi Vanvitelli", Naples, Italy; ^2^Centre for Perinatal Neuroscience, Imperial College London, London, United Kingdom

##### **Correspondence:** Roberto Del Gado (robertodelgado@virgilio.it)

The history of Paediatrics at in Naples has its roots in the Greek-Roman teaching and a few centuries afterwards, in the figure of Frederick II who laid the foundations of the Medical School in Naples. The Holy Roman Emperor was the first to realise the strategic importance of Naples as well as its pivotal role in facilitating the cultural growth of bright young students and scholars so as to avoid unnecessary and expensive trips to other cities for study reasons [1]. After the end of the Frederick II Empire, childcare was neglected until 1874 when Santa Casa dell’Annunziata started playing a leading role as a charitable institution in the city and a first unofficial attempt of teaching Paediatrics took place there. When the formation of the modern Italian state was completed, Chairs in Paediatrics came up in different cities including Naples, where Francesco Fede (1887-1913) became the first Professor in Paediatrics followed by Rocco Jemma (1913-1936), Luigi Auricchio (1936-1964) and Giulio Murano (1964-1980). In 1883 Francesco Fede founded “La Pediatria” a peer-reviewed paediatric medical journal which continued its activity for almost 100 years. This journal contributed to spread new ideas at a national and international level and helped to set new links within the academic field. Throughout the 19th century, infant mortality rates were huge and physicians considered "childhood diseases" to take up the biggest share in human pathology. A specific field in knowledge and clinical practice was gradually built, and under the initiative and leadership of Ernesto Cacace (Fede’s Medical School disciple) the basis of Nipiology came to the fore [2]. Consequently, the concept of "culture of prevention" emerged and Medicine entered a scientific era, which ultimately set the foundations of the "puericulture movement" (subsequently known as Social Paediatrics) and perinatal care. In 1925, following these new concepts, a special fund for maternity and child welfare was created (ONMI) as well as new hospitals and Paediatric Health Institutes for rachitic children [3]. At that time the Italian Society of Paediatrics was also founded and several National Conferences took place in Naples, highlighting the intense cultural role played by the Naples Medical School. In conclusion, Paediatric Medical School in Naples has clearly played an influential role in Italy. The History of Paediatrics at Naples reflects how quickly infant mortality decreased since the 19th century and how different nowadays are the “new diseases” which paediatricians have to challenge like rare and chronic diseases.

**References**

1. De Renzi S, Napoli M. Stato della medicina nel resto d’Italia durante il periodo salernitano. In: Ripostes editor. Storia della medicina in Italia: la scuola di Salerno. 2nd edition. Naples; 2000. p.195-213.

2. Tinanoff N. Development and developmental anomalies of the teeth. In: Kliegman RM, editors. Nelson Textbook of Pediatrics. 19th edition. Philadelphia: Elsevier Saunders; 2011. p.1250-1251.

3. Auricchio L. L'Università per la difesa dell'infanzia. Annali dell'Università italiana. 1940; 1:1-15.

## A30 The path to adulthood with hereditary metabolic disorders

### Roberto Della Casa^1^, Simona Fecarotta^1^, Cinzia Gragnaniello^1^, Miriam Massese^1^, Brunella Capaldo^2^

#### ^1^Department of Translational Medical Sciences, Section of Pediatrics, Federico II University, Naples, Italy; ^2^Department of Clinical Medicine and Surgery, Federico II University, Naples, Italy

##### **Correspondence:** Roberto Della Casa (roberto.dellacasa@unina.it)

The transition of patients with Inborn errors of metabolism (IEM) is a delicate matter; as the complexity of these disorders makes it difficult to identify a single specialist for the adult patient [1]. In Italy, the transitional pathways are mostly constructed on a voluntary basis and with various approaches: we wanted to identify one possible path for patients with IEM. Taking inspiration from the available literature, mainly related to Diabetes [2-3], 3 years ago AOU Federico II activated a transitional clinic with monthly appointments, involving 2 Metabolic Pediatricians, 1 Adult Physician and some support figures (nutritionist, psychologist, organizational figure). It started with a 13-month pilot project that involved only patients with hepatic glycogenosis, referring them to an internist expert of glucose metabolism disorders, with a paper and digital dossier summarizing the pathology and clinical history of the patient. 16 patients were involved (7M-9F, 3 in 14-17 years, 13> 18 years), 11 with GSD1a, 3 with GSD1b and 2 with GSDIIIa. Through a guided interview and psychological support, we observed how the initial sense of mistrust and anxiety was reduced with incremental visits, while also improving autonomy. At the end of the project, 3 patients were followed in DH Clinical Medicine, 6 in joint surgery, 4 in pediatric outpatient clinics, and 3 patients <18 years old co-operated with joint DHD pediatric surgery. All showed stability/improvement of clinical parameters and a high degree of satisfaction. Based on this experience, it was decided to extend the project: in the last 18 months, 29 visits were made to 19 patients, all >18 years old(8M-11F), of which 10 with hepatic glycogenosis, 6 with accumulation diseases (Gaucher, mucopolysaccharidosis, mucolipidosis), 2 with neuromuscular diseases (GSDIIIb, GSDV) and one OCT deficit. We concomitantly extended our network of consultants, now comprising: gastroenterologist, neurologist, rheumatologist, cardiologist and nephrologist. To date, 10 of these patients have been admitted to adult departments, 2 patients still hold periodic meetings in a joint outpatient clinic, 9 are still only in a joint outpatient clinic, although one patient has been admitted to the Medical Clinic. While for some IEM it is easier to find an adult patient specialist (e.g. a nephrologist for Fabry's disease, etc.), in other cases, due to the complexity of events or individual and sociocultural features, this process needs greater support. We believe that the joint outpatient clinic can be an excellent strategy to help these patients in acquiring full awareness and autonomy in managing their own pathology and interacting with their attendant physician, and we propose to maintain and enhance this promising clinical structure by further enlarging the network of specialists while seeking greater involvement with doctors in the area.

References

1. Tran C, Barbey F, Pitteloud N, Philippe J, Kern I, Bonafé L. Inborn errors of metabolism: transition from childhood to adulthood. Rev Med Suisse. 2015; 11:445-449.

2. Garvey KC, Markowitz JT, Laffel LM. Transition to adult care for youth with type 1 diabetes. Curr Diab Rep. 2012; 12:533–54.

3. Van Walleghem N, Macdonald CA, Dean HJ. Evaluation of a systems navigator model for transition from pediatric to adult care for young adults with type 1 diabetes. Diabetes Care. 2008; 31:1529-30.

## A31 PANDAS: tip of the iceberg

### Raffaele Falsaperla^1^, Marco A N Saporito^2^, Valeria Di Stefano^3^, Piero Pavone^1^

#### ^1^Acute and Emergency Paediatric and General Paediatric Operative Unit, Policlinico-Vittorio Emanuele Hospital, University of Catania, Catania, Italy; ^2^Neonatal Intensive Care Unit, Santo Bambino Hospital, Policlinico-Vittorio Emanuele Hospital, University of Catania, Catania, Italy; ^3^Paediatric Unit, Lentini Hospital, Lentini (Siracusa), Italy

##### **Correspondence:** Raffaele Falsaperla (raffaelefalsaperla@hotmail.com)

**Background**

“Pediatric Autoimmune Neuropsychiatric Disorders Associated with Streptococcal Infections” (PANDAS) are conditions at pediatric acute onset characterized by obsessive compulsive disorder (OCD), tic disorders and cognitive manifestations following a group A beta-haemolytic streptococcal infection (GABHSI) [1]. The term PANDAS was first used by Swedo et al in 1998. Pathogenesis is not clear; it is due to anti-neuronal autoantibodies against brain antigens induced by GABHSI. Diagnostic criteria are: 1) presence of OCD and/or a tic disorder; 2) pediatric onset; 3) acute clinical onset and episodic course; 4) temporal association with GABHSI; 5) association with neurological abnormalities (motor hyperactivity and choreiform movements) [1,2]. PANDAS have not been accepted as distinct disorder; there are not specific tests or diagnostic biomarkers [2]. Recently it has been performed a revision of diagnostic criteria and it has been proposed a new clinical entity, the Pediatric Acute onset Neuropsychiatric Syndrome (PANS) to indicate a subtype of OCD with an acute and dramatic onset or exacerbation with multiple aetiologies. Therapy consist in use of antibiotics, anti-inflammatory, intravenous immunoglobulin, plasma exchange, drugs for movement disorders and neuropsychiatric alterations, cognitive-behavioural therapies and tonsillectomy [1,2].

**Materials and methods**

To identify articles concerning PANDAS and PANS syndrome. The PubMed database was used to search and typed terms were as follow: PANDAS syndrome, PANS syndrome, PANDAS syndrome diagnosis, PANS syndrome diagnosis, PANDAS treatment, PANS treatment, pediatric autoimmune neuropsychiatric disorders associated with streptococcal infections, pediatric acute onset neuropsychiatric syndrome, movement disorders associated to streptococcal infection.

**Results**

We found 160 articles. We selected only articles in English language concerning diagnosis and treatment. Case reports were 55. We selected randomized controlled trial (RCT), systematic reviews, reviews, observational studies, clinical studies. We found 38 reviews and 4 systematic reviews. Comparative studies were 6. We found 5 RCT, 3 about antibiotic therapy, 2 about cognitive-behavioural therapy. We didn’t find meta-analysis or guideline. We found a survey published in 2017 concerning PANS characteristic and course in 698 patients, but we didn’t consider it because authors included adult patients. We found an observational study concerning surgery. There isn’t widespread agreement on tonsillectomy. Singles cases are reported concerning the resolution of symptoms after surgery. In this study Pavone et al provided that surgery didn’t improve the course of the disease.

**Conclusions**

PANDAS are a very heterogeneous condition. Diagnosis and treatment are debated topics and Literature data are controversial. Further studies are necessary to a better definition and management of this condition.

**References**

1. Williams KA, Swedo SE. Post-infectious autoimmune disorders: sydenham chorea, PANDAS and beyond. Brain Res. 2014; 1617:144-154.

2. Pavone P, Rapisarda V, Serra A, Nicita F, Spalice A, Parano E, et al. Pediatric autoimmune neuropsychiatric disorder associated with group A streptococcal infection: the role of surgical treatment. Int J Immunopathol Pharmacol. 2014; 27:371-8.

## A32 Management of acute bronchiolitis in the pediatric ward

### Giovanna Gaudiello, Edoardo Vassallo, Antonio Correra

#### 3 Pediatric Unit, Department of Pediatrics, Santobono Children’s Hospital, Naples, Italy

##### **Correspondence:** Giovanna Gaudiello (giogaudie@libero.it)

Bronchiolitis is an acute inflammatory disease of the lower respiratory tract. It is characterized by acute inflammation, edema, and necrosis of epithelial cells lining small airways, increased mucus production, and bronchospasm. It is the most common lower respiratory tract infection in the first year of life. Bronchiolitis is the first cause of hospitalizations in infants less than 1 year of age.

Respiratory syncytial virus (RSV) is the most common etiologic agent of bronchiolitis. The severity of RSV-related disease is worse than that of other viral infections.

Most infants who contract bronchiolitis recover without sequelae; instead, some infants require intensive care and respiratory support. Younger infants and those with pre-existing risk factors (prematurity, bronchopulmonary dysplasia, congenital heart diseases and immunodeficiency) are more likely to develop a severe form of respiratory disease.

Despite the high incidence of bronchiolitis, confusion and controversies still exist about its definition and management. To date, there is no specific treatment for viral bronchiolitis, and the mainstay of therapy is supportive care. Typical bronchiolitis in infants is a self-limited disease that is little modified by treatment. Many clinical guidelines have shown that corticosteroids and bronchodilator have little or no effect on the clinical evolution of the disease. Nebulized adrenaline may be sometimes useful in the emergency room, but in the hospital setting do not change severity of disease or length of stay.

It is suggested that nebulized hypertonic saline may be useful in hospitalized bronchiolitis, making secretion less viscous and promoting their excretion. Antibiotics are not recommended unless clinical features or laboratory results indicate secondary bacterial infection [1,2,3].

The main points of the management of viral bronchiolitis are oxygen and rehydration support. Use of high-flow nasal cannula provides superior results to low flow oxygen delivery in moderate to severe bronchiolitis. High Flow Nasal Cannula (HFNC) therapy has emerged as a new method to provide humidified air flow to deliver a non-invasive form of positive pressure support. This treatment can be used in pediatric ward and reduces transferring of infants with bronchiolitis in intensive care unit. Some studies suggest that HFNC reduces the need of intubation and the rate of mortality [4,5].

**References**

1. Ralston SL, Lieberthal AS, Meissner HC, Alverson BK, Baley JE, Gadomski AM, et al. Clinical practice guideline: the diagnosis, management, and prevention of bronchiolitis. Pediatrics. 2014; 134:1474-1502.

2. NICE guideline. Bronchiolitis in children: diagnosis and management. 2015. Available in https://www.nice.org.uk/guidance/ng9. Accessed in June 22, 2017.

3. Baraldi E, Lanari M, Manzoni P, Rossi GA, Vandini S, Rimini A, et al. Inter-society consensus document on treatment and prevention of bronchiolitis in newborns and infants. Ital J Pediatr. 2014; 24: 40:65.

4. Cunningham S, Fernandes RM. High-flow oxygen therapy in acute bronchiolitis. Lancet. 2017. 4; 389:886-887.

5. Wing R, James C, Maranda LS, Armsby CC. Use of the high-flow nasal cannula support in the emergency department reduces the need for intubation in pediatric acute respiratory insufficiency. Pediatr Emergency Care. 2012; 28:1117-23.

## A33 How to write a scientific article & tips of scientific English

### Jean A. Gilder (info@jeangilder.it)

#### Scientific Communication srl, Naples, Italy

Publishing in peer-reviewed journals is becoming increasingly competitive. Frequently, papers are rejected by journals because of poor data presentation. Consequently, it is now recognized that the formation of researchers and physicians should include specific training in how to write a biomedical article. This presentation focuses on the issues faced by non native English speakers when writing scientific articles for peer-reviewed journals. The presentation is divided into two main parts.

1) Structure: (a) The title and the abstract represent the "business card" of your article, and its most widely read parts. A good title and abstract will favorably impress the journal editor, the reviewer and readers. (b) The body of scientific articles typically follows the IMRAD structure (Introduction, Materials and Methods, Results, Discussion). The items to include (or most importantly, not to include) in each section will be briefly illustrated.

2) Language: Language may be a barrier to publication, especially for not native English speakers. The seminar will provide tips to help you to avoid the most common pitfalls and mistakes made by Italian investigators when writing in English.

## A34 New communications systems for the therapeutic education of adolescents with type 1 diabetes: from chat lines to a mobile chat APP

### Dario Iafusco^1^, Angela Zanfardino^1^, Santino Confetto^1^, Crescenzo Cascella^2^, Antonietta Chianese^2^, Benedetta Rosaria Totaro^1^, Alessandra Cocca^1^, Laura Perrone^1^ and Alda Troncone^2^

#### ^1^Regional Centre of Pediatric Diabetology “G. Stoppoloni”, Department of the Woman, of the Child and of the General and Specialized Surgery, University of Campania "Luigi Vanvitelli”, Naples, Italy; ^2^Department of Psychology, University of Campania “Luigi Vanvitelli”, Caserta, Italy

##### **Correspondence:** Dario Iafusco (dario.iafusco@uninacampania.it)

As the recent revolution of transformative communications technology now connects individuals to the entire world, remote communications are progressively replacing face-to-face meetings. The use of social media networks has not only become increasingly popular, but also been associated with the enhanced well-being and social support of individuals with chronic disease [1, 2]. As using mobile phones for telephone calls declines, using them as smartphones—low-cost devices with the potential for continuous Internet access—is on the rise. Today’s young people, almost always online, have adopted virtual means to sustain their interpersonal relationships. For adolescents facing physical and psychological changes, along with typical difficulties of peer integration, communications via SMS and chat platforms, which allow delayed responses, do not require face-to-face interaction, and permit them to present themselves differently from their real-life selves, have become a highly popular way to keep in touch with friends. In response to that trend and the specific challenges of adolescents with type 1 diabetes (T1DM) always waging personal battles against the disease’s restrictions, the Regional Center for Pediatric Diabetology "G. Stoppoloni" in Naples (Italy) adopted new communications systems for educational purposes in 2000 by opening a chat line for adolescent patients. Using the chat line, accessible by personal computer during certain hours every week, adolescents discussed their chief concerns regarding T1DM and shared aspects of their daily lives. Patients joined the virtual community by using aliases that allowed them to anonymously express their fears and problems. Participation improved patients’ adherence to treatment, their degree of metabolic control, and the autonomous management of T1DM [3, 4].

In 2016, to offer a more attractive opportunity of virtual contact, the Diabetology Centre proposed an updated version of the chat line downloadable as a smartphone app. Named “L’isola pancreatica che non c’è”, the virtual group affords adolescent patients with T1DM a space to chat and interact with friends using multimedia content (e.g., vocal messages, photos, stickers). Interestingly, participants no longer preferred to use aliases, but often use their real names, even when discussing personal issues. They are becoming protagonists in their life stories, even to the extent that they have recently proposed to start a blog to share their experiences and organize meetings with teachers and classmates at school to explain important problems related to their struggles with T1DM.

**Acknowledgements**

Many thanks to all adolescents attending the Regional Center for Pediatric Diabetology "G. Stoppoloni" in Naples (Italy).

**References**

1. Troncone A, Cascella C, Chianese A, Iafusco D. Using computerized text analysis to assess communication within an Italian type 1 diabetes Facebook group. Health Psychol Open. 2015; 2:1-9.

2. Troncone A, Cascella C, Chianese A, Iafusco D. What relatives and caregivers of children with type 1 diabetes talk about: Preliminary results from computerized text analysis of messages posted on the Italian Facebook diabetes group. In: Bassis S, Esposito A, Morabito F, Pasero E, editors. Neural Networks and Computational intelligence for Information Communication Technologies. Cham: Springer International Publishing; 2016. p.235-242.

3. Iafusco D, Ingenito N, Prisco F. The chatline as a communication and educational tool in adolescents with insulin-dependent diabetes: preliminary observations. Diabetes Care. 2000; 23:1853.

4. Iafusco D, Galderisi A, Nocerino I, Cocca A, Zuccotti G, Prisco F, et al. Chat line for adolescents with type 1 diabetes: a useful tool to improve coping with diabetes: a 2-year follow-up study. Diabetes Technol Ther. 2011; 13:551-5.

## A35 Vaccination coverage for Papillomavirus (HPV) in Italy: state of art and strategies to address vaccine hesitancy

### Giancarlo Icardi, Cecilia Trucchi, Francesca Maria Piazza, Federico Tassinari

#### Department of Health Sciences, University of Italy, Genoa, 16132, Italy

##### **Correspondence:** Giancarlo Icardi (icardi@unige.it)

Since the licensures of anti-HPV quadrivalent (QIV) and bivalent vaccines in 2006, their indications have been progressively extended and the schedule indicated to pre-adolescents was modified [1,2].

In June 2015, the European Commission authorized the licensure of a nine-valent vaccine able to prevent 90% of malignancies HPV-related and almost 100% of condylomatosis [3].

In 2007, the Ministry of Health published the first national recommendations, identifying pre-adolescent females as primary target. All the Italian regions immediately adopted them [4]. The 2012-2014 National Immunization Prevention Plan (NIPP) [5] re-modulated the objective of vaccination coverage (VC) set for the birth cohort 2001 from ≥95% to ≥70%, introduced further objectives for the birth cohorts 2002 and 2003 (≥80% and ≥95%, respectively) and suggested a multi-cohort approach to female subjects, that was variously adopted by the regions. The 2017-2019 NIPP [6] aligns the VC objectives at ≥95% for all the female birth cohorts and introduces the recommendations for pre-adolescent males and at risk subjects. Nevertheless, seven regions and two local health units previously introduced the recommendations for pre-adolescent males and four regions for at risk subjects.

The most recent data about national VC obtained in the primary target showed a decreasing trend from 71.5% for the birth cohort 1999 to 56.3% for the birth cohort 2003, being largely under the objectives set by the Ministry of Health [7]. At a regional level, a wide variability of VC was observed both in the primary and in the secondary target.

The alarming decrease of VC have to be hindered through strategies that address the vaccine hesitancy (VH). Among the determinants of HPV-vaccine acceptance the health care provider and parents’ recommendations, the doubts about need and safety of vaccines and organizational criticisms such as the dynamic evolution of recommendations and objectives play a crucial role [8]. The main interventions to address VH are listening to doubts and concerns of pre-adolescents and their parents, building trust in health care workers and institutions, the education of both target subjects and primary care physician and logistical interventions [9,10].

Available evidences demonstrate that licensed HPV-vaccines are efficacious and safe [1,2]. Nevertheless, the dynamic evolution of indications, schedule, and immunization strategies may have contributed to suboptimal VC obtained both in primary and secondary target. The implementation of interventions to address VH is crucial to reach the objectives set by the NIPP, in order to improve equity and accessibility in the prevention field.

References

1. European Medicines Agency website. Gardasil. Available at: http://www.ema.europa.eu/docs/it_IT/document_library/EPAR_-_Product_Information/human/000703/WC500021142.pdf. Accessed May 10, 2017.

2. European Medicines Agency website. Cervarix. Available at: http://www.ema.europa.eu/docs/it_IT/document_library/EPAR_-_Product_Information/human/000721/WC500024632.pdf. Accessed May 10, 2017.

3. European Medicines Agency website. Gardasil9. Available at: http://www.ema.europa.eu/ema/index.jsp?curl=pages/medicines/human/medicines/003852/human_med_001863.jsp&mid=WC0b01ac058001d124. Accessed May 10, 2017.

4. Presidenza del Consiglio dei Ministri. Conferenza permanente per i rapporti tra lo stato, le regioni e le province autonome di Trento e Bolzano. Intesa, ai sensi dell’articolo 8, comma 6, della legge 5 giugno 2003, n. 131, tra il Governo, le Regioni e le Province autonome di Trento e Bolzano concernente “Strategia per l’offerta attiva del vaccino contro l’infezione da HPV in Italia”. Available at: http://www.statoregioni.it/Documenti/DOC_016696_264%20csr.pdf. Accessed May 10, 2017.

5. Ministero della Salute. Piano Nazionale Prevenzione Vaccinale (PNPV) 2012-2014. Available at: http://www.salute.gov.it/imgs/C_17_pubblicazioni_1721_allegato.pdf. Accessed May 10, 2017.

6. Ministero della Salute. Piano Nazionale Prevenzione Vaccinale (PNPV) 2017-2019. Available at: http://www.salute.gov.it/imgs/C_17_pubblicazioni_2571_allegato.pdf. Accessed May 10, 2017.

7. Ministero della Salute. Coperture vaccinali al 31/12/2015 per HPV (Aggiornamento 13 febbraio 2017). Available at: http://www.salute.gov.it/imgs/C_17_tavole_27_allegati_iitemAllegati_0_fileAllegati_itemFile_0_file.pdf. Accessed May 10, 2017. Accessed May 10, 2017.

8. Bailey HH, Chuang LT, duPont NC, Eng C, Foxhall LE, Merrill JK et al. American Society of Clinical Oncology Statement: Human Papillomavirus Vaccination for Cancer Prevention. J Clin Oncol. 2016; 34:1803-12.

9. Bratic JS, Seyferth ER, Bocchini JA Jr. Update on barriers to human papillomavirus vaccination and effective strategies to promote vaccine acceptance. Curr Opin Pediatr. 2016; 28:407-12.

10. Jarrett C, Wilson R, O'Leary M, Eckersberger E, Larson HJ; SAGE Working Group on Vaccine Hesitancy. Strategies for addressing vaccine hesitancy - A systematic review. Vaccine. 2015; 33:4180-90.

## A36 Italian Guideline on the acute asthma attack in children

### Luciana Indinnimeo^1^, Elena Chiappini^2^, Michele Miraglia del Giudice^3^ and the Italian Panel for the management of acute asthma attack in children^1^

#### ^1^Pediatric Department “Sapienza” University of Rome, Rome, Italy; ^2^Pediatric Infectious Disease Unit, Anna Meyer Children’s University Hospital, Florence, Italy; ^3^Department of Woman and Child and General and Specialized Surgery, University of Campania Luigi Vanvitelli, Naples Italy

##### **Correspondence:** Luciana Indinnimeo (luciana.indinnimeo@uniroma1.it)

**Background**

Acute asthma attack is a frequent condition in children. It is one of the most common reasons for emergency department (ED) visit and hospitalization. Appropriate care is fundamental, considering both the high prevalence of asthma in children, and its life-threatening risks.

The multidisciplinary panel of Italian Society of Pediatrics (ISP) recently issued a new guideline on the management of acute asthma attack in children over age 2, in ambulatory and emergency department settings.

**Materials and methods**

The Grading of Recommendations Assessment, Development, and Evaluation (GRADE) methodology was adopted. A literature search was performed using the Cochrane Library and Medline/PubMed databases, retrieving studies in English or Italian and including children over age 2 year.

**Results**

Inhaled ß_2_ agonists are the first line drugs for acute asthma attack in children. Ipratropium bromide should be added in moderate/severe attacks. Early use of steroids is associated with reduced risk of ED visits and hospitalization. A 3-5 day course of oral prednisolone is preferable in children able to retain drugs orally. Leukotriene receptor antagonists should not be used. Aminophylline use should be avoided in mild/moderate attacks. Weak evidence supports its use in life-threatening attacks. Intravenous (iv) magnesium solphate could be used in children with severe attacks and/or forced expiratory volume1 (FEV1) lower than 60% predicted, unresponsive to initial inhaled therapy. Heliox could be administered in life-threatening attacks despite previous treatment.

**Conclusions**

This Guideline is expected to be a useful resource in managing acute asthma attacks in children over age 2.

**Acknowledgments**

Italian Panel for the management of acute asthma attack in children: Bernardini Roberto (Empoli), Capristo Carlo (Naples), Cardinale Fabio (Bari), Cazzato Salvatore (Ancona), Chiamenti Giampiero (Verona), Chinellato Iolanda (Taranto), Corsello Giovanni (Palermo), Cutrera Renato (Rome), Da Dalt Liviana (Padova), Duse Marzia (Rome), Festini Filippo (Florence), Frateiacci Sandra (Rome), Minasi Domenico (Polistena-Reggio Calabria), Novelli Andrea (Florence), Piacentini Giorgio (Verona), Scoppi Pietro (Spoleto-Perugia), Tappi Eleonora (Turin).

## A37 Methods and tools for the participatory assessment of the level of patient centeredness in health facilities

### Alessandro Lamanna (lamanna@agenas.it)

#### Quality and Accreditation Department, Agenas, Roma, Italy

The Unified Conference between the State, the Regions and the Autonomous Provinces of September 20^th^ 2007 assigned to Agenas a twofold mandate. On the one hand, promoting citizens, patients, professionals, organization and community empowerment within the regional healthcare services [2]. On the other hand, monitoring quality, equity and efficiency of the healthcare system.

Further background documents are:

- The Patto per la Salute (Pact for Health) 2014-2016, which highlights patient centeredness as a key element to ensure the care provided takes into account a person’s physical, psychological and social entirety
- Two State-Regions Conference Agreements (December 20^th^ 2012 and February 2^nd^ 2015), which defines patient centeredness as one of the 8 requirements for the new National Accreditation System.

Agenas has been promoting since 2011 a program aimed at improving quality of healthcare through the participatory assessment of patient centeredness in hospital facilities, carried out in cooperation with the Active Citizenship Network and the Italian Regions [2].. The method used- drawing on the experience of the Civic Audit [2] and based on the principles of external evaluation of quality- provides for the active participation of citizens in all the steps of the assessment process: from definition/development of the items included in the checklist to data collection (carried out by equipes composed of both citizens and professionals from the hospital facility) and finally to analysis of the results achieved and definition/implementation and monitoring of subsequent improvement actions.

The 4 areas assessed are [2]:

1. Person-oriented organizational and care processes

2. Physical accessibility livability and comfort of care facilities

3. Access to information, streamlining and transparency

4. Taking care of the relationship with the patient/citizen

The first participatory assessment, carried out in 2014, saw the voluntary participation of professionals from 287 hospital facilities and about 300 patient associations. A new edition is currently underway, with the aim of extending the assessment to all the hospital facilities. Of the 142 items included in the checklist, 16 are aimed at verifying the commitment of the health facilities on issues regarding patient centeredness of healthcare services for children and adolescents.

On this basis, within the Project “Analysis and implementation of patient centeredness processes in pediatric health facilities of the Campania Region”, an ad hoc checklist composed of 122 items, aimed at identifying elements considered as indicative of patient centeredness for pediatric patients and their families, 63 of which are specific for pediatrics.

**References**

1. Lamanna A, Tanese A, Metastasio R. Uno strumento per valutare il grado di umanizzazione delle strutture di ricovero. Monitor. 2013; 32:26-41.

2. Caracci G, Carzaniga S. Definizione, modello di analisi, strumenti ed esperienze significative di empowerment in sanità. I quaderni di Monitor 2010; 6:10-17.

## A38 The role of neonatologists in promoting vaccinations

### Gianluca Lista^1^, Ilia Bresesti^1^, Paolo Tagliabue^2^

#### ^1^NICU “V. Buzzi” Children’s hospital, ASST FBF-Sacco, Milan, Italy; ^2^NICU “San Gerardo” hospital, MBBM Foundation, Monza, Italy

##### **Correspondence:** Gianluca Lista (gianluca.lista@asst-fbf-sacco.it)

Vaccinations are worldwide recognized as the most effective large scale measure which has been playing a crucial role in dramatically reducing infants’ mortality [1].

However, even if safety concerns about vaccines are proved to be unreasonable and their efficacy has been widely confirmed, many children do not get vaccinated [2].

Moreover, lately social media have had purported role in spreading anti-vaccine attitude and advocate risk becoming a global phenomenon that could impact immunization [3].

Preterm infants are especially vulnerable to infectious diseases, but this population often experiences delays in vaccinations [4,5].

The successful implementation of vaccines depends on many factors.

Giving parents information so that they can make informed decisions about their children’s health is an important part of this process. In relation to this aspect, neonatologists are directly involved, regardless they work with term babies or premature infants.

It has been proved that healthcare providers’ attitude towards vaccination are among the most important influences on the decision to vaccinate [6].

A recent survey conducted during November and December 2016 and promoted by Italian Neonatal Society among all neonatologist of Lombardy (Italy) revealed that the physicians’ adherence to recommendations needs to be improved, particularly regarding new vaccines such as rotavirus.

Since we found that more than 50% of the surveyed doctors are 30-50 years, we have speculated that this age target hasn’t experienced the effects of epidemic diseases, and this could contribute to a lower awareness of the danger related to the decrease of “herd immunity”.

Another relevant finding regards the physicians’ perception that there is paucity of updated and easily-available information regarding vaccines, which leads to a perceived lower level of knowledge in this field.

Hence, we believe that a close cooperation between scientific societies and pharmaceutical industries could fill this gap.

To conclude, the role of physicians seems to be crucial in providing a widespread vaccination educational campaign, and neonatologist are involved from birth. In addition, an adequate training for doctors should be provided periodically and with easy access.

There is an urgent need for future research on vaccinations in preterm infants to further reinforce the safety and efficacy of vaccines and for an effective policy to implement the adherence to vaccinations national program.

**References**

1. Bland J, Clements J. Protecting the world's children: the story of WHO's immunization programme. World Health Forum. 1998;19:162-73.

2. My C, Danchin M, Willaby HW, Pemberton S, Leask J. Parental attitudes, beliefs, behaviours and concerns towards childhood vaccinations in Australia: A national online survey. Aust Fam Physician. 2017;46:145-51.

3. Kata A. A postmodern Pandora's box: anti-vaccination misinformation on the Internet. Vaccine. 2010;28:1709-16.

4. Cuna A, Winter L. Quality improvement project to reduce delayed vaccinations in preterm infants. Adv Neonatal Care. 2017. Apr 3. doi: 10.1097/ANC.0000000000000398. [Epub ahead of print].

5. Crawford NW, Yeo V, Hunt RW, Barfield C, Gelbart B, Buttery JP. Immunisation practices in infants born prematurely: neonatologists' survey and clinical audit. J Paediatr Child Health. 2009;45:602-9.

6. Doherty M, Schmidt-Ott R, Santos JI, Stanberry LR, Hofstetter AM, Rosenthal SL, et al. Vaccination of special populations: Protecting the vulnerable. Vaccine. 2016;34:6681-90.

## A39 Physical activity in the adolescent with type 1 diabetes

### Fortunato Lombardo, Claudia Ventrici, Giuseppina Salzano

#### Department of Pediatrics, University Hospital of Messina, Messina, 98125, Italy

##### **Correspondence:** Fortunato Lombardo (Fortunato.Lombardo@unime.it)

Physical activity is one of the three main pillars of type 1 diabetes (T1D) therapy, and if practiced since the early years of life, the child can gain control of his muscular movements, develop strength, and self-confidence.

Exercise enhances the hypoglycemic effects of insulin therapy, decreasing the daily insulin dosage, promoting blood pressure control, increasing glucose utilization, improving quality of life, prolonging life expectancy and psycho-physical well-being of the child and adolescent with T1D.

It is important to make distinctions between what is meant by physical activity, sport, and exercise, because management is different.

Physical activity is all about the body movement produced by the skeletal muscle contraction and which requires excess energy expenditure compared to the restive energy expenditure.

For sports it is all about the programmed, structured and repeated body movement in order to improve one or more components in good physical shape.

For sport, however, is meant everything that implies both fun and agonism.

Physical exercise in a child/teenager with diabetes is related to health conditions but also to good personal and family participation.

All management will be a constant balance dependent not only on the type of activity but not by agonistic or agonistic, whether aerobic or anaerobic, but also by many other factors such as the type of therapy the patient practices (eg multiinjective or microinfusion), the duration of physical activity and especially the type of diet that the patient follows.

It is the team's task to always give precise and safe messages and especially practical advice such as:

a) never practicing physical activity yourself;

b) Always schedule physical activity whenever possible, considering the type and duration

c) Always have sugar and glucagon available,

d) Monitor blood glucose, and

e) Always warn your friends and your own trainer about your health state.

## A40 Adolescents in international adoptions: complexity and specificity

### Giorgio Macario (macario.g@gmail.com)

#### Giudice Onorario presso il Tribunale per i Minorenni di Genova, Genova, Italia

The topic of adolescence in international adoptions has been dealt with in the past on a few specific occasions, but it emerges now strongly from the combination of over 50,000 adoptions realized from 2000 until today and from an average age of children adopted by now around 6 years old.

We will highlight this topic with a triple visual look. First of all, a short overview of the training at national level for practitioners dealing with international adoptions, in which specific issues related to adoptive adolescence have been developed [1]. The vast number of adopted children that have been entering the adolescence has driven practitioners through a process of research and development on the resolution of teenagers’ interior struggling, a focal point in order to build a balanced adulthood.

Secondly, we will go through some thoughts on the universal gestures of education (hospitality, care and promotion of his independency) and on the importance of resilience tutors, as adoptive parents or other significant persons that could help the adoptive teenagers in order to “browse the torrents” [2,3]. Finally, it will be briefly illustrated in its main results the most recent and extensive living survey – on European level - on the topic of adolescence and international adoptions ^[4].^ More than 800 families engaged and around 700 teenagers interviewed in order to acknowledge diagnosed pathologies before and after the adoptive process, the relationship with the social context and with the school, the ethnic identity and the difference between “adopted” and “immigrant” teenagers, the psychological healthcare and the relationship with their own origins.

The conclusion will be referred to the centrality of the adopted children and the importance of listening.

**References**

1. Macario G. L’adolescenza nelle adozioni internazionali: complessità e specificità. In: Macario G, editor. I percorsi formativi del 2009 nelle adozioni internazionali. 1st ed. Firenze: Istituto degli Innocenti; 2012. p. 101-180.

2. Cyrulnik B, Malaguti E. Costruire la resilienza. 1st ed. Trento: Erikson; 2005.

3. Cyrulnik B. I compagni, tutori di resilienza. In: Cyrulnik B, editor. Autobiografia di uno spaventapasseri*.* 1st ed. Milano: Raffaello Cortina Editore; 2009. p. 173-176.

4. Bianchi D, Di Gioia G. Adolescenti e adozioni internazionale. 1st ed. Firenze: Istituto degli Innocenti-Carocci Editore; 2016.

## A41 CAM and Vaccinations

### Francesco Macrì, Vitalia Murgia

#### ^1^Pediatrics Department, “Sapienza” University, Rome, Italy; ^2^Pediatrician, Mogliano Veneto (TV), Italy

##### **Correspondence:** Francesco Macrì (profmacri@gmail.com)

**Background**

A complex situation has developed around vaccination practice in Italy in recent years. Stories of the possible harmful effects of vaccines have led to a trend towards abstention and a coverage of less than 95% for some types of vaccination, with the consequent re-emergence of diseases such as measles, which had almost disappeared. This trend has induced health institutions to take defensive measures, such as vaccination prior to registering for school, required in some regions, or sanctions for doctors who are against vaccinations. It is widely believed that the doctors who are most outspoken against vaccinations are those practicing complementary and alternative medicine (CAM), especially homeopathy. Homeopathic prophylaxis of infectious diseases is based on two different methods, homeoprophylaxis and isoprophylaxis (also known as isotherapy), but neither of these has been confirmed as effective [1]. Investigation of these aspects in relation to the attitude of homeopathic doctors towards vaccination reveals some interesting information. Eizayaga and colleagues found, through use of an online survey, that 75.6% of homeopathic doctors believe vaccinations to be useful, effective and safe; this opinion is widespread above all in doctors working in public health and those who do not practice homeopathy as their sole professional specialism [2]. The present study aimed to assess the attitude towards vaccination of paediatricians using CAM.

**Materials and methods**

We distributed the questionnaire used by Eizayaga, partly modified to meet our needs, to a group of 30 paediatricians using CAM and a group of 160 family paediatricians who do not practice CAM. The questions were designed to establish their attitude towards vaccinations.

**Results**

Only 3.4% of CAM paediatricians were totally against vaccination. The remaining 96.6%, while not against it, had some concerns in relation to the priority of the various vaccinations. 23% were in favour of all vaccinations in the Italian national programme, and 50% were in favour of a substantial part of them. No family paediatrician was against vaccination altogether, 75% were in favour of all vaccinations and 23% were in favour of a substantial part of them.

**Conclusions**

Only a small percentage of paediatricians practicing CAM are against vaccinations. The remainder are favourable, indicating a priority in line with the relevance of each infectious disease.

**References**

1. Teixeira MZ. Isoprofilaxis is neither homeoprofilaxis nor homeopathic immunization, but isopathic immunization unsupported by the homeopathic epistemological model: a response to Golden. Int J High Dilution Res 2014; 13:54-82.

2. Eizayaga JE, Waisse S. What do homeopathic doctors think of vaccines? An international online survey. Homeopathy 2016; 105:180-185.

## A42 How to organize a scientific talk and prepare a poster

### Marco Maglione (marcomaglione84@gmail.com)

#### Department of Translational Medical Sciences, Federico II University, Naples, 80131, Italy

Preparing an effective oral presentation may represent a difficult and time-consuming challenge even for the most experienced speaker. One of the main aspects to take into account is that each presentation, even on the same topic, is different and deserves a careful and specific evaluation. First, it is important for a speaker to know his (or her) audience. A talk given to a team of experts in the field will obviously start at a more specialized level than a presentation conceived for a group of students, that will require a more general introductory background. Furthermore, in order to be regarded as someone who is not only competent, but also appealing to the audience, it is crucial to adequately prepare the talk and never give the impression that you don’t handle the topic you are presenting. Eye-contact with the audience and clear speech are among the main qualities of a good speaker, even though not exceeding the time scheduled for a talk, possibly keeping a few minutes for questions, is probably the most important.

Slides should be structured with the aim of capturing audience’s attention on relevant information avoiding unuseful distractions. Nevertheless, varying the visual look of the slides by mixing text, tables, and figures will avoid a bored audience and may turn a simple sequence of data in an attractive story.

Criteria to build a successful poster mostly coincide with those used for an attractive scientific talk, even though a key issue in posters is being concise, using figures instead of words whenever possible. All efforts must be made in order to obtain a poster which is attractive and essential at the same time: a good title will get people to stop, but few clear figures with concise captions will be needed to keep their attention. A synthetic introduction, a clear take-home message and a brief methods section generally represent all the text necessary into a poster.

Both scientific talks and posters are not intended to be second-class solutions to communicate your data, and represent valuable opportunities to present your research. In order to achieve success it is crucial to dedicate time to their preparation, trying to communicate information in a synthetic, attractive and understandable way [1].

Reference

1. Hites RA. How to give a scientific talk, present a poster, and write a research paper or proposal. Environ Sci Technol. 2014; 48:9960-9964.

## A43 Round Table on the humanization of pediatric care in hospital: a questionnaire for users and operators in Campania Region

### Claudia Mandato (claudia.mandato@virgilio.it)

#### Pediatrics, Santobono–Pausilipon Pediatric Hospital, 80100, Naples, Italy

The term "humanization of care" includes a wide range of aspects related to child hospitalization which are still poorly investigated. Analysis of humanization degree has a central role in targeting intervention strategies, for detecting lacking areas in hospital environment and planning effective initiatives. In pediatric setting, data on humanization degree assessment are scarce and based on different and often not comparable methodology. Examples of strategies for analysis of Humanization Degree are present in several countries (Fig. 1).

**Fig. 1:**
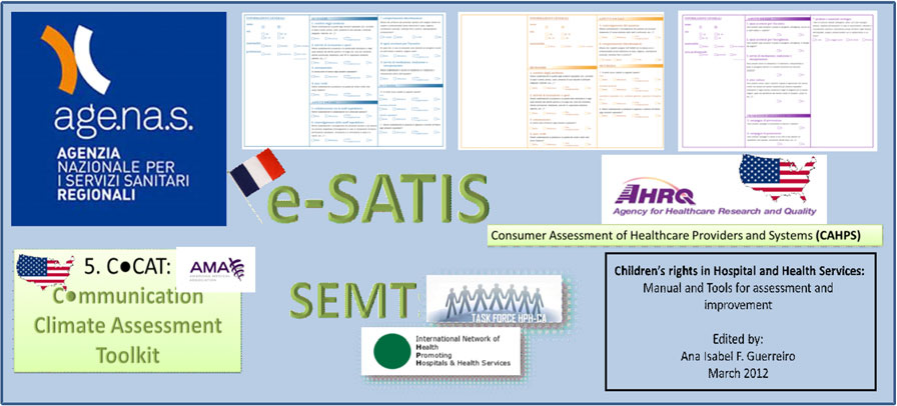
**(abstract A43).** Examples of strategies for analysis of Humanization Degree

In Italy representatives models for assessment and monitoring of Humanization Degree was realized and carried out by AGENAS [1] and University of Milan (LPCP tool), in several general and pediatric hospital settings.

In the context of the project “Analysis and Implementation of the Process of Humanization of the Care Pathways in the Pediatric Hospital Structures of the Campania Region” a novel version of AGENAS survey was realized and administered focusing the items strictly on pediatrics hospital environments. In addition, in order to question the perception of Humanization Degree by families, medical and technical staff the “LPCP tool” questionnaire [2] was also given in the same pediatric departments. The analysis of the respect of children rights in hospital was tested by a specific tool for children and adolescents, in line with task force of International Network of Health Promoting Hospital (HPH). Preliminary data results permit the identification of implementable areas, some of which are common to various pediatric settings including: child-friendly spaces, linguistic mediation, simplifying access to health care, continued care, training course, school continuity plans, patient and family centered care programs. Noteworthy, the existing Humanization Degree is different from those perceived by the operators and the users, and -in the latter- between family and patient himself, with considerable distinction also by age.

**References**

1. Agenas-Agenzia Nazionale per i servizi sanitari Regionali. 2016. Available in http://www.agenas.it. Accessed in June 22, 2017.

2. Buffoli M, Bellini E, Bellagarda A, Di Noia M, Nickolova M, Capolongo S. Listening to people to cure people: theLpCp - tool, an instrument to evaluate hospital humanization. Annali Di Igiene: Medicina Preventiva E Di Comunità. 2014:26; 447–455.

## A44 The experience of Nati per Leggere

### Stefania Manetti (doc.manetti@gmail.com)

#### Family paediatrician, Member of the Nati per Leggere National Coordination Team, Head of the regional association: ACP Campania, Piano di Sorrento, Italy

Nati per Leggere (NpL), website www.natiperleggere.it., is the national Italian program on reading promotion in primary paediatric care. It is implemented, since 1999, by ACP (Associazione Culturale Pediatri), AIB (Associazione Italiana Biblioteche) e CSB (Centro per la salute del Bambino). The aim of NpL is reading aloud promotion in families with children from birth to 5 years of age [1]. Primary care paediatrician, together with other primary care paediatric professionals (nurses) are trained to promote such intervention in their clinical practice, with specific regards to disadvantaged families [2]. Nati per Leggere has in the last 20 years an active collaboration with Reach out and read, website www.reachoutandread.org, a national USA program on reading aloud promotion, considered by the American Academy of Pediatrics (AAP) a program endorsed by the academy and supported by many scientific evidences. AAP has included Reach out and Read together with similar programs, as a best practice that paediatricians should promote in primary care. The Italian experience is described, its similarities with other international programs as Reach out and Read, Bookstart, Stiftung Lesen, and other programs in Spain, Croatia, Switzerland,together with the scientific evidences supporting such intervention in primary care. The role of primary care paediatrician is underlined as influential in promoting early child development (ECD). The precocity of the intervention is remarked as fundamental in order to produce a change in child development trajectory. Nati per Leggere is considered a scientific based intervention which strongly influences well-being, competence in literacy, language and cognitive domains of development, and parenting support. The aim of this discussion is to inform primary care paediatricians and other health care providers on the importance of reading promotion, especially in disadvantaged families. NpL should be included, within the best practices for Early Child Development, also in the training of pediatric residencies [3]. Hospital humanization should include and implement this intervention in order to support parents during hospitalization and to reinforce the information given by other primary health care providers. The early child period is considered the most important phase throughout the lifespan. What happens to the child in the early years is critical for the child’s developmental trajectory and life course.

**References**

1. Tamburlini G. Lettura condivisa in famiglia e sviluppo del cervello nel bambino. Medico e Bambino. 2015; 8:505-510.

2. Manetti S, Panza C, Tamburlini G. Strumenti per i pediatri delle cure primarie. Medico e Bambino. 2011; 30:167-74.

3. National scientific council on the developing child. The science of early child development. Center for the Developing Child, Harvard University; 2007. Available in http://www.developingchild.net. Accessed in July 13, 2017.

## A45 Kawasaki disease

### Alessandra Marchesi, Alberto Villani

#### UOC Pediatria Generale e Malattia Infettive, Ospedale Pediatrico Bambino Gesù, Roma, Italy

##### **Correspondence:** Alessandra Marchesi (alessandra.marchesi@opbg.net)

Kawasaki disease (KD) is an acute, systemic vasculitis [1,2]. According to the “Revised International Chapel Hill Consensus Conference Nomenclature of Vasculitides” of 2012 [3], its target are small and medium diameter vessels in each organ and apparatus. KD is a self-limited disease with unknown, probably multi-factor, aetiology, which primarily affects infants and children under five years. Diagnosis of Kawasaki disease is clinic, based on diagnostic clinical criteria, supported by the results of blood and instrumental exams.

This presentation aims to highlight news in guidelines than those published in 2008. We will evaluate the differences in the definition of different forms, new instrumental techniques (echocardiography, CT Angiography, MRA), updates in therapy both in responder- patients (eg. changing the length and dosage of anti-inflammatory therapy with ASA), and in non-responders, and in major risk patients (adding the steroid therapy). Finally, we will reevaluate the times for follow-up in the different risk classes.

**References**

1. Newburger JW, Takahashi M, Gerber MA, Gewitz MH, Tani LY, Burns JC et al. Diagnosis, treatment, and long-term management in Kawasaki 4disease: a statement for health professionals from the committee on rheumatic fever, endocarditis and Kawasaki disease, council on cardiovascular disease in the young, American Heart Association. Pediatrics 2004; 114:1708-33.

2. Marchesi A, Pongiglione G, Rimini A, Longhi R, Villani A. Malattia di Kawasaki: Linee Guida italiane. Prospettive in Pediatria. 2008; 38:266-83.

3. Jennette JC, Falk RJ, Bacon PA, Basu N, Cid MC, Ferrario F, et al. 2012 Revised International Chapel Hill Consensus Conference Nomenclature of Vasculitides. Arthritis Rheum. 2013; 65:1-11.

## A46 A possible paradox. The disease can turn into opportunity

### Giuseppe Masera (g.masera@asst-monza.it)

#### Clinica Pediatrica Università di Milano-Bicocca, Monza, Italia

Disease-opportunity: in the light of the prevailing guidelines in our culture, juxtaposing the two words has more the meaning of an oxymoron (two opposite concepts) than of a possible paradox. The dominant paradigm in medicine is the bio-medical, centered on disease rather than on the patient with its complexity of needs.

The disease-opportunity concept goes back to some of the great philosophers of ancient Greece (Heraclitus: "is disease that makes health pleasant and good"). Over the centuries, the concept was reiterated by philosophers, poets, scientists: "I learned from disease a lot of what life would never be able to teach" (Goethe).

From the late 1990s the Positive Psychology appears in the scientific landscape, facing the new concept of well-being as full-functioning of the person and not only as absence of illness or negative events. [1].

The research was focused on assessing the subjectivity of the patient, exploring the constructive, creative and propositional aspects of individuals and groups. It was highlighted the phenomenon of RESILIENCE characterized by the ability of some people, although exposed to severe negative events (illnesses, other adverse events), not to show symptoms of distress and disadvantage, but to develop resources previously latent and to achieve positive results in personal and social life [2].

Hoping that this precious contribution of Positive Psychology will be better known by physicians, pediatricians and healthcare professionals, I will describe the professional pathway of over 40 years in the treatment of leukemia and what I have learned about the disease-opportunity relationship. I refer in particular to ALL and to the great progress achieved since the late 1960s. The percentage of successfully treated cases has progressively increased to 70-80%. For many years prevailed the concept that Post Traumatic Stress Disorder should develop after such a serious illness. In the early 2000s, I met Mark Chesler, the author of a fundamental article [3] documenting that cancer is a catalyst for growth in most young-adults successfully treated for cancer or leukemia in their early years. Similar studies are reported in subsequent years and the prevalence of Resilience and Post Traumatic Growth is increasingly documented. We conducted qualitative studies in Monza [4], Nicaragua and other Latin American countries.

Conclusion: yes, the disease can become an opportunity if it is faced with the awareness that after a traumatic event a person is no longer the one it was before: it could face distress, depression and other negative consequences, but more frequently develop growth, allowing to discover new positive values.

**References**

1. Seligman ME, Csikszentmihalyi M. Positive psychology. An introduction. Am Psychol 2000; 55:5-14.

2. Masera G, Delle Fave A. La resilienza: una risorsa da valorizzare. Medico e Bambino 2015; 6:360-364.

3. Parry C, Chesler MA. Thematic evidence of psychosocial thriving in childhood cancer survivors. Qual Health Res 2005; 15:1055-73.

4. Masera G, Jankovic M. Noi ragazzi guariti. Milano; Ancora Editore; 2008.

## A47 Becoming adult with a medical device

### Paola Cianci^1^, Luigi Memo^2^ (luigi.memo@tin.it)

#### ^1^Pediatric Department, University of Insubria, Varese, Italy; ^2^Pediatric Department, S. Martino Hospital, Belluno, Italy

The management of any chronic condition during adolescence, a time of rapid growth and physiological changes accompanied by important individuation and socialization processes, constitutes a challenge for the individual and his family. We would like to discuss the relationship between adolescents’ use of medical devices (e.g. infusion pumps, braces, prostheses, gastrointestinal stomas) and its impact on their quality of life.

Identity, self-image and ego development are affected by chronic diseases in a generic fashion [1], even when the disease leads to the use of devices**.** This is especially true when the illness is more severe and intelligence quotient is higher [2], because of a major sense of awareness. In fact a medical device can be read as sign of diversity that is in contrast with the need to look similar to one’s peers or as a reason of rejection by the group. Because of this, adolescents can easily reject their diagnosis and the correct use of a device, determining the disease worsening.

Body image and the development of a sense of sexuality may also be impaired using devices, which distort the physical body either (e.g. special glasses, a corset, or orthopedic shoes). The need to appear as “normal”, which is especially powerful during adolescence, may lead patients to abandon habits that they had previously accepted without much difficulty. The simplest and most efficient way to investigate these aspects is to ask patients about how well they manage to use the device and how they feel about it.

On the contrary, the choice of involving adolescents in using their device can be a chance to make them more responsible, explaining that having a device does not necessarily mean giving up any experiences (such as adolescents with diabetes, setting their insulin pumps during parties or school trips). If adolescents learn how to control their condition using devices, they progressively get a sense to be able to change positively or control their situation [3,4]. Furthermore, active participation in the negotiation of device use helps young people to get some ownership and control of the disease back from their parents.

Adolescents represent a particularly vulnerable group which should understand their illness, especially the rationale for treatment and treatment options. Information should be provided at a level that is developmentally and cognitively appropriate, to facilitate the acceptance of illness and, consequently, a better quality of life.

References

1. Hauser S, Houlihan J, Powers SI, Jacobson AM, Noam GG, Weiss-Perry B et al. Ego development and self-image complexity in early adolescence. Longitudinal studies of psychiatric and diabetic patients. Arch Gen Psychiatry. 1983; 40:325–332.

2. Silver EJ, Bauman LJ, Coupey SM, Doctors SR, Boeck MA. Ego development and chronic illness in adolescents. J Pers Soc Psychol. 1990; 59:305–310.

3. Hampson SE, Skinner TC, Hart J, Storey L, Gage H, Foxcroft D, et al. Effects of educational and psychosocial interventions for adolescents with diabetes mellitus: a systematic review. Health Technol Assess. 2002; 5:1-79.

4. Greenen S, Powers L, Sells W. Understanding the role of health care providers during the transition of adolescents with disabilities and special health care needs. J Adolesc Health. 2003; 32:225–233.

## A48 Neutropenia: diagnosis and treatment

### Giuseppe Menna (giumenna56@libero.it)

#### Pediatric Haematology Unit, Pausilipon Hospital, Naples, 80123, Italy

Neutropenia is defined as a blood neutrophil count <1500x10^6^/l in children older than 12 months and <1000x10^6^/l in infants between 2 weeks and 1 years of age. In Blacks neutrophils between 200 and 600x10^6^/l are considered normal. Children with neutropenia have an increased susceptibility to bacterial infection, such as pneumonia, otitis, cellulitis and sepsis; stomatitis and gingivitis are also common. These symptoms are more frequent when neutrophils are <500x10^6^/l. Neutropenia can be distinguished in isolated neutropenia (severe congenital neutropenia, cyclic neutropenia, alloimmune and autoimmune neutropenia [1], drug induced neutropenia, idiopathic neutropenia, post viral neutropenia) and neutropenia associated with extrahematopoietic features (Hax1 neutropenia, Shwachman-Diamond disease, Barth Disease, Type 1b glycogenosis, Congenital Diskeratosis, etc). The cumulative incidence of congenital/genetic neutropenia is 1 per million inhabitants; the auto/alloimmune neutropenia is more frequent, about 1 per 100000 inhabitants [2]. The diagnostic work up for neutropenia (Fig 1) [3] is quite complex, and family and personal history is very helpful. In congenital/genetic neutropenias, bone marrow aspirate shows maturation arrest at promyelocytic/myelocytic level; molecular diagnosis can reveal a gene defect in about 75% of congenital neutropenias. The availability of G-CSF since 1993 has dramatically improved the prognosis of the children with severe neutropenia, reducing mortality due severe infectious diseases. Patients responding poorly to G-CSF, or requiring doses greater than 8-10 μg/kg/day, and patients with monosomy 7 have an increased risk of MDS/AML (myelodysplasia/acute myeloid leukemia) evolution. Haematopoietic stem cell transplantation is a therapeutic option in neutropenias that have a greater risk of evolution to MDS/AML, such as severe congenital neutropenia, Shwachman-Diamond disease, Wiskott Aldrich syndrome, Congenital Diskeratosis [4].

**Fig. 1:**
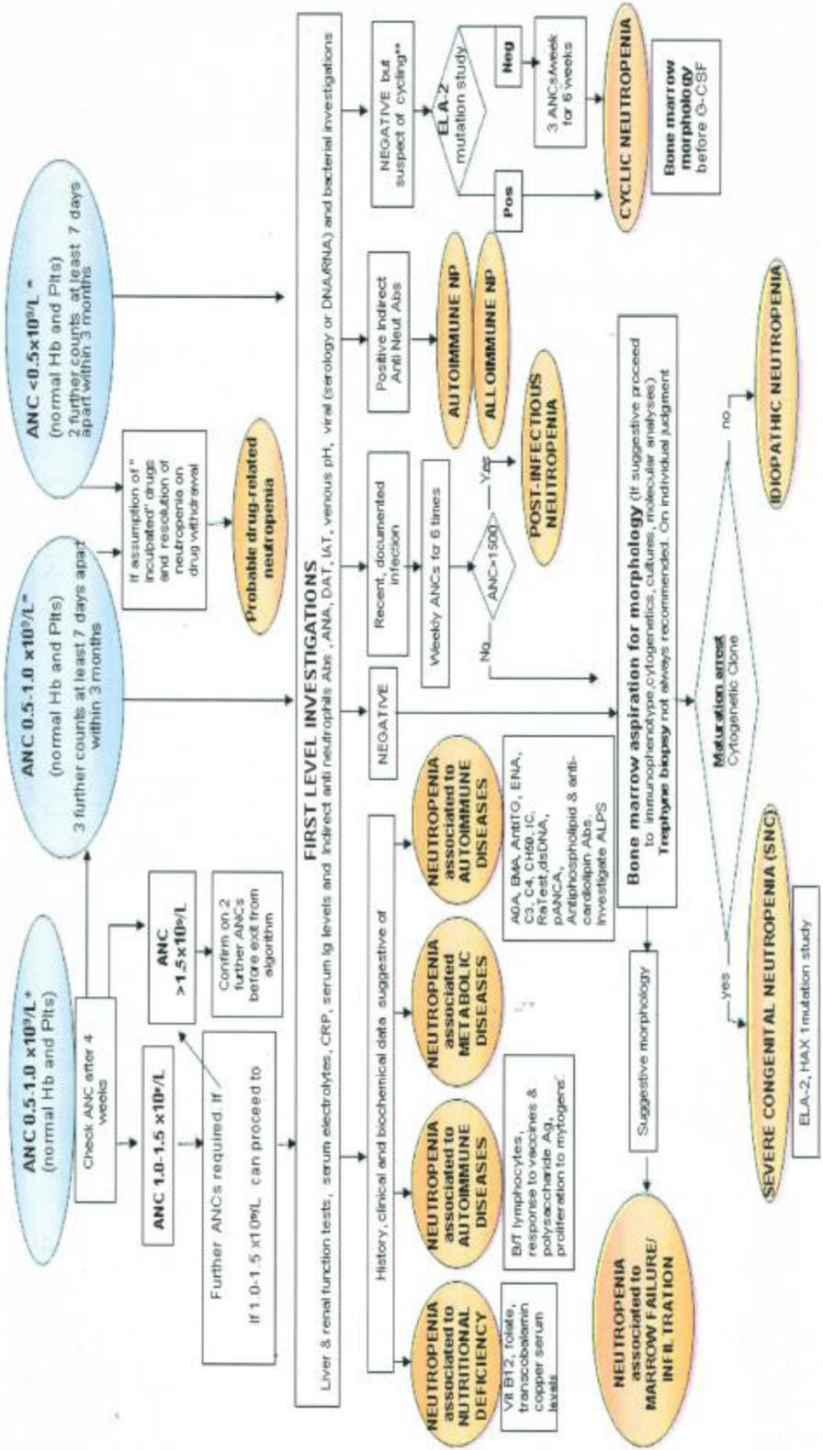
**(abstract A48).** See text for description

**References**

1. Farruggia P, Fioredda F, Puccio G, Porretti L, Lanza T, Ramenghi U, et al. Autoimmune neutropenia of infancy: Data from the Italian neutropenia registry. Am J Hematol. 2015; 90:E221-2.

2. Donadieu J, Beaupain B, Mahlaoui N, Bellanné-Chantelot C. Epidemiology of congenital neutropenia. Hematol Oncol Clin North Am. 2013; 27:1-17.

3. Fioredda F, Calvillo M, Bonanomi S, Coliva T, Tucci F, Farruggia P, et al. Neutropenia Committee of the Marrow Failure Syndrome Group of the AIEOP (Associazione Italiana Emato-Oncologia Pediatrica). Congenital and acquired neutropenias consensus guidelines on therapy and follow-up in childhood from the Neutropenia Committee of the Marrow Failure Syndrome Group of the AIEOP (Associazione Italiana Emato-Oncologia Pediatrica). Am J Hematol. 2012; 87:238-43.

4. Dale DC. How I manage children with neutropenia. Br J Haematol. 2017; 17:1-13.

## A49 Pediatric multiple sclerosis: clinical approach to differential diagnosis

### Milani Nicoletta (nicoletta.milani@istituto-besta.it)

#### Child Neurology Department, Neurological Institute “Carlo Besta”, Milano, Italy

Pediatric multiple sclerosis (MS) is a rare disease, accounting for the 4-10% of all MS patients (with children <10y being the 17% of all pediatric patients), and it is probably not enough considered. A child at his first demyelinating episode is probably more easily diagnosed as having ADE/ADEM (acute demyelinating encephalopathy/encephalomyelopathy) even when diagnostic criteria, mostly presence of encephalopathy, are not fulfilled. Clinical and paraclinical features pointing to MS may help but are not absolute, since an ADEM-like onset occurs in 10% of pediatric MS. Strict clinical and radiological follow-up is recommended. Neuromyelitis optica spectrum disorders (NMOSD) are to be considered when optic neuritis, mostly if bilateral and with poor recovery and/or extense transverse myelitis occur, and/or when peculiar radiological involvement (dorsal brainstem, area postrema, diencephalon, periependymal region of third and fourth ventricle) is seen. Anti-aquaporin 4 antibodies may be detected or not (in the last case adjunctive, more strict radiological and clinical criteria are requested). Tumefactive demyelinating lesions may mimic clinically and radiologically a space occupying lesion and may prompt useless brain biopsy. Open-ring enhancement of the lesion is a distinctive feature. MR spettroscopy and PET-TC may be support the diagnosis. Apart from atypical demyelinating disease, the differential diagnosis of pediatric MS is broad including infections, reumathologic diseases, CNS vasculitis, neurosarcoidosis, macrophage activation syndrome (MAS), mithocondrial diseases et al. An exaustive diagnostic evaluation must therefore be performed [1], to be able to recognize the so-called red flags, which are clinical, imaging or other laboratory features suggesting alternative diagnosis [1, 2, 3, 4]. In details this diagnostic workup should include: a) brain and spine MRI ( with orbital study when visual deficit is present); b) VEP, BAEP, SSEP; c) Screening for infectious diseases ( blood count, ESR,CRP, serology for HSV 1-2, HZV, EBV, Mycoplasma + VDRL, Borrelia,cysticercosis and HTLV tests when clinically or anamnestically supported); d) CSF analysis (including cell count, glucose, lactate, protein, intrathecal IgG synthesis bacterioscopy, colture and CSF PCR pane; e) Screening for NMOSD (Aquaporin-4 and anti-MOG antibodies); f) Screening for MAS ( ferritin and tryglicerides); g) Endocrine screening (fT4, TSH and antiTPO antibodies, when an Hashimoto encephalopathy is Suspected); h) Screening for rheumatologic disease (ANA, anti ds-DNA, ENA, LAC, ACA, anti beta2glycoprotein); i) screening for mithocondrial disease ( serum and CSF pyruvate and lactate); l) screening for nutritional disease (vitamin B12 and vitamin D).

**References**

1. O'Mahony J, Shroff M, Banwell B. Mimics and rare presentations of pediatric demyelination. Neuroimaging Clin N Am. 2013; 23:321-36.

2. Tardieu M, Deiva K. Rare inflammatory diseases of the white matter and mimics of multiple sclerosis and related disorders. Neuropediatrics. 2013; 44:302-8.

3. Tenembaum SN. Pediatric multiple sclerosis: distinguishing clinical and MR imaging features. Neuroimaging. Clin N Am. 2017; 27:229-250

4. Brownlee WJ, Hardy TA, Fazekas F, Miller DH. Diagnosis of multiple sclerosis: progress and challenges. Lancet. 2017; 389:1336-1346.

## A50 The obese adolescent

### Anna Di Sessa, Emanuele Miraglia del Giudice

#### Department of Woman, Child, General and Specialized Surgery, Università degli Studi della Campania "Luigi Vanvitelli", 80138, Naples, Italy

##### **Correspondence:** Emanuele Miraglia del Giudice (emanuele.miragliadelgiudice@unicampania.it)

In the past decades, the prevalence of childhood obesity has strongly increased, by heightening the risk to being obese even during adolescence. Recent data, in fact, indicate that in developed countries a third of the adolescents are overweight or obese. In children and adolescents, overweight and obesity are commonly defined by using the Body Mass Index (BMI, calculated as weight in kilograms divided by height in meters squared), given a BMI value at or above the gender-specific 85^th^ or 95^th^ percentile growth charts for overweight and obesity, respectively. Currently, the WHO (World Health Organization) and IOTF (International Obesity Task Force) systems are the most widely used growth charts in Europe, while the Italian references are addressed to Cacciari et al. on behalf of the Italian Society for Pediatric Endocrinology and Diabetology (ISPED). Considering the seriousness of pediatric obesity epidemic and related comorbidities, recent data suggest the use of WHO references as more sensitive for clinical practice and/or obesity screening [1]. Therefore, since obesity has been linked to an unfavorable both metabolic (Metabolic Syndrome, Insulin-resistance) and cardiac (cardiac remodeling, atherosclerosis) profile, adolescents have an increased cardiometabolic risk. Indeed, it has been now widely recognized a relationship between the BMI within adolescence and consequent cardiovascular mortality in adulthood. Findings from an Israeli-adolescent population-based study have shown that an increased BMI, although within the currently accepted normal range (50-74^th^), is strongly related to cardiovascular and all-cause mortality in young adulthood or midlife [2]. As expected, patients with BMI values above the 84^th^ percentile showed a higher risk of death from stroke, sudden death, and total cardiovascular causes, with an increase more steeply among the extremely obese or cardiovascular-specific death [2]. More importantly, recent data showed that the severity of obesity might identify children and young adults at greater cardiometabolic risk, especially in male gender [3]. Particularly, young patients assigned to class II obesity (≥120% to 140% of the 95 th percentile or BMI ≥35) showed an increased risk for abnormal levels of HDL cholesterol, systolic blood pressure and glucose, conversely subjects assigned to class III obesity (≥140% of the 95 th percentile, or BMI≥40) seem to be at higher risk for hypertriglyceridemia, elevated levels of diastolic blood pressure and glycated hemoglobin [2]. Thus, the persistence of obesity from childhood into the young adulthood may anticipate the onset of serious obesity -related consequences, such as diabetes or hypertension, representing a rising concern of healthcare. Given that, it has been highlighted the key role of obesity prevention and treatment. Diet, physical activity and lifestyle modifications represent the mainstay of treatment of pediatric obesity [4]. However, recent studies evaluating the effectiveness of multidisciplinary weight management programs for obese adolescents have shown moderate results on weight loss as decreased BMI, at least in the short term [5]. Due to conflicting findings from lifestyle programs, weight loss surgery might be considered a valid option for selected young severe obese patient with significant comorbidities who failed medical treatment. To this purpose, recent evidence supports the effectiveness and safety of bariatric surgery, suggesting it as a possible valid option for the treatment of extremely severe obesity in adolescence [6]. A recent multicenter prospective study examined the weight-loss surgery (gastric bypass or sleeve gastrectomy) among adolescents through three follow-up years after the procedure, underscoring its positive effect not only on cardiometabolic health but also in weight-related quality of life in these subjects, although it was reported a specific micronutrient deficiency (e.g. iron and vitamin B 12) and the need for consequent abdominal procedures [6]. In light of this, therefore, an increasingly growing awareness of the future burden of cardiovascular and metabolic diseases is imperative in order to provide an adequate management of adolescent obesity in health care.

**References**

1. Valerio G, Balsamo A, Baroni MG, Brufani C, Forziato C,^7^ Grugni G, et al. Childhood obesity classification systems and cardiometabolic risk factors: a comparison of the Italian, World Health Organization and International Obesity Task Force references. Ital J Pediatr. 2017; 43:19.

2. Twig G, Yaniv G, Levine H, Leiba A, Goldberger N, Derazne E. Body-mass index in 2.3 million adolescents and cardiovascular death in adulthood. N Engl J Med. 2016; 374:2430-40.

3. Skinner AC, Perrin EM, Moss LA, Skelton JA. Cardiometabolic risks and severity of obesity in children and young adults. N Engl J Med. 2015; 373:1307-17.

4. Styne DM, Arslanian SA, Connor EL, Farooqi IS, Murad MH, Silverstein JH et al. Pediatric Obesity-Assessment, Treatment, and Prevention: An Endocrine Society Clinical Practice Guideline. J Clin Endocrinol Metab. 2017; 102:709-757.

5. Al-Khudairy L, Loveman E, Colquitt JL, Mead E, Johnson RE, Fraser H, et al. Diet, physical activity and behavioural interventions for the treatment of overweight or obese adolescents aged 12 to 17 years. Cochrane Database Syst Rev. 2017; 6:CD012691.

6. Inge TH, Courcoulas AP, Jenkins TM, Michalsky MP, Helmrath MA, Brandt ML, et al. Weight loss and health status 3 years after bariatric surgery in adolescents. N Engl J Med. 2016; 374:113-23.

## A51 Bronchiolitis in clinical practice

### Michele Miraglia del Giudice, Amalia Coronella

#### Department of Woman, Child and General and Specialized Surgery, University of Campania “Luigi Vanvitelli”, Naples, Italy

##### **Correspondence:** Michele Miraglia del Giudice (michele.miraglia@unicampania.it)

Acute bronchiolitis is the main cause of respiratory illness requiring hospitalization in children under 2 years of age and the trend in hospitalization rate has been increasing in recent years [1]. Mainly due to Respiratory Syncytial Virus (RSV) infection, the disease leads to hospitalization in only 1% of the children. However, considering that virtually all children before the age of 2 years could be infected, the cost of the disease determined by hospital admissions is elevated. In the United States, it has been shown that the burden of the disease is considerable, having an annual cost of more than $500 million [2]and being responsible for 17% of all infant hospitalizations [2]. Even though immune prophylaxis with monoclonal antibodies (Palivizumab®) has shown promising results in terms of reduction of the prevalence of the disease, this strategy cannot be applied to the whole population because of the high costs [3].

Considering therapeutic options, the mainstays for treatment are supplemental oxygen and hydration [3]. Other types of treatment remain controversial. Corticosteroid denied positive effect in wheezing and hospitalisation rate [3,4]. The role of bronchodilators is debated; bronchiolitis is characterized as predominant pathological features, by airway edema and mucus plugging rather than bronchospam and adrenergic agents used did not assure univocal results [3,5-7].

In bronchiolitis, viral infection causes peribronchial mononuclear infiltration and epithelial cell necrosis, submucosal edema, increased secretion of mucus and a relative dehydration of the airway surface liquid. Nebulized hypertonic saline solution (HS) may substantially contribute to airway rehydratation, reducing edema, enhancing ciliary activity.

The use of HS was demonstrated to be effective to decrease symptoms [8, 9] and length of hospitalization in association to β-adrenergic drugs [9, 10]. In a pre-hospital setting, Al-Ansari et al. compared the effects of 5% vs 3% HS in addition to epinephrine, showing a better response to the more concentrated solution in term of clinical severity scores [11]. More recently, it has been shown that in the treatment of acute bronchiolitis in an emergency department setting, the use of nebulized 3% HS added to epinephrine did not improve clinical outcomes more than normal saline (NS) and epinephrine [12].

In an Italian study, Miraglia del Giudice M et al [13] have shown that, infants hospitalized for bronchiolitis treated with 3% hypertonic saline nebulization presented a more rapid decrease of respiratory symptoms and ameliorated the general condition compared to infants treated with 0.9% saline solution and epinephrine. The effect was already significant after the first 24 hours of therapy and was sustained through the third day of treatment, allowing to discharge the infants treated with 3% HS one day earlier than the NS treated group.

However, therapies that may contribute to the reduction in hospital stay could potentially greatly reduce health costs related to bronchiolitis [2].

References

1. García CG, Bhore R, Soriano-Fallas A, Trost M, Chason R, Ramilo O, et al. Risk factors in children hospitalized with RSV bronchiolitis versus non-RSV bronchiolitis. Pediatrics. 2010; 126:1453-60.

2. Pelletier AJ, Mansbach JM, Camargo CA. Direct medical costs of bronchiolitis hospitalizations in the United States. Pediatrics. 2006; 118:2418-23.

3. American Academy of Pediatrics. Subcommittee on Diagnosis and Management of Bronchiolitis. Pediatrics. 2006; 118:1774-93.

4. Corneli HM, Zorc JJ, Mahajan P, Shaw KN, Holubkov R, Reeves SD, et al. Bronchiolitis Study Group of the Pediatric Emergency Care Applie Research Network (PECARN). A multicenter, randomized, controlled trial of dexamethasone for bronchiolitis. N Engl J Med. 2007; 357:331-9.

5. Gadomski AM, Bhasale AL. Bronchodilators for bronchiolitis. Cochrane Database Syst Rev 2006; (3):CD001266.

6. Wainwright C, Altamirano L, Cheney M, Cheney J, Barber S, Price D, et al. A multicenter, randomized, double-blind, controlled trial of nebulized epinephrine in infants with acute bronchiolitis. N Engl J Med. 2003; 349:27-35.

7. Florin TA, Plint AC, Zorc JJ. Viral bronchiolitis. Lancet. 2017; 389:211-224.

8. Sarrell EM, Tal G, Witzling M, Someck E, Houri S, Cohen HA, et al. Nebulized 3% hypertonic saline solution treatment in ambulatory children with viral bronchiolitis decreases symptoms. Chest. 2002; 122:2015-20.

9. Mandelberg A, Tal G, Witzling M, Someck E, Houri S, Balin A, et al. Nebulized 3% hypertonic saline solution treatment in hospitalized infants with viral bronchiolitis. Chest. 2003; 123:481-7.

10. Baron J, El-Chaar G. Hypertonic saline for the treatment of bronchiolitis in infants and young children: a critical review of the literature. J Pediatr Pharmacol Ther. 2016;21:7-26.

11. Al-Ansari K, Sakran M, Davidson BL, El Sayyed R, Mahjoub H, Ibrahim K. Nebulized 5% or 3% hypertonic or 0.9% saline for treating acute bronchiolitis in infants. J Pediatr. 2010; 157:630-4.

12. Grewal S, Ali S, McConnell DW, Vandermeer B, Klassen TP. A randomized trial of nebulized 3% hypertonic saline with epinephrine in the treatment of acute bronchiolitis in the emergency department. Arch Pediatr Adolesc Med. 2009; 163:1007-12.

13. Miraglia Del Giudice M, Saitta F, Leonardi S, Capasso M, Niglio B, Chinellato I, et al. Effectiveness of nebulized hypertonic saline and epinephrine in hospitalized infants with bronchiolitis. Int J Immunopathol Pharmacol. 2012; 25:485-491.

## A52 Primary immunodeficiencies: from newborn screening to personalized medicine

### Luigi D. Notarangelo (luigi.notarangelo2@nih.gov)

#### Laboratory of Host Defenses, National Institute of Allergy and Infectious Diseases, National Institutes of Health, Bethesda, MD 20892, USA

Severe Combined Immune Deficiency (SCID) is inevitably fatal within the first years of life, unless treated by hematopoietic cell transplantation (HCT). The outcome of HCT for SCID is influenced by several factors: donor-recipient HLA matching, genetic type of SCID, transplant-related toxicity, infections, graft-versus-host disease, and quality of immune reconstitution. A few years ago, Dr. Buckley’s group reported that survival is superior in infants with SCID who receive HCT at <3.5 months of life than in those treated later in life [1]. More recent data from the Primary Immune Deficiency Treatment Consortium have shown that the clinical status of the patient at the time of transplant (lack or presence of active infections) is a better predictor of the outcome of transplant [2]. Overall, these data emphasize the importance of early diagnosis and treatment of SCID. Newborn screening for SCID is widely available in the United States. As a result of its implementation, the number of babies with SCID who go to transplant with active infection has dramatically decreased, and survival after HCT has significantly improved. Characterization of the genetic and molecular pathophysiology of primary immune deficiencies (PIDs) may also open new therapeutic perspectives. For example, a group of patients with Autoimmune Lymphoproliferative Syndrome (ALPS) have been diagnosed to carry CTLA4 haploinsufficiency [3]. In these patients, who develop lymphocytic infiltrates in the grain, lungs, and gut, decreased expression of CTLA4 leads to uncontrolled T cell activation, and in particular increased signaling through the mTOR pathway. Administration of the chimeric molecule CTLA4-Ig, possibly in combination with an mTOR inhibitor (sirolimus) has dramatically improved the clinical status of the patients [4].

Finally, leukocyte adhesion deficiency type 1 (LAD1) is characterized by inability of neutrophils to transmigrate and reach sites of infection/inflammation. These patients suffer from serious infections, cutaneous ulcers, and periodontopathy (leading to loss of teeth). Studies in patients and in animal models have shown that neutrophils that transmigrate to the oral mucosa inhibit macrophage activation by products of the microbial flora. In patients with LAD1, macrophage activation leads to increased release of IL-23 that acts upon Th17 cells favoring production of IL-17 which causes tissue damage. Administration of the monoclonal antibody ustekinumab (which inhibits IL-12/IL-23) leads to dramatic improvement of the periodontopathy and of cutaneous ulcers otherwise resistant to conventional management [5]. These examples are illustrative of what we can expect from advances in the characterization of PID pathophysiology in the near future.

References

1. Myers LA, Patel DD, Puck JM, Buckley RH. Hematopoietic stem cell transplantation for severe combined immunodeficiency in the neonatal period leads to superior thymic output and improved survival. Blood. 2002; 99:872-8.

2. Pai SY, Logan BR, Griffith LM, Buckley RH, Parrott RE, Dvorak CC, et al. Transplantation outcomes for severe combined immunodeficiency, 2000-2009. N Engl J Med. 2014; 371:434-46.

3. Kuehn HS, Ouyang W, Lo B, Deenick EK, Niemela JE, Avery DT, et al. Immune dysregulation in human subjects with heterozygous germline mutations in CTLA4. Science. 2014; 345:1623-7.

4. Lo B, Zhang K, Lu W, Zheng L, Zhang Q, Kanellopoulou C, et al. Autoimmune disease. Patients with LRBA deficiency show CTLA4 loss and immune dysregulation responsive to abatacept therapy. Science. 2015; 349:436-40.

5. Moutsopoulos NM, Zerbe CS, Wild T, Dutzan N, Brenchley L, DiPasquale G, et al. Interleukin-12 and interleukin-23 blockade in leukocyte adhesion deficiency type 1. N Engl J Med. 2017; 376:1141-1146.

## A53 The application of precision medicine in primary immunodeficiencies (PIDs): from “different therapeutic approach for each disease” to “different approach for each patient” Targeting strategies for primary immunodeficiencies (PIDs): models of precision medicine. Toward personalized treatment strategies in primary immunodeficiencies (PIDs)

### Paolo Palma^1^, Paola Zangari^1^, Andrea Finocchi^1,2^, Paolo Rossi^1,2^, Caterina Cancrini^1,2^

#### ^1^Academic Department of Pediatrics, Unit of Immune and Infectious Diseases, Children’s Hospital Bambino Gesù, P.zza Sant’Onofrio, 4-00165, Rome, Italy; ^2^Chair of Pediatrics, Department of Systems Medicine, University of Rome “Tor Vergata”, Rome, Italy

##### **Correspondence:** Paolo Palma (paolo.palma@opbg.net)

Primary immunodeficiencies (PIDs) represent a diagnostic challenge for clinicians [1]. Beyond the classical spectrum phenotype of severe Combined Immunodeficiencies (SCID), atypical clinical manifestations including immune dysregulation often manifesting as autoimmunity, can make diagnosis difficult [2]. The introduction of next generation sequencing methodologies has revolutionized the field of immunogenetics and has led to the recent discovery of a large variety of single-gene abnormalities associated with PIDs [3]. This has allowed moving beyond the classical model of loss of function (LOF) mutation, identifying gain of function (GOF) mutations for the same genes already associated with immunodeficiency, such as those already known in *STAT1* and *STAT3* [4]*.* The exponential discovery of genetic defects in patients with previously undefined PIDs have contributed to improve our understanding of this group of disorders and has opened up the potential for targeted therapy directed at the specific disease-causing abnormality. The final aim is to establish a direct link between PID-specific genetic defects and the associated alteration in cellular signaling pathways in order to tailor targeted therapies. These therapeutic strategies include molecules that either block or enhance cellular pathways, based on the pathogenic mechanism. An interesting model of a targeted therapy focused on the specific GOF mutation is represented by the experimental use of PI3K catalytic subunit p110d in patients with Activated PI3K Delta Syndrome (APDS). In our patient, the use of such molecule reduced the need of immune suppression, thus limiting the administration of IVIG and immunosuppressive drugs. Mechanism-based targeted therapies are currently applied in other selected PIDs such as X-MEN, SAVI syndrome, cytotoxic lymphocyte antigen 4 (CTLA4) haploinsufficiency representing a real opportunity to improve the quality of life and survival of the patients and to reach novel insights of these diseases [5]. In line with this personalized strategies, a multi-disciplinary team of specialists together with the availability of registries and international network are crucial elements that help health care providers to optimize patient-tailored interventions and to select therapies that are more precise, efficient and safe. A similar approach is required in the field of vaccination for immunocompromised children. In this population, validated correlates of protection to identify risk of incomplete or waning immunity are currently lacking and a personalized schedule is required to provide effective and long-term protection [6,7]. Together these approaches, driven by steady progress in the immunopathogenesis, define the basis for treating PIDs in the age of precision medicine (Fig. 1).

**Fig. 1:**
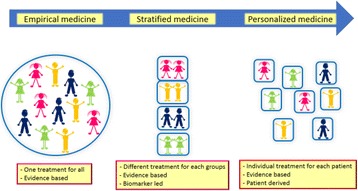
**(abstract A53).** Toward personalized treatment strategies in primary immunodeficiencies (PIDs)

References

1. Picard C, Al-Herz W, Bousfiha A, Casanova JL, Chatila T, Conley ME, et al. Primary immunodeficiency diseases: an update on the classification from the International Union of Immunological Societies Expert Committee for Primary Immunodeficiency 2015. J Clin Immunol. 2015; 35:696-726.

2. Giardino G, Gallo V, Prencipe R, Gaudino G, Romano R, De Cataldis M, et al. Unbalanced Immune System: Immunodeficiencies and Autoimmunity. Front Pediatr. 2016; 4: 107.

3. Gallo V, Dotta L, Giardino G, Cirillo E, Lougaris V, D’Assante R et al. Diagnostics of primary immunodeficiencies through next-generation sequencing. Front Immunol. 2016; 7:466

4. Milner JD, Vogel TP, Forbes L, Ma CA, Stray-Pedersen A, Niemela JE, et al. Early-onset lymphoproliferation and autoimmunity caused by germline STAT3 gain-of-function mutations. Blood. 2015; 125:591–599.

5. Notarangelo LD, Fleisher TA. Targeted strategies directed at the molecular defect: Toward precision medicine for select primary immunodeficiency disorders. J Allergy Clin Immunol.2017; 139:715-23.

6. Cagigi A, Cotugno N, Rinaldi S, Santilli V, Rossi P, Palma P. Downfall of the current antibody correlates of influenza vaccine response in yearly vaccinated subjects: Toward qualitative rather than quantitative assays. Pediatr Allergy Immunol. 2016; 27:22-7.

7. Cagigi A, Cotugno N, Giaquinto C, Nicolosi L, Bernardi S, Rossi P, et al. Immune reconstitution and vaccination outcome in HIV-1 infected children: present knowledge and future directions. Hum Vaccin Immunother. 2012; 8:1784-94.

## A54 Neonatal stabilization before transport

### Roberto Aufieri, Simonetta Picone, Piermichele Paolillo

#### Division of Neonatology and Neonatal Intensive Care, Casilino General Hospital, Roma, Italy

##### **Correspondence:** Piermichele Paolillo (piermpa@tin.it)

Neonates who require care that cannot be provided in the referral center, need to be transferred to the most appropriate location for their clinical conditions. An interfacility transport may bring additional risks to an already critically ill neonate [1]; thus, it should be performed, where available, by a Neonatal Emergency Transport Service (NETS) who has the resources to reduce these risks [2]. Proper patient stabilization before transport is essential to reduce adverse events and to prevent clinical deterioration that may occur during transport [3,4]. Therefore, every center, regardless of its perinatal level, should be able to perform effective resuscitation and stabilization meanwhile awaiting for the transport team.

It has been shown how the lower level of care of the referring hospital, the lower gestational age and the medical reason for transport (compared to surgical) are among factors that increases the stabilization time required by the neonatal transport team at the referral center [5]. Perinatal history, severity and requirements of infant conditions should be appropriately assessed and reported to the transport coordinating center (or transport team), at the time of transfer request, in order to formulate an initial management and transport plan. The transport team will provide consultation and advice to the personnel at the referring center, when required. Stabilization should ensure that physiological parameters are satisfactory: airway, oxygenation, cardiovascular, metabolic and thermal stability should be obtained, maintained and monitored. Special conditions can be encountered and the personnel at the referral center should be trained and equipped to face also with the most challenging scenarios (e.g. pneumothorax, surgical conditions, congenital heart disease, persistent pulmonary hypertension of the newborn, hypoxic ischemic encephalopathy requiring hypothermia). Written consent to transport and other procedures (e.g. hypothermia) should be obtained by parents, who should also should be updated about baby’s clinical conditions and encouraged to reach the receiving hospital as soon as it will be possible. On the arrival of the transport team a structured verbal hand-over should be provided together with the referral letter, results of investigations, images, and the other documentation available. A debriefing among personnel of the referral center should be considered after the management of critical cases. Guidelines for stabilization and resuscitation should be available in every referral centre. Outreach education programs regarding resuscitation and pre-transport stabilization should be periodically performed by the Hub centers or by the transport coordinating centers, also adopting the recent techniques of high-fidelity simulation.

**References**

1. Arora P, Bajaj M, Natarajan G, Auror NP, Kalra VK, Zidane M, et al. Impact of interhospital transport on the physiologic status of very low-birth-weight infants. Am J Perinatol. 2014; 31:237-244.

2. Agostino R, Aufieri R, Gente M. Neonatal transport services. In: Buonocore G, Bracci R, Weindling, M. Neonatology. A practical approach to neonatal diseases. Cham (ZG Switzerland): Springer International Publishing; 2016.

3. Whitfield JM, Buser MK. Transport stabilization times for neonatal and pediatric patients prior to interfacility transfer. Pediatr Emerg Care. 1993; 9:69-71.

4. Goldsmit G, Rabasa C, Rodríguez S, Aguirre Y, Valdés M, Pretz D, et al. Risk factors associated to clinical deterioration during the transport of sick newborn infants. Arch Argent Pediatr. 2012; 110:304-309.

5. Gente M, Di Lallo D, Franco F, Aufieri R, Paolillo P, De Curtis M, et al. Stabilization of the critically ill neonate awaiting transport. Ital J of Pediatr. 2015; 41:A15

## A55 The psychology of the adolescent with chronic disease: what to learn from the diabete

### Ippolita Patrizia Patera (patera@opbg.net)

#### Pediatric Diabetes Center, Bambino Gesù Children Hospital, Roma, 000195, Italy

Adolescence is a time of significant changes. Developmental process among adolescents may include achieving independence from family, formulating values and self-concept, conforming to social norms of peer groups, minimizing differences from one’s peers, forming own identity, are all common developmental processes among adolescents. A chronic illness affects all of these issues. Diabetes is a complex disease that interferes with developing of personal identity and body image, affecting self-esteem, blocking the path towards autonomy, enhancing depressive and opposing features. Diabetes becomes the focus of conflicts [1]. The need to adhere to the habits of the peers and the behavioral patterns imposed, contrasts with good diabetes care practices, generating rebellion and disease negation, with omission of insulin dose, inadequate or at risk behaviors. Family system theory considers adolescent behavior as a function of the dynamic interactions between family members. Diabetes is a Family Condition [2]. Low levels of family conflict and stress, high levels of cohesion and organization, good communication skills, and appropriate involvement of both parents and children in diabetes management, have been associated with higher levels of regimen adherence. Recent studies also suggest that when the fathers are involved in diabetes management, the usual decline in treatment adherence in adolescence is less observed and quality of life is better [3]. The deterioration in glycemic control during adolescence has been widely documented. Females appear to have a higher level of mismanagement of diabetes than males, particularly those in late adolescence, with recurrent admissions for diabetic ketoacidosis. Glycemic failure is often used to lose body weight. Eating disorders such as anorexia, bulimia, binge eating, purging, excessive exercising, and food deprivation occur more often among adolescent girls with diabetes [4]. The Hvidore Study performed in 2,101 adolescents, aged 10-18 years, from 21 centres, who were evaluated through the Diabetes Quality of Life questionnaire, concluded that lower HbA1c was associated with lower impact, fewer worries, greater satisfaction and better health perception in adolescents and showed a direct relationship between general well-being and metabolic control. Diabetes is a risk factor for developing psychological problems in adolescence [5]. The SEARCH Study reported that 14% of youth were mildly depressed and 8.6% were moderately/severely depressed [6]. Diabetes-related characteristics associated with depression in adolescents include poor adherence to treatment and duration of disease. Furthermore, a strong correlation with maternal depression has been described [7].

Complexity of diabetes management in adolescence need a multidisciplinary team trained to support this process.

**References**

1. Weissberg-Benchell J, Nansel T, Holmbeck G, Chen R, Anderson B, Wysocki T, et al. Generic and diabetes-specific parent-child behaviors and quality of life among youth with type1 diabetes. J Pediatr Psychol. 2009; 34:977–988.

2. Minuchin, P. Families and individual development: Provocations from the field of family therapy. Child Development. 1985; 56:289–302.

3. Wysocki T, Gavin L. Paternal involvement in the management of pediatric chronic diseases: Associations with adherence, quality of life, and health status. J Pediatr Psychol. 2006; 31:501–511.

4. Peveler RC, Bryden KS, Neil HAW, Fairburn CG, Mayou RA, Dunger DB, et al. The relationship of disordered eating habits and attitudes to clinical outcomes in young adult females with type 1 diabetes. Diabetes Care. 2005; 28:84–88.

5. Hoey H, Aanstoot HJ, Chiarelli F, Daneman D, Danne T, Dorchy H, et al. Good metabolic control is associated with better quality of life in 2.101 adolescents with type 1 diabetes. Diabetes Care. 2001; 24:1923-1928.

6. Lawrence JM, Standiford DA, Loots B, Klingensmith GJ, Williams DE, Ruggiero A, et al. Prevalence and correlates of depressed mood among youth with diabetes: The SEARCH for Diabetes in Youth Study. Pediatrics. 2006; 117:1348–1358.

7. Whittemore R, Kanner S, Singleton S, Hamrin V, Chiu J, Grey M. Correlates of depressive symptoms in adolescents with type 1 diabetes. Pediatr Diabetes. 2002; 3:135–143.

## A56 Acute post-infectious glomerulonephritis: is it always so benign?

### Carmine Pecoraro, Francesca Nuzzi, Simona Spadarella, Patrizia Di Matteo

#### S.C. di Nefrologia e Dialisi, A.O. Santobono-Pausilipon, Naples, Italy

##### **Correspondence:** Carmine Pecoraro (pecoraro@unina.it)

Acute Post-Infectious GlomeruloNephritis (PIGN) is a common disorder in children, caused by a reactive immunological process that develops following an infection, classically by a streptococcus. Circulating and/or in situ Immune Complexes are deposited and/or formed in the kidney, causing activation of the complement alternative pathway (CAP). The typical clinical presentation is: gross hematuria, mild proteinuria, hypertension, edema and renal function impairment. The laboratory hallmark is the low C3 serum level due to the activation of CAP. Histologically PIGN is characterized by acute essudative proliferation of glomeruli on Light Microscopy (LM) and C3 and Ig deposition on Immunofluorescence (IF**).** C3 Glomerulopathy (C3G), a recently described entity, is caused by dysregulation, of CAP and is characterized by predominant C3 and scanty Ig deposits in glomeruli. It includes C3 Glomerulonephritis, characterized by specific distribution pattern of C3 deposits, and the Dense Deposit Disease characterized by electron-dense deposits in the glomerular basement membrane. The majority of cases of typical acute PIGN, shows recovery within a few days to weeks. In a small percentage of patients, however, the glomerulonephritis takes longer to resolve resulting in persistent hematuria and proteinuria, or even progression to end-stage kidney disease. These patients with ‘atypical’ PIGN may have an underlying defect in the regulation of CAP. These defects include mutations in complement regulating proteins and antibodies to the C3 convertase known as C3 nephritic factors. Hence, the sequela is continuing glomerular deposition of complement factors with resultant inflammation and development of an ‘atypical’PIGN. In these cases it is recommended to perform kidney biopsy for the evaluation on LM, IF and EM and the molecular genetic analysis of C3 and factors regulating its activation. At Santobono children’s hospital in Naples, Unit of Nephrology, we observed, from 2001 to 2016, 194 children (120 M, 74 F; M/F: 1.7, mean age: 5.6 yrs) affected by PIGN. Among these patients 35/194 (18 %) exhibited an atypical PIGN and were submitted to renal biopsy. Sixteen (8%) had a morphological pattern of C3GN. In 12 of them CAP was studied, in 5 we could find mutations in C3 and its regulatory factors. Conclusions: the prognosis of PIGN in children remains globally excellent, but a major attention should be reserved to atypical forms due to the possibility of a worse renal prognosis and, mainly, to the chance to treat them with complement targeted drugs, as the Eculizumab, humanized monoclonal antibody which blocks C5 covertase and so production of sC5b9, membrane attack complex [1].

**Reference**

1. Ghaithi B, Chanchlani R, Riedl M, Thorner P, Licht C. C3 Glomerulopathy and post-infectious glomerulonephritis define a disease spectrum. Pediatr Nephrol. 2016; 31:2079-86.

## A57 Systematic integrated national program for the management of asplenia in Italy

### Silverio Perrotta, Maddalena Casale

#### Dipartimento della Donna, del Bambino e di Chirurgia Generale e Specialistica, Università degli Studi della Campania “Luigi Vanvitelli”, Naples, Italy

##### **Correspondence:** Silverio Perrotta (silverio.perrotta@unicampania.it)

**Background**

Asplenia is a condition due to spleen absence or dysfunction, which leads to high risk of infections and thrombotic events with significant chance of mortality and morbidity. A number of national and international recommended interventions in asplenic patients [1,2] has been published but adherence to recommendations was shown to be poor [3]. The cost to the health system of asplenia- related complications can be significant, and systematic approaches were demonstrated to be cost-effective [4]. In Italy no common policy of patient care has yet been developed, and management of asplenia is mainly case or locally directed. Our aim was to investigate the feasibility of a national program for the management of asplenia and to create a national working group focused on asplenic patients.

**Materials and methods**

Centers of the Italian Association of Pediatric Hematology Oncology (AIEOP) and the Italian Society of Thalassemia and Hemoglobinopathies (SITE) were invited to participate in the Italian Network on Asplenia. The coordinating centre sent a registration form to all doctors who formally agreed to the proposal.

**Results**

Thirty- six care centers formally agreed to the project. Asplenic patients were registered in an electronic database in order to conduct a national census of asplenic patients. Data about reason and duration of asplenia, type of surgery and post-surgery complications for splenectomized patients, long term infectious and thrombotic complications, antibiotic and vaccine prophylaxis and causes of death were entered in an electronic case report forms. At the last update, the database included data from 1312 patients. Meetings of involved parties were held to discuss key points in the management of asplenic patients and to develop a consensus on recommendations for patient care. A national working group of experts developed an algorithm to instruct in the fast and appropriate management of infections in asplenic children.

**Conclusions**

This is the first systematic approach to the management of asplenia in Italy and the most recent program in Europe, as previous reports are long-standing [4]. Comprehensive national project is feasible in Italy. Till now the main concern for asplenic patients has been the high risk of overwhelming infections, but there is a growing body of knowledge on thrombotic risk in asplenic patients. The novelty of this project consists in collecting data regarding this issue, which has been neglected so far. Finally, this project has the potential for implementing research and public health purposes in this target population at a national level.

**References**

1. Kanhutu K, Jones P, Cheng AC, Grannell L, Best E, Spelman D. Spleen Australia guidelines for the prevention of sepsis in patients with asplenia and hyposplenism in Australia and New Zealand. Intern Med J. 2016. doi: 10.1111/imj.13348. [Epub ahead of print].

2. Davies JM, Lewis MP, Wimperis J, Rafi I, Ladhani S, Bolton-Maggs PH; British Committee for Standards in Haematology. Review of guidelines for the prevention and treatment of infection in patients with an absent or dysfunctional spleen: prepared on behalf of the British Committee for Standards in Haematology by a working party of the Haemato-Oncology task force. Br J Haematol. 2011; 155:308-17.

3. El-Alfy MS, El-Sayed MH. Overwhelming postsplenectomy infection: is quality of patient knowledge enough for prevention? Hematol J. 2004; 5:77-80.

4. Woolley I, Jones P, Spelman D, Gold L. Cost-effectiveness of a post-splenectomy registry for prevention of sepsis in the asplenic. Aust N Z J Public Health. 2006; 30:558-61.

## A58 New treatments for asthma in children

### Laura Tenero, Giorgio Piacentini

#### Pediatric Dpt. University of Verona**,** Verona, Italy

##### **Correspondence:** Giorgio Piacentini (giorgio.piacentini@univr.it)

Most asthmatic patients can be controlled by stepwise approach described in current available guidelines with inhaled steroids, LABA and LTRA. However, new treatments are currently under evaluation to improve asthma control. In particular, interventions on the immune-pathogenesis of the disease, such as T-helper type 2 (Th2) inflammation, mediated by interleukin (IL)-4, IL-5, IL-9 and IL-13, which are involved in the pathway of allergic asthma [1], are actively evaluated.

Several different treatments have been proposed to prevent T-cell activation, to modulate Th1/Th2 differentiation, inhibit Th2 related cytokines and inhibit the mediators involved in the disease [2].

In children, the inhibition of the downstream mediators through the use of anti-IgE has been shown to represent a promising approach for the treatment of severe asthma [3].

Omalizumab, a monoclonal human antibody which can antagonize the role of IgE in the pathogenesis of the allergic asthma, is a currently available clinical option for the treatment of children with severe asthma [4]. A number of other antibodies have been evaluated in asthma, including anti-IL-5 (Mepolizumab), anti-IL-4 (Pascolizumab), anti-IL-13 (Lebrikizumab). Interleukin 5 (IL-5), in particular play a pivotal role in eosinophil activation and airway hyperresponsiveness [5]. It can act on the migration of eosinophils to the sites of inflammation as well as in promoting the survival of eosinophils by preventing apoptosis. A number of studies suggest a rationale for the modulation of eosinophils inflammation in asthma showing a potential emerging strategy with anti-IL-5 antibody (Mepolizumab) in the treatment of asthma [6]. Dupilumab is a recently developed monoclonal antibody targeting IL-4/IL-13 which has been evaluated in the treatment of asthma in adults and is under evaluation in children.

Further potential targets for new anti-asthma treatments targeting airway inflammation may include anti-TNFα, anti-CCR3, anti-CCR4 and anti-OX40L, but they still are under pre-clinical evaluation.

New strategies to improve asthma control have been proposed in the recent years and are currently under development and evaluation to the purpose of achieving a tailored approach to asthma treatment in children.

**References**

1. Carr TF, Peters AT. Asthma: principles and treatment. Allergy and Asthma Proc. 2012; 33:39-43.

2. Pahl A, Szelenyi I. Asthma therapy in the new millennium. Inflamm Res 2002; 51:273-282.

3. Chang TW. The pharmacological basis of anti-IgE therapy. Nat Biotechnol. 2000; 18:157-162.

4. Soler M, Matz J, Townley R, Buhl R, O'Brien J, Fox H, et al. The anti-IgE antibody omalizumab reduces exacerbations and steroid requirement in allergic asthmatics. Eur Respir J. 2001; 18:254-261.

5. Shi HZ, Xiao CQ, Zhong D, Qin SM, Liu Y, Liang GR, et al. Effect of inhaled interleukin-5 on airway hyperreactivity and eosinophilia in asthmatics. Am J Respir Crit Care Med. 1998; 157:204-9.

6. Catley MC, Coote J, Tomlinson KL. Monoclonal antibodies for the treatment of asthma Pharmacol Ther. 2011; 132:333–351.

## A59 Adenopathies in Adolescence

### Davide Massano, Elisa Carraro, Marta Pillon

#### Clinic of Hematology-Oncology, University-Hospital, Padova, 35128, Italy

##### **Correspondence:** Marta Pillon (marta.pillon@unipd.it)

**Background**

The management of lymphadenopathy (LAP) without any associated symptom in the adolescence is a hard challenge for pediatrician because a differential diagnosis between the more frequent non malignant disease and neoplasm has to be established [1, 2]. In fact, Hodgkin lymphoma is the first cause of death in adolescent patients [3], and usually the first symptoms are a chronic and generalized adenopathy with/without associated symptoms [4]. The peak incidence of lymphomas is about 9/100.000 between 15-19 years [5].

**Materials and methods**

We conducted a systematic review of the literature on the LAP in adolescence in order to evaluate the etiologies, and the clinical and laboratory signs associated to malignancies. We performed a research on Pub Med with the following terms: ("lymphadenopathy"[MeSH Terms] OR "lymphadenopathy"[All Fields]) AND "adolescent"[MeSH Terms]. A wider research was done with: ("lymphadenopathy"[MeSH Terms] OR "lymphadenopathy"[All Fields]) AND ("infant"[MeSH Terms] OR "child"[MeSH Terms] OR "adolescent"[MeSH Terms]). We used the language limit English.

**Results**

The first literature research reported 2,512 papers (20 systematic reviews, 2 practice guidelines, 5 meta-analysis, 128 non-systematic reviews), but no one focused on the clinical management of the lymphadenopathy in adolescence. The second research reported 3,969 papers (31 systematic reviews, 5 practice guidelines, 5 meta-analysis, 315 non-systematic reviews). One systematic review [6] underlines clinical features which may recognize a malignancy according to NICE guideline recommendation [4]. The main etiologies of chronic LAP in childhood are reactive LAP, lymphoid hyperplasia, tuberculosis, chronic lymphadenitis and primitive tumor as Hodgkin lymphoma, non-Hodgkin lymphoma or metastasis of nasopharynx carcinoma, thyroid carcinoma or rhabdomyosarcoma [7]. More rare are Kikuchi disease (mean age at diagnosis 12.9 years) [8] or autoimmune lymph proliferative syndrome (mean age at diagnosis 15 years) [9]. Some suspicious symptoms that could be related to malignancies: non-tender lymph nodes, firm or hard, diameter >3 cm, localization supraclavicular, axillary or inguinal in the absence of skin changes, persistent (>6 weeks) or generalized form (>2 non-contiguous sites); systemic signs as persistent fever, itching, night swat, weight loss, fatigue, petechiae or other hemorrhagic lesions; persistent hepatosplenomegaly; symptoms related to mediastinal and/or abdominal masses [6]. Moreover, the presence of lymph nodes with suspected pathological signs, associated with the increase of lactate-dehydrogenase levels (>600 IU/l), must be investigated by imaging techniques [1].

**Conclusions**

This systematic review lacks to identify any particular papers focus on the LAP in adolescence. The management of LAP should follow pediatric guideline keeping in mind all differential diagnosis.

**References**

1. Yaris N, Cakir M, Sözen E, Cobanoglu U. Analysis of children with peripheral lymphadenopathy. Clin Pediatr (Phila). 2006; 45:544-9.

2. Citak EC, Koku N, Demirci M, Tanyeri B, Deniz H. A retrospective chart review of evaluation of the cervical lymphadenopathies in children. Auris Nasus Larynx. 2011; 38:618-21.

3. Sender L, Zabokrtsky KB. Adolescent and young adult patients with cancer: a milieu of unique features. Nat Rev Clin Oncol. 2015; 12:465-80.

4. NICE. Suspected cancer: recognition and referral. 2015. Available in https://www.nice.org.uk/guidance/ng12. Accessed in July 7 2017.

5. Group AW, CCM, Group AW. Italian cancer figures, report 2012: Cancer in children and adolescents. Epidemiol Prev. 2013; 37:1-225.

6. Chiappini E, Camaioni A, Benazzo M, Biondi A, Bottero S, De Masi S, et al. Development of an algorithm for the management of cervical lymphadenopathy in children: consensus of the Italian Society of Preventive and Social Pediatrics, jointly with the Italian Society of Pediatric Infectious Diseases and the Italian Society of Pediatric Otorhinolaryngology. Expert Rev Anti Infect Ther. 2015; 13:1557-67.

7. Oguz A, Karadeniz C, Temel EA, Citak EC, Okur FV. Evaluation of peripheral lymphadenopathy in children. Pediatr Hematol Oncol. 2006; 23:549-61.

8. Kim JY, Lee H, Yun B. Ultrasonographic findings of Kikuchi cervical lymphadenopathy in children. Ultrasonography. 2017; 36:66-70.

9. Price S, Shaw PA, Seitz A, Joshi G, Davis J, Niemela JE, et al. Natural history of autoimmune lymphoproliferative syndrome associated with FAS gene mutations. Blood. 2014; 123:1989-99.

## A60 What is pediatric multiple sclerosis? What is special, what is new, what do we really need to know?

### Daniela Pohl (dpohl@cheo.on.ca)

#### Associate Professor and Pediatric Neurologist, Faculty of Medicine, Department of Pediatrics University of Ottawa, Canada

Over the past two decades, pediatric multiple sclerosis (MS) has emerged from a denied and ignored disease to a well-recognized disorder, moving into the focus of international research efforts, but also attracting growing public attention. There has been an exponential increase in publications, with more than 300 peer-reviewed articles on pediatric MS over the past 10 years. With rising diagnostic awareness of pediatric MS, the paradigm of early treatment emerged as a consensus standard of care. However, early diagnosis and treatment of pediatric MS is being complicated by the fact that there is no single disease-defining diagnostic test, that there are more MS mimics in childhood as compared to adult-onset MS, and that the results of the first pediatric MS therapeutic drug trials are still pending.

This presentation will aim to summarize the most up-to-date, clinically relevant knowledge on pediatric MS, including epidemiology, pathophysiology, etiology, genetic and environmental risk factors, diagnostic criteria, biomarkers, prognostic factors and outcome. The other speakers in this symposium will additionally cover the crucial topics of differential diagnosis, therapeutic strategies, and Italian MS projects and networks.

Pediatric MS and other inflammatory CNS demyelinating diseases constitute a group of disorders with potentially devastating irreversible neurological consequences. I hope that this symposium will facilitate the best possible care for those young patients, by providing the knowledge basis and expert opinions required for informed decisions with regards to diagnostic and treatment strategies. Future research will hopefully elucidate preventative approaches, and provide more reliable information with regards to treatment risks and responses. In view of the relatively low incidence of pediatric demyelinating disorders, a collaborative, international approach is required.

## A61 Hyperglycemia in the adolescent: from the pathogenesis to the therapy

### Barbara Predieri, Lorenzo Iughetti

#### Department of Medical and Surgical Sciences of the Mother, Children and Adults, University of Modena and Reggio Emilia, Modena, 41124, Italy

##### **Correspondence:** Barbara Predieri (barbara.predieri@unimore.it)

The term diabetes mellitus describes a complex metabolic disorder characterized by chronic hyperglycemia resulting from defects in insulin secretion, insulin action, or both. Diabetes usually presents with symptoms such as polyuria, polydipsia, nocturia/enuresis, and weight loss. In its most severe form, ketoacidosis may develop. If symptoms are present, urinary ‘dipstick’ testing for glycosuria and ketonuria, or measurement of glucose and ketones using a glucometer, must be performed [1-2]. The etiology of diabetes is heterogeneous but most cases can be classified into two etiopathogenetic categories: type 1 diabetes (T1D; absolute deficiency of insulin secretion); type 2 diabetes (T2D; combination of resistance to insulin action and an inadequate compensatory insulin secretory response) [2]. T1D is characterized by chronic autoimmune destruction of pancreatic β-cells by CD4+ and CD8+ T-cells and macrophages infiltrating the islets. The etiology is multifactorial and the specific roles for genetic susceptibility, environmental factors, the immune system, and β-cells in the pathogenic processes underlying T1D remain unclear. All patients with T1D will require insulin therapy that must be started as soon as possible after diagnosis. Whatever insulin regimen is chosen, it must be supported by comprehensive education [2]. T2D is highly associated with a family history of diabetes, obesity and lack of exercise and most affected individuals exhibit visceral obesity. The two main pathological defects are impaired insulin secretion through a dysfunction of the pancreatic β-cell, and impaired insulin action through insulin resistance [1]. Unlike T1D, there is no identified autoimmune process leading to inadequate insulin secretion in T2D and inadequate insulin secretion appears to result from genetic, environmental, and metabolic causes that may differ between individuals [3]. Lifestyle change should be initiated at the time of diagnosis. Initial T2D treatment of youth is determined by symptoms, severity of hyperglycemia, and presence or absence of ketosis/ketoacidosis. Pharmacologic therapy should include metformin and insulin alone or in combination [4]. Finally, the maturity-onset diabetes of the young (MODY) is a familial form of mild and non-ketotic hyperglycemia diagnosed before twenty-five years. It results from dominantly acting heterozygous mutations in genes involved in the development or function of pancreatic β-cells. The early molecular diagnosis helps predict the expected clinical course and guide the most appropriate management, including pharmacological treatment [5]. In conclusion, there is considerable overlap in the presentation of T1D, T2D, and monogenic diabetes. The early differentiation between different forms of diabetes has important implications for both treatment and education.

**References**

1. American Diabetes Association. Classification and diagnosis of diabetes. Diabetes Care. 2015; 38: 8-16.

2. Craig ME, Jefferies C, Dabelea D, Balde N, Seth A, Donaghue KC. Definition, epidemiology, and classification of diabetes in children and adolescents. Pediatr Diabetes. 2014; 15:4–17.

3. Druet C, Tubiana-Rufi N, Chevenne D, Rigal O, Polak M, Levy-Marchal C. Characterization of insulin secretion and resistance in type 2 diabetes of adolescents. J Clin Endocrinol Metab. 2006; 91: 401–404.

4. Zeitler P, Fu J, Tandon N, Nadeau K, Urakami T, Bartlett T, Maahs D. Type 2 diabetes in the child and adolescent. Pediatr Diabetes. 2014; 15:26–46.

5. Rubio-Cabezas O, Hattersley AT, Njølstad PR, Mlynarski W, Ellard S,White N, et al. ISPAD Clinical Practice Consensus Guidelines 2014. The diagnosis and management of monogenic diabetes in children and adolescents. Pediatr Diabetes. 2014; 15:47-64.

## A62 Diabetes ketoacidosis management in adolescents with type 1 diabetes: lights and shadows

### Ivana Rabbone (ivana.rabbone@unito.it)

#### Pediatric Diabetes Centre, Department of Pediatric, Regina Margherita Children Hospital, Turin, Italy

Diabetic ketoacidosis (DKA) is an acute emergency that occurs both in newly diagnosed patients and in those with known diabetes. In a recent longitudinal population-based study the frequency of DKA at the onset of type 1 diabetes in Italian children under 15 years of age, during 2004–2013, was 40.3% (95% CI: 39.3–41.4%), with 29.1% and 11.2% for mild/moderate and severe DKA, respectively. Severe DKA increased significantly during the period; younger-age children and children living in Southern Italy compared to Central Italy were at significantly higher risk of DKA and severe DKA [1]. The incidence of DKA in Italian children and adolescents with known diabetes is 2.4 events/100 patient-years and adolescence in female gender is one of the higher risk factor [2-3].

The primary goals of DKA therapy are to correct dehydration and electrolyte depletion and reverse ketoacidosis. It should be noted that all used guidelines are based on limited high-quality scientific evidence and much of the content is based on expert consensus. Nevertheless, it is important to have written recommendations to improve DKA management and increase the effectiveness and safety of clinical practice.

In Italy, pediatric diabetologists belonging the Diabetes Study Group of Italian Society of Endocrinology and Diabetology (ISPED) sought to write and implement recommendations for DKA management from an evidence-based pathway taking into account the last 2014 ISPAD consensus guidelines and subsequent critical review articles in an attempt to reduce the considerable variability in management among pediatric centers and improve overall treatment of pediatric DKA [4]. Key points of Italian DKA management are summarized in Table 1.

**Table 1:** **(abstract A62).** Key points of Italian DKA management

Begin with an isotonic solution (0.9 % saline) at 5–10 ml/kg/h over 90–120 min (not exceeding 300 ml/h); do not use colloids
At the beginning of hydration if hypokalemic, but at the latest from the start of insulin therapy, add potassium (20 mmol/L before or 40 mmol/L from the start of insulin infusion*)* as 50 % potassium chloride and 50 % potassium phosphate
Start IV insulin infusion as human regular insulin not before 90–120 min and never give an insulin bolus. It is recommended to utilize an automated syringe for insulin delivery
The recommended insulin dosage is 0.05–0.1 U/kg/h according to patient’s age, but less insulin (0.025–0.05/kg/h) is better and safer
Continue from the third hour with 0.9 % saline
The rate of IV fluid should be calculated to rehydrate evenly over at least 48 h; be careful not to exceed 1.5 times the daily maintenance
When the blood glucose level drops to 250–300 mg/dl (14–17 mmol/l), or decreases faster than 100 mg/dl (6 mmol/l))/h, add glucose 5–10 %, but the fluid replacement should continue to have a tonicity equal to or greater than 0.45 % saline
The use of bicarbonate is not recommended

The goal of guidelines for DKA management is to improve the safety and effectiveness of patient care that should not be dependent on the patient’s location in a country or region. Practice variability and patient safety issues in DKA management prompted development and improvement in the Italian DKA clinical standard work, through the use of well-written and shared guidelines among all healthcare providers.

**References**

1. Cherubini V, Skrami E, Ferrito L, Zucchini S, Scaramuzza A, Bonfanti et al. High frequency of diabetic ketoacidosis at diagnosis of type 1 diabetes in Italian children: a nationwide longitudinal study, 2004-2013. Sci Rep. 2016; 6:38844.

2. Cherubini V, Pintaudi B, Rossi MC, Lucisano G, Pellegrini F, Chiumello G et al. Severe hypoglycemia and ketoacidosis over one year in Italian pediatric population with type 1 diabetes mellitus: a multicenter retrospective observational study. Nutr Metab Cardiovasc Dis. 2014; 24:538-46.

3. Wolfsdorf, J, Glaser N, Sperling MA. Diabetic Ketoacidosis in Infants, Children, and Adolescents. A consensus statement from the American Diabetes Association. Diabetes Care. 2006; 29:1150-59.

4. Rabbone I, Bonfanti R, Cardella F, Buono P, Cauvin V, Cherubini V et al. Raccomandazioni per la gestione della chetoacidosi diabetica in età pediatrica - Gruppo di Studio di Diabetologia Pediatrica S.I.E.D.P. Acta Biomed. 2015; 86:4–25.

## A63 Hospital care in the paediatric population: network between paediatrician, nurse and volunteers

### Valeria Raia (raia@unina.it)

#### Department of Translational Medical Sciences, Regional Cystic Fibrosis Centre, University Federico II, Naples, Italy

Cystic fibrosis (CF) is a severe genetic illness associated with high healthcare utilization and healthcare costs, even when compared to other chronic illnesses. Patients with CF are obligated to correcty take several medications for improving quality of life and survival. Age, gender and lung function may impact on medical adherence. Several studies showed that lower adherence and poorer medical outcomes are strongly associated with greater healthcare utilization, highlighting the importance of addressing the mental health needs of chronically ill patients. In this context non-physician providers including nurse practitioners are very important members of CF care teams that may improve adherence when care processes are shared in a continous standardized survey of CF programs. Setting of national organizations, involving families and friends of individuals with CF and other volunteers, has improved knowledge about CF among public health authorities, and general public and increased collaboration between groups and organizations (including pharmaceutical companies) at the national, regional, and international levels.

## A64 Antipsychotic medication in children and adolescents

### Renata Rizzo, Mariangela Gulisano

#### Child and Adolescent Neuropsychiatry, Department of Clinical and Experimental Medicine, Catania University, Catania, Italy

##### **Correspondence:** Renata Rizzo (rerizzo@unict.it)

Antipsychotics (APs) based on their mechanisms and effects, can be categorized into typical (first generation) and atypical (second generation) APs [1] ; APs are frequently prescribed in the treatment of children and adolescents psychopathologies such as behavioural disorder, autism spectrum disorder, tic spectrum disorder, bipolar disorder, schizophrenia etc. Several adverse events (eg: metabolic, cardiovascular and neurological) are associated with the administration of APs.

In recent years, APs have increasingly used in pediatric population, despite the fact that many of these drugs were approved based on clinical trials in adult patients only and that children are at higher risk for some adverse events like extrapyramidal and metabolic side effects than adults [2]. It has been recently reported that pediatric patients presented a statistically significant increase of adverse events, compared to adult population [4]. Studies of pediatric outpatients have revealed several deficiencies in monitoring practices for adverse effects associated with APs. The majority of the young patients treated with APs show at least one adverse event, even if not serious [3].

Second-generation APs, especially risperidone and aripiprazole, are the most frequently used in children, with a good efficacy [5]. They could be associated, in genetic predispose patients, with weight gain [6], dyslipidemia [7], insulin resistance [8], blood pressure increases and QT prolongation [9,10,11] and extrapyramidal side effects [12]. When choosing an antipsychotic treatment, patients and their families should be included in a careful risk-benefit assessment. Consideration of adverse effects, as well as dietary and lifestyle counseling, should be part of any antipsychotic treatment initiation and continuation. Routine, proactive monitoring of side effects is essential to optimize patient outcomes. In all treatment decisions, the benefits of improving often severe and debilitating symptomatology must be balanced against the varying risks of adverse effects associated with specific antipsychotic agents in child and adolescent patients [13]. Long term pharmacogenomics researches are suggested in order to identify gene signatures that can be used to predict children at risk of adverse events.

**References**

1. Preston JD, O’Neil JH, Talalaga MC. Handbook of clinical psychopharmacology for therapists. New Harbinger Publications, Oakland. 2013

2. Vitiello B, Correll C, van Zwieten-Boot B, Zuddas A, Parellada M, Arango C. Antipsychotics in children and adolescents: increasing use, evidence for efficacy and safety concerns. Eur Neuropsychopharmacol. 2009; 19: 629-635.

3. Rafaniello C, Pozzi M, Pisano S, Ferrajolo C, Bertella S, Sportiello L et al. Second generation antipsychotics in 'real-life' paediatric patients. Adverse drug reactions and clinical outcomes of drug switch. Expert Opin Drug Saf. 2016; 15:1-8.

4. Sagreiya H, Chen YR, Kumarasamy NA, Ponnusamy K, Chen D, Das AK. Differences in Antipsychotic-Related Adverse Events in Adult, Pediatric, and Geriatric Populations. Cureus. 2017; 26:9

5. Pozzi M, Pisano S, Bertella S, Capuano A, Rizzo R, Antoniazzi S et al. Persistence in Therapy With Risperidone and Aripiprazole in Pediatric Outpatients: A 2-Year Naturalistic Comparison. J Clin Psychiatry. 2016; 77: 1601-1609.

6. Yoon Y, Wink LK, Pedapati EV, Horn PS, Erickson CA. Weight Gain Effects of Second-Generation Antipsychotic Treatment in Autism Spectrum Disorder. J Child Adolesc Psychopharmacol. 2016; 26:822-827.

7. Rizzo R, Eddy CM, Calí P, Gulisano M, Cavanna AE. Metabolic effects of aripiprazole and pimozide in children with Tourette syndrome. Pediatr Neurol. 2012; 47:419-422.

8. Rojo LE, Gaspar PA, Silva H, Risco L, Arena P, Cubillos-Robles K et al. Metabolic syndrome and obesity among users of second generation antipsychotics: A global challenge for modern psychopharmacology. Pharmacol Res. 2015; 101:74-85.

9. Rizzo R, Gulisano M, Calì PV, Di Pino A. Mandatory electrocardiographic monitoring in young patients treated with psychoactive drugs. Eur Child Adolesc Psychiatry. 2013; 22:577-579.

10. Gulisano M, Calì PV, Cavanna AE, Eddy C, Rickards H, Rizzo R. Cardiovascular safety of aripiprazole and pimozide in young patients with Tourette syndrome. Neurol Sci. 2011; 32:1213-1217.

11. Palanca-Maresca I, Ruiz-Antorán B, Centeno-Soto GA, Forti-Buratti MA, Siles A, Usano A et al. Prevalence and Risk Factors of Prolonged Corrected QT Interval Among Children and Adolescents Treated With Antipsychotic Medications: A Long-Term Follow-Up in a Real-World Population. J Clin Psychopharmacol. 2017; 37:78-83.

12. Rizzo R, Gulisano M, Calì PV. Oculogyric crisis: a rare extrapyramidal side effect in the treatment of Tourette syndrome. Eur Child Adolesc Psychiatry. 2012; 21:591-592.

13. Correll CU, Kratochvil CJ, March JS. Developments in pediatric psychopharmacology: focus on stimulants, antidepressants, and antipsychotics. J Clin Psychiatry. 2011; 72:655-70.

## A65 Functional gastrointestinal disorders in children

### Valeria Dipasquale, Maria Ausilia Catena, Claudio Romano

#### Department of Human Pathology in Adulthood and Childhood “G.Barresi”, University of Messina, Messina, Italy

##### **Correspondence:** Claudio Romano (romanoc@unime.it)

Functional gastrointestinal disorders (FGDIs) are represented by age-dependent, chronic, or recurrent gastrointestinal symptoms, not explained by neither structural nor biochemical abnormalities [1,2], that reduce the quality of life of patients and families, and raise health care consultation and related costs. According to the revised diagnostic Rome IV criteria, childhood FGDIs can be distinguished in FGDIs of younger (neonate/toddler) and older children (child/adolescent) [1,2]. FGDIs in neonate and toddler (aged 2-3) include 7 clinical entities, such as infant regurgitation, infant rumination syndrome, cyclic vomiting syndrome, infant colic, functional diarrhea, infant dyschezia and functional constipation [1]. For children and adolescents, FGIDs are divided into three main groups: functional nausea and vomiting disorders, functional abdominal pain disorders (FAPDs), and functional defecation disorders [2]. Functional symptoms vary with age, and depend on the stage of the patient’s physiologic, affective and intellectual development [1]. During childhood, they sometimes attend the normal development (for example, infant regurgitation), or they can develop from maladaptive behavioral responses to internal or external stimuli (for example, fecal retention arising from an experience with painful defecation) [1]. Diagnosing FGIDs in infant and preschool child, is mainly based on parental report and clinician observation [1,3]. In older children, all FGIDs can be diagnosed only if an appropriate medical evaluation rule out another underlying medical condition [2]; therefore, patients with organic diseases, such as celiac disease or inflammatory bowel diseases (IBDs) can present “alarm symptoms” as fever, bleeding, vomiting and failure to thrive [3]. The prevalence of most FGIDs in childhood is unknown. The most common FGDIs are regurgitation and colic in infants, functional constipation and functional diarrhea in toddlers, and functional constipation and abdominal migraine in children and adolescents [4,5]. The most successful management of FGIDs in children involve behavioral therapy and counseling families. Psychological factors that can contribute to the severity of the problem should be addressed. Dietary modifications and pharmacological treatment can be useful [1,2]. A large proportion of children with FGDIs continue to satisfy the diagnostic criteria at long-term follow up [6,7]. It has been suggested that particularly FAPDs in childhood may be a precursor to FGIDs in adulthood. Childhood predictors of long-term outcomes could be the severity of gastrointestinal symptoms and the presence of extra-intestinal somatic and depressive symptoms [7]. Additionally, children with FAPDs carry higher risk for anxiety disorders in late adolescence and early adulthood, even if abdominal pain resolves [8].

**References**

1. Benninga MA, Faure C, Hyman PE, St James Roberts I, Schechter NL, Nurko S. Childhood Functional Gastrointestinal Disorders: Neonate/Toddler. Gastroenterology. 2016; 150: 1443–1455.

2. Hyams JS, Di Lorenzo C, Saps M, Shulman RJ, Staiano A, van Tilburg M. Childhood Functional Gastrointestinal Disorders: Child/Adolescent. Gastroenterology. 2016; 150: 1456–68.

3. Koppen IJ, Nurko S, Saps M, Di Lorenzo C, Benninga MA. The pediatric Rome IV criteria: what’s new? Expert Rev Gastroenterol Hepatol. 2017; 11:193-201.

4. van Tilburg MA, Hyman PE, Walker L, Rouster A, Palsson OS, Kim SM, et al. Prevalence of functional gastrointestinal disorders in infants and toddlesr. J Pediatr. 2015; 166:684-9.

5. Lewis ML, Palsson OS, Whitehead WE, van Tilburg MA. Prevalence of Functional Gastrointestinal Disorders in Children and Adolescents. J Pediatr. 2016; 177:39-43.

6. Miele E, Simeone D, Marino A, Greco L, Auricchio R, Novek SJ, et al. Functional gastrointestinal disorders in children: an Italian prospective survey. Pediatrics. 2004; 114:73-8.

7. Horst S, Shelby G, Anderson J, Acra S, Polk DB, Saville BR, et al. Predicting Persistence of Functional Abdominal Pain from Childhood into Young Adulthood. Clin Gastroenterol Hepatol. 2014; 12: 2026–2032.

8. Shelby GD, Shirkey KC, Sherman AL, Beck JE, Haman K, Shears AR, et al. Functional abdominal pain in childhood and long-term vulnerability to anxiety disorders. Pediatrics. 2013; 132:475-82.

## A66 Chronic immune thrombocytopenia in children: new therapeutic options

### Francesca Rossi, Sofia MR Matarese, Felice Corvino, Bruno Nobili

#### Department of Woman, Child and General and Specialist Surgery, University of Campania “Luigi Vanvitelli”, Naples, Italy

##### **Correspondence:** Francesca Rossi (francesca.rossi@unicampania.it)

Immune thrombocytopenia (ITP) in children is defined as an autoimmune disorder characterized by isolated thrombocytopenia in the absence of other causes or disorders that are associated with thrombocytopenia. Newly diagnosed or “acute” ITP is defined as lasting <3 months, “persistent” lasting up to 12 months, and “chronic” lasting beyond 12 months in which a spontaneous remission is not achieved or in which patients do not achieve a response off therapy. Despite the vast majority of children with ITP will experience resolution, one-third of children will demonstrate thrombocytopenia at 12 months post-diagnosis consistent with chronic ITP [1-3].

Currently available therapeutic agents may provide a transient increase in platelet counts. This may be associated with diminished bleeding in some children. Other collateral benefits may be seen in some families such as less parental anxiety and greater support for children to participate in social activities [4].

Treatments for chronic ITP largely overlap with the therapeutic agents utilized for the treatment of acute ITP, including intravenous immunoglobulin, anti-D immunoglobulin, and corticosteroids [5,6]. The list of agents also includes a variety of agents such as vincristine, danazol, mycophenolate mofetil, and dapsone utilized as monotherapy or in various combinations [7, 8]. Rituximab and splenectomy remain as options.However, many of these treatments are not ideal in children with chronic ITP. Platelet response rates and durability of platelet responses with rituximab treatment are variable and the risk of mortality secondary to sepsis and thromboembolic events with splenectomy is low but real [9,10].

The thrombopoietin (TPO) is a lineage-specific cytokine that stimulates the production of megakaryocytes and platelets. Two TPO receptor agonists (TPO-RAs), romiplostim and eltrombopag, are currently Food and Drug Administration (FDA) approved for adults with chronic ITP. Eltrombopag is also approved for children >1 year. In a randomized phase I/II trial of pediatric patients with primary ITP for >6 months, 88% of patients receiving romiplostim maintained a platelet count >50 × 10^9^/l for a median of 7 weeks compared to zero patients in the placebo group [5]. Results from eltrombopag randomized clinical trials showed that approximately 40% of patients were able to achieve a platelet count >50 × 10^9^/l for the majority of study visits compared to 0–3% of patients in the placebo group [11-14].

Currently, TPO-RAs represent a new therapeutic option for children with chronic ITP. In the future, TPO-RAs may offer an important therapeutic option for other thrombocytopenias.

**References**

1. Rodeghiero F, Stasi R, Gernsheimer T, Michel M, Provan D, Arnold DM et al. Standardization of terminology, definitions and outcome criteria in immune thrombocytopenic purpura of adults and children: report from an international working group. Blood. 2009; 113: 2386–2393.

2. Terrell DR, Beebe LA, Vesely SK, Neas BR, Segal JB, George JN. The incidence of immune thrombocytopenic purpura in children and adults: a critical review of published reports. Am J Hematol. 2010; 85: 174–180.

3. Neunert CE, Buchanan GR, Imbach P, Bolton-Maggs PH, Bennet CM, Neufeld E et al. Intercontinental Cooperative ITP Study Group Registry II Participants Bleeding manifestations and management of children with persistent and chronic immune thrombocytopenia: data from the Intercontinental Cooperative ITP Study Group (ICIS) Blood. 2013; 121: 4457–4462.

4. Neunert C, Lim W, Crowther M, Cohen A, Solberg L Jr, Crowther MA. The American Society of Hematology 2011 evidence-based practice guideline for immune thrombocytopenia. Blood. 2011; 117: 4190–4207.

5. El Alfy MS, Mokhtar GM, El-Laboudy MA, Khalifa AS. Randomized trial of anti-D immunoglobulin versus low-dose intravenous immunoglobulin in the treatment of childhood chronic idiopathic thrombocytopenic purpura. Acta Haematol. 2006; 115:46–52.

6. Hedlund-Treutiger I, Henter JI, Elinder G. Randomized study of IVIg and high-dose dexamethasone therapy for children with chronic idiopathic thrombocytopenic purpura. J Pediatr Hematol Oncol. 2003; 25:139–144.

7. Miano M, Ramenghi U, Russo G, Rubert L, Barone A, Tucci F et al. Mycophenolate mofetil for the treatment of children with immune thrombocytopenia and Evans syndrome. A retrospective data review from the Italian association of paediatric haematology/oncology. Br J Haematol. 2016; 175:490–495.

8. Patel AP, Patil AS. Dapsone for immune thrombocytopenic purpura in children and adults. Platelets. 2015; 26:164–167.

9. Cooper N. State of the art - how I manage immune thrombocytopenia. Br J Haematol. 2017; 177:39-54.

10. Chater C, Terriou L, Duhamel A, Launay D, Chambon JP, Pruvot FR et al. Reemergence of Splenectomy for ITP Second-line Treatment? Ann Surg. 2016; 264:772-777.

11. Bussel JB, Buchanan GR, Nugent DJ, Gnarra DJ, Bomgaars LR, Blanchette V et al. A randomized, double-blind study of romiplostim to determine its safety and efficacy in children with immune thrombocytopenia. Blood. 2011; 118:28–36.

12. Grainger JD, Locatelli F, Chotsampancharoen T, Donyush E, Pongtanakul B, Komvilaisak P, et al. Eltrombopag for children with chronic immune thrombocytopenia (PETIT2): A randomised, multicentre, placebo-controlled trial. Lancet. 2015; 386:1649–1658.

13. Bussel JB, Garcia de Miguel P, Despotovic J, Grainger JD. Eltrombopag for the treatment of children with persistent and chronic immune thrombocytopenia (ITP): Results from the randomised, multicentre, okacebo-controlled PETIT study. Lancet Haematol. 2015; 2:315–325.

14. Bussel JB, Hsieh L, Buchanan GR, Stine K, Kalpatthi R, Gnarra D et al. Long-term use of the thrombopoietin-mimetic romiplostim in children with severe chronic immune thrombocytopenia (ITP). Pediatr Blood Cancer. 2015; 62:208–213.

## A67 Respiratory health starts in pregnancy

### Franca Rusconi (f.rusconi@meyer.it)

#### Unit of Epidemiology, Anna Meyer Children’s University Hospital, Florence, Italy

Starting from the work of DJP Barker “The fetal and infant origins of adult disease. The womb may be more important than the home”, there has been growing evidence of the role of the prenatal environment in the development of childhood respiratory disorders. One of the well-known risk factors implicated in the development of wheezing, asthma and diminished lung function is maternal smoking during pregnancy. In the last few years, however, the importance of other factors has been acknowledged.

We provide an overlook of selected conditions that are relatively prevalent among reproductive age women and for which there has been a relatively large literature in the last few years of a relationship with respiratory disorders in offspring.

For the selected conditions on which we provide an overview, specifically hypertensive disorders of pregnancy, overweight and obesity, infections and antibiotics use, maternal stress and cesarean section, we will discuss potential mechanisms, as well as mediators and confounding factors involved in associations. This is important, as any prevention strategy aimed at reducing the burden of childhood respiratory health must act upon risk factors that are in the casual chain.

Several birth cohort studies established in the past two decades and prospectively collecting data on pregnancy and perinatal exposures are precious source of data on potential risk factors for the development of respiratory outcomes in childhood. Wheezing and asthma, which affect up to 30 % of children, have been largely studied in cohort studies, but also less common respiratory problems and specifically bronchopulmonary dysplasia (BPD), a complication affecting up to 35% of infants born very preterm, will be the focus of the present review.

## A68 Vaccination against ACWY meningococcal disease in Italy

### Rocco Russo (roccorusso@tin.it)

#### Maternity and Pediatrics Services–Local Health Units, Benevento, Italy

Meningococcal disease is caused by Neisseria meningitidis, an encapsulated bacterium whose pathogenic strains are divided into serogroups based on components of the polysaccharide capsule.

In Italy in 2015, 196 cases of invasive meningococcal disease were reported, with an incidence of 0.32 cases per 100,000; the incidences are increasing compared to previous years (0.23 in 2012, 0.29 in 2013, and 0.27 in 2014). In most regions the trend is almost stable or presents small fluctuations in the period from 2011-2014. The exception is the region of Tuscany, where both the consolidated 2015 data and the preliminary 2016 data show a marked increase of cases of meningococcal type C in adults, which has led to the implementation of a major vaccination campaign by the region.

The incidence of invasive meningococcal disease in Italy is higher in the 0-4 year-old age group and especially in the first year of life, when the incidence exceeds 4 cases per 100.000. Nevertheless, the incidence remains high up to 15-24 years of age and decreases from the age of 25 and up.

In 2015 the number of Italian cases of invasive meningococcal disease divided by serogroup and the isolation percentage, serotyped within the total number of reported cases, are 142 with the following distribution: serogroup A (n. 0 (0%)), serogroup B (n. 49 (36%)), serogroup C (n. 63 (44%)), serogroup W (n. 7 (5%)), serogroup Y (n. 23 (14%)) [1].

The number of reported infections for which information on the capsular serogroup isn’t available (approximately 30%) remains high.

In Italy, for the prevention of meningococcal infections of serogroups A, C, W, and Y, the current National Immunization Prevention Plan 2017-2019 provides for a dose of the monovalent meningococcal C conjugate vaccine for the 13-15 month-old age group. For the 12-14 year-old age group, a dose of quadrivalent meningococcal conjugate vaccine Men ACWY is recommended, both for those who have never had the childhood vaccination C or quadrivalent, and for those who have already received a dose, since the persistence of the protection is tied to a high bactericidal antibody titer, which tends to decrease over time [2].

As an alternative to the anti-meningococcal C vaccine, some Italian regions (for example: Emilia Romagna, Veneto, Campania, Sicily and Puglia), recommend the tetravalent A, C, Y, W135 vaccine for the 13-15 month-old age group. The aim is to give children greater protection for those strains of meningococcus that, while still sporadic in the country, show a tendency to increase, mainly because of climate change, travel, and migration.

**References**

1. Surveillance data of invasive bacterial diseases updated on November 16, 2016. Istituto Superiore di Sanità Italiano (ISS) Available at: http://www.iss.it/binary/mabi/cont/Report_MBI_20161116_v11.pdf. Accessed June 22, 2017.

2. Piano Nazionale Prevenzione Vaccinale 2017-2019. Ministero della Salute Italiano. Available at: http://www.salute.gov.it/imgs/C_17_pubblicazioni_2571_allegato.pdf, Accessed June 22, 2017.

## A69 The new juridical framework for medical malpractice in Italy: main issues for the Italian insurance market

### Paolo Sacchi (Paolo.sacchi@aon.it)

#### Aon S.p.A. Insurance & Reinsurance Brokers, Milano, Italy

A new law, legge 24/2017, bearing “Disposizioni in materia di sicurezza delle cure e della persona assistita, nonché in materia di responsabilità professionale degli esercenti le professioni sanitarie” known as “legge Gelli” – Federico Gelli being the main promoter of this law within Italian Parliament - concerning professional liability in the healthcare sector (both for hospitals and professional) was issued on march 8^th^ 2017. The main features of the law can be summarized as follows: - Health professionals, both freelancers and employees, either public or private, are compelled to purchase a professional liability or gross negligence insurance coverage. - Hospitals, either public or private, are compelled to purchase a third party liability insurance coverage or, as an alternative, they are allowed to retain the risk. This self-insured retention can be partial (applying deductibles – up to € 1.5 million each and every loss – on third party liability insurance policy) or even total. - Patients will be allowed to bring civil actions against health professionals’ insurers and hospitals’ insurers. The “Legge Gelli” won’t be entirely and immediately applicable until the “decreti attuativi” (expected to be issued by the Parliament within 120 days) will clarify many relevant parts of the law. As Italian insurance companies are largely unavailable to underwrite “medmal” risks, we shall try to understand the effects of the new law on Italian insurance market.

## A70 Society relief and labor, “La Scarpetta” hospital, Pediatric museum

### Giulio Seganti (giulioseganti@gmail.com)

#### Pediatric Neonatologist, Rome, Italy

In 1871 the City Hall of Rome created the "Society for the Reception Hall for the Children of Workers' ", with the aim of helping the "honest and poor mothers" working in factories and workshops. In 1892 A.Celli, a member of the Relief Society and Labor (SRL), founded an outpatient clinic, named "La Scarpetta". Noble and charitable ladies, whose purpose was to assist the poor children and families of Trastevere and the Ghetto, constituted the SRL. In 1894 L. Concetti, professor of Pediatrics at La Sapienza University, used the Scarpetta for his lessons; he opened a department of 6 beds that was increased in 1905 to 12 beds. The Scarpetta was adjoined to the S. Spirito Hospital, which hosted the Pediatrics Clinic of the University. L.Giordani was director from 1937 to 1961, than A.Seganti became director till 1987. Seganti renewed the SRL statute and restructured the building. In 1968 the hospital reopened with 60 beds. In 1975 La Scarpetta was ceded to public administration and was joined to the “New Queen Margherita”. Subsequently, Scarpetta became an outpatient clinic for visits, vaccinations and neuropsychiatric care. M.Assumma and G.Dell'Uomo are now responsible. In 2012, a center for autism was created. In 2016, it became a center for children and adolescents with disorders of the developmental age, directed by Silvia Bracci.

PEDIATRIC MUSEUM

Seganti has always been collecting old instruments: electrocardiographs, microscopes, viennese wax images, photos of the hospital in the early 19th century, an infinity of objects that tell the story of SRL. A beautiful picture of the beginning of the century portrays Concetti visiting the children with a cylinder on the head, to differentiate him from his assistants. In 1992 Seganti had the idea of ​​making a pediatrics museum with an attached library, therefore he donated all his collected material. In 1997 the Museum was set up in the ace of the Scarpetta.

In 1976 the Scientific Committee and SRL organized the PUER: 4 evening conferences taking place at the beginning of the summer every year, till 2008 (32 editions). The Future: in agreement with the RM1, the objective is to open the museum to the public, to valorize its contents by involving the media, to promote and replicate the PUER meeting that all the pediatricians still remember.

This is to relaunch a never forgotten structure of the old Rome, known by Romans as "the historic good living room of Roman pediatrics".

## A71 To become adult with intellectual disability

### S. Tajè, A. Selicorni

#### UOC Pediatria, ASST Lariana, Como, Italy

##### **Correspondence:** A. Selicorni (angelo.selicorni61@gmail.com)

The improvement of pediatric assistance of patients affected with complex diseases associated with intellectual disability (ID) considerably increased their survival. Bittles et al. (2002) stated that mean survival age depends from the severity of mental retardation, so patients with mild ID have a mean survival around 74 years, moderate ID people of 68 and severly ID patients around 58 [1]. The clinical evolution of these patients is quite difficult to define because it’s related both to the natural history of the different syndromes and to the general, transversal problem related to disability. As it’s quite easy to understand syndrome-specific problems are mostly unknown. Moreover recently different research groups have published data related to adult cohorts of patients affected with various genetic syndromes like de Lange [2], Williams, Charge, Costello, Rett, Angelmann and Prader Willi syndrome in order to define age-specific medical complications. Generally speaking two of the main challenges that caregivers have to face with are overweight and behavioural problems. For various reasons (increase psychiatric food intake, poor physical activity) the natural evolution of many patients affected with syndromic ID is toward overweight or obesity with the well- known consequences at cardiovascular and metabolic level. Again for various reasons (poor inclusion, scare communicative skills) it’s also frequent the development of behavioural problems that can be very difficult to treat and to cope with. Two other important issues characterizing adolescents with ID are education to sexuality and to the more independent life possible. For both these situations the help that families usually receive from institutions is really very poor; in this landscape a great job is made from “parents support groups” which are able to organize targeted experiences in order to face with these challenges. Finally adolescence should be the privileged moment in which transition from pediatric specialists to adult ones takes place. In USA a dedicated protocol suggested from AAP is available [3]. Unfortunately in our country we have no defined recommendations and it’ also very difficult to identify proper specialists to address these patients. So most of these families remain connected for a while to pediatric centers or are lost to follow-up. The definition of a national guideline related to transition of care for these patient is absolutely urgent and mandatory.

**Acknowledgments**

We thank Fondazione Mariani, Milan for supporting clinical activity related to pediatric clinical genetics at UOC Pediatria, ASST Lariana

**References**

1. Bittles AH, Petterson BA, Sullivan SG, Hussain R, Glasson EJ, Montgomery PD. The influence of intellectual disability on life expectancy. J Gerontol A Biol Sci Med Sci 2002; 57: M470-2.

3. Mariani M, Decimi V, Bettini L, Maitz S, Gervasini C, Masciadri M, et al. Adolescents and adults affected by Cornelia de Lange syndrome: A report of 73 Italian patients. Am J Med Genet C Semin Med Genet 2016; 172:206-13.

3. American Academy of Pediatrics, American Academy of Family Physicians, American College of Physicians, Transitions Clinical Report Authoring Group. Supporting the health care transition from adolescence to adulthood in the medical home. Pediatrics 2011; 128:182-200.

## A72 Only exceptional circumstances, only regenerative tissue

### Stefano Semplici (semplici@lettere.uniroma2.it)

#### Department of Literature, Philosophy and History of Art Studies, University of Rome Tor Vergata, Rome, 00173, Italy

The respect for fundamental rights and human dignity entails the respect for personal integrity. This is why the case of organ and tissue removal from living donors has always been considered both as a shining example of solidarity and one of the most risky situations for the most vulnerable individuals to be exploited. According to the Oviedo Convention, “the human body and its parts shall not, as such, give rise to financial gain” (Art. 21) and three conditions are to be met: therapeutic benefit of the recipient; no suitable organ or tissue available from a deceased person and no other alternative therapeutic method of comparable effectiveness; free and informed consent (Art. 19). Therefore, no controversy should arise as to the “protection of persons not able to consent” (Art. 20), including children: prohibition is the obvious consequence. The only exception concerns “regenerative tissue” and was included, as it is made explicit in the Explanatory Report, to permit removal of bone marrow from a minor for the benefit of his or her brother or sister, provided that the potential donor does not object. This provision builds on two essential considerations, which are worth reflection in a broader perspective. First, life-saving potential for the recipient, which is also a necessary condition, has to be predicated on “acceptable” risk (*ER* 127). This balancing between the principle of mutual aid and the protection of personal integrity, given the lack of the “capacity” to consent, requires not only that the opinion of the minor be increasingly taken into consideration, but also setting in a very precautionary way the bar of acceptable risk. Research involving children and adolescents is confronted with a similar responsibility. According to the Ethical Guidelines published by CIOMS in 2016 (of course, the Helsinki Declaration is also to mention), for instance, a “minor increase above minimal risk” may be permitted when a “compelling” social value is at stake (*GL* 17). Secondly, the strict limitation of brother/sister relationship should avoid “family and doctors going to extreme lengths to found a donor at any price” (*ER* 128). Against this background, Art. 15 of the Additional Protocol of 2002, by loosening the restrictions for “cell removal” which implies “minimal risk and minimal burden” for the donor [1], accepts that a quasi-zero risk allows considering other beneficiaries and has in mind future technical developments such as the reconstitution of tissues from a limited number of cells. New ethical challenges, also looking at the risk/benefit evaluation, are likely to arise.

1. Article 14 of the Protocol reaffirms the conditions set in Article 20 of the Convention for removal of regenerative tissue to be performed. Article 2 makes it clear that the provisions applicable to tissues apply also to cells, including haematopoietic stem cells. The Protocol does not apply to reproductive organs and tissue, embryonic or foetal organs and tissues, blood and blood derivatives.

## A73 Child abuse: talking about to fight it

### Andrea Smarrazzo^1^, Paolo Siani^2^

#### ^1^ Department of Translational Medical Sciences, Section of Pediatrics, University of Naples "Federico II", Naples 80137, Italy; ^2^Division of Pediatrics, "Santobono-Pausilipon" Hospital, Naples, 80137, Italy

##### **Correspondence:** Andrea Smarrazzo (and.smarrazzo@gmail.com)

Child abuse, in all its various forms, is one of the most dramatically important causes of morbidity and mortality in children; the amount of cases reported by the literature (as well as from the media and the associations more dedicated to this subject) are impressive, especially considering the important underestimation of the cases themselves. A feeling of "lack of preparation and helplessness" is a consequence of the lack of this subject in medical education, although its occurrence is extremely and tremendously more frequent than thought. This gave rise to the need to implement our knowledge on the subject, in order to improve our ability to deal with child abuse, recognizing and reporting it. The aim is to provide the clinician with the awareness on the true epidemiology and the different types of child abuse; turning on the light on the problem we’ll start to think that, unfortunately, such a diagnosis is possible. The subsequent management, from reporting to the Judicial Authority, sending the patient to an hospital for a multidisciplinary management and diagnostic-instrumental study, must be part of the cultural baggage of every pediatrician, working in hospital or in the field. It must be remembered that the role of the pediatrician is purely to report the suspicion, leaving the judge the task of ascertaining the facts. Hence the collaboration of Division of Pediatrics of the Santobono-Pausilipon Hospital, the Cultural Association of Pediatricians (ACP) and the School of Specialization in Pediatrics of Naples to study child abuse through meetings and seminars, leading to the drafting of a practical protocol, derived from the most recent evidence of literature. We suggest to diffuse it in pediatric offices, in the Emergency Departments, in the pediatric wards and in the Schools of Specialization in Pediatrics.

## A74 Cybersick!: risks and side effects of digital media use

### Manfred Spitzer (manfred.spitzer@uni-ulm.de)

#### Department of Psychiatry, University of Ulm, Ulm, Germany

Digital information technology (IT) has become part of our everyday life. Drawing from studies in cognitive neuroscience, experimental psychology, education research as well as clinical research, I argue that there is a considerable negative impact of digital IT on mental functioning. Mechanisms include (1) “outsourcing” mental work from our brains into machines, (2) replacing face-to-face contact with digital contact, resulting in reduced empathy towards parents and peers, (3) distractions, such as multitasking and being online most of the time, resulting in dysfunctional attentional and thought processes, (4) giving away the control of our lives to gadgets, thereby increasing chronic stress, with its known negative impact on physical and mental functioning, (5) addiction and (6) lack of exercise and recreational outdoor activities, with its known detrimental physical and social effects. Furthermore, digital IT may cause short-sightedness (myopia), hypertension, diabetes, sleep disorders, depression, attention deficit disorder, and dementia.

With special emphasis on brain development in young age, and cognitive decline in old age, I will present examples to illuminate these processes and mechanisms that cause concern regarding the risks and side effects of the massive digital media use that is the norm in developed societies. In particular, I argue that these effects are long-term in nature and must be taken seriously *now*. Needless to say: I am not against the use of digital information technology per se. But I want to caution against the unrestricted and market-driven exposure of our most precious resource, the brains and minds of the next generation, on a large scale, to devices with strong risks and side effects which are either already known or are suggested by what we know about brain development and functioning.

## A75 What twin studies can add

### Maria Antonietta Stazi, Sonia Brescianini

#### Centre for Behavioural Sciences and Mental Health, Istituto Superiore di Sanità, 00161, Rome, Italy

##### **Correspondence:** Maria Antonietta Stazi (stazi@iss.it)

For more than half a century twin studies have been a milestone in biomedical research. Indeed, they have quantified the relative contribution of genetics and environment not just to the occurrence of many diseases but, more in general, to the development of innumerous human traits, and have provided estimation of their “heritability”. In details, twin research allows to disentangle the effect of genetic sequences and environmental exposures on phenotype variability. Environment could be shared by the twins (e.g. maternal exposure in *utero*) or non-shared (e.g. factors specific to each fetus). This can be done through mathematical models based on the comparison of similarities and differences in monozygotic *versus* dizygotic twins.

In the more recent epigenetic era, that studies mechanisms turning on and off DNA translation into proteins, the role of twin research is even more relevant. Discordant monozygotic twin design (discordant on phenotype or on exposure) is a robust design that allows to compare epigenetic features adjusting for genetic background, early life exposures, age, gender and cohort effects.

For all these reasons, the possibility of following twins from birth onwards has become a powerful tool for research in public health.

Research activities in the pediatric field of the Italian Twin Registry (ITR) [1] will be presented. The ITR has enrolled, since 2001, about 28,000 twins of which about 4,000 are children below 12 years of age. The ITR, participates to the European network of birth cohorts with its newborn twin cohort MUBICOS (MUultiple BIrth COhortS). MUBICOS is a multicenter birth cohort that has enrolled about 360 families and collected data at birth, 6,12, 18 and 36 months of age on different outcomes: growth, respiratory health and allergies, sleeping behavior and neurodevelopment. DNA has been collected for twins and their parents using saliva collection kits, and stored in the ITR Biobank. Part of the DNA from the twins has been used to determine twin zygosity, a necessary information to implement twin studies.

**Reference**

1. Brescianini S, Fagnani C, Toccaceli V, Medda E, Nisticò L, D'Ippolito C, et al. An update on the Italian Twin Register: advances in cohort recruitment, project building and network development. Twin Res Hum Genet. 2013; 16:190-6.

## A76 Short-term and long-term effects of traffic air pollution on school children neurodevelopment

### Jordi Sunyer^1,2,3^ (jordi.sunyer@isglobal.org)

#### ^1^Childhood and Environment program, ISGlobal- Centre for Research in Environmental Epidemiology (CREAL), Barcelona, E-08003, Spain; ^2^Respiratory health, air pollution, childhood development group, IMIM (Hospital del Mar Medical Research Institute), Barcelona, E-08003, Spain; ^3^Experimental and Health Sciences Department, Pompeu Fabra University, Barcelona, E08003,Spain

Exposure to traffic related air pollutants (TRAPs) during pregnancy or infancy has been related to cognitive impairment and behavioral disorders in children preliminarily but there is no evidence on the role of TRAPs in schools on cognitive function and neuroimaging. Children (n=2897) aged 7 to 10 years from 39 high and low TRAPs schools, paired by socio-economic status, were tested via a series of four computerized tests from January 2012 to March 2013 in Barcelona (Catalonia, Spain) to evaluate working memory development, executive attention, impulsivity, and selective attention. Behavioral problems (strengths and difficulties questionnaire) were reported by parents. Attention Deficit and Hyperactivity Disorder (ADHD_DSM IV) was reported by teachers. MRI (T2, flair, and DTI) and fMRI were conducted in around 300 children. Air pollution (nitrogen dioxide (NO2), ultrafine particle number (UFP), and particulate matter (PM) < =0.25 μm (quasi-ultrafines), 0.25 to 2.5 μm (accumulation mode), 2.5 to 10 μm (coarse mode), < =2.5 μm (PM2.5) and organics (PAHs)) was measured during two, one week campaigns both inside the classroom and in the courtyard simultaneously in each school pair during 2012. The children performed 10,973 cognitive tests. Cognitive functions increased notably (around 10% per year) during primary school years. Children attending schools with higher TRAPS (largely diesel pollutants such as EC and UFP), had a smaller improvement with age in cognitive development (in all measured cognitive functions) [1]. Similarly, TRAPS were associated with more frequent behavioral problems [2]. Only fine particles generated from traffic (no from other origins) showed the association with brain development [3]. TRAPS were associated with lower functional integration and segregation in key brain networks using neuroimaging which indicates slower brain maturation [4]. These chronic relationships were independent of the acute effects, though the short-term exposures to TRAPs (the day before) were also associated with daily fluctuations in attention [5]. Furthermore, noise inside the classroom is related to attention deficit and hyperactivity disorder symptoms, but the effects of TRAPS were independent of noise. In addition, we proved that green space is beneficial for brain maturation (function and structure) and that moderate video-gaming is beneficial for brain functioning but at certain level is related to behavioral problems. Overall, school air is relevant for a healthy brain development. Results imply cost-benefit interventions in schools to endorse the protection of child brain maturation from traffic exhausts.

References

1. Sunyer J, Esnaola M, Alvarez-Pedrerol M, Forns J, Rivas I, López-Vicente M, et al. Association between traffic-related air pollution in schools and cognitive development in primary school children: a prospective cohort study. PLoS Med. 2015; 12:e1001792.

2. Forns J, Dadvand P, Foraster M, Alvarez-Pedrerol M, Rivas I, López-Vicente M, et al. Traffic-Related air pollution, noise at school, and behavioral problems in Barcelona schoolchildren: a cross-sectional study. Environ Health Perspect. 2016; 124:529-35.

3. Basagaña X, Esnaola M, Rivas I, Amato F, Alvarez-Pedrerol M, Forns J, et al. Neurodevelopmental deceleration by urban fine particles from different emission sources: a longitudinal observational study. Environ Health Perspect. 2016; 124:1630-1636.

4. Pujol J, Martínez-Vilavella G, Macià D, Fenoll R, Alvarez-Pedrerol M, Rivas I, et al. Traffic pollution exposure is associated with altered brain connectivity in school children. Neuroimage. 2016; 129:175-84.

5. Sunyer J, Suades-González E, García-Esteban R, Rivas I, Pujol J, Alvarez-Pedrerol M, et al. Traffic-related air pollution and attention in primary school children: short-term association. Epidemiology. 2017; 28:181-189.

## A77 The Transitional Care: a national project

### Domenico Tangolo (domenico.tangolo@unito.it)

#### ASTRA, Association for Health in Transitions, Torino, Italy

**Background**

In recent years there has been a growing need to ensure the planning and the structure of care pathways that allow a gradual transition from the pediatric age to adulthood due to the gradual increase of patients with chronic diseases during adolescence. However, the medical importance of these diseases has not increased hand in hand with the development of competences in the field of medicine for adults; therefore, these complex patients have difficulties to be inserted in the organizational structure of medicine for adults.

As regards socio and health care, the scientific literature demonstrates that the type of care offered within the pediatric area and the one addressed to adults is profoundly different. For these reasons, we note the existence of a considerable heterogeneity of treatment. Main goal of the project is to identify the "winning" characteristics of transition models (from pediatric to adulthood) in which the planning of transition and the involvement in all stages of transition of patients and the family are inalienable variables of the transitional care pathway.

**Materials and Methods**

The program of work consists of three main phases that run "in parallel": modeling, trial, mapping of experiences. Modeling: a direction group provides the task of formulating working hypotheses and quality requirements of a "transition services" independent of the condition being treated. The working hypotheses are subjected to a critical evaluation of the Technical Advisory Committee to assess their feasibility and the theoretical efficiency. A preliminary document of Criteria for the definition of the transition project has been drawn and discussed in three consecutive meetings and with Delphi method. Trial: in this phase, the authors identify some regions in which institutional and university partners are made available both to design concrete organizational models and to define new training methods for the spread of the project. Mapping of the experiences: a specific questionnaire has been designed to be extensively handed out among interested personnel. The project implies the involvement of many partners, among which are considered priorities: institutions, University, Federations of Professional Orders, Scientific Societies, People, Patients and Families associations.

**Conclusions**

The “Transitional Care” is a multidisciplinary and multiprofessional project that consider the complexity of the transitional disease as an opportunity for the implementation and validation of innovative care models in which all protagonists work together for the improvement of heath care delivery.

## A78 Gender medicine in pediatrics

### Walter Malorni^1^, Isabella Tarissi de Jacobis^2^, Lucrezia Gambardella^1^, Giulia Ceglie^2^, Francesca de Gennaro^2^, Elisabetta Straface^1^

#### ^1^Center for Gender-Specific Medicine, Istituto Superiore di Sanità, Rome, Italy; ^2^DPUO-ospedale Pediatrico Bambino Gesù, Rome, Italy

##### **Correspondence:** Walter Malorni (malorni@iss.it)

Gender Medicine (GM) investigates different characteristics of diseases occurring in males and females taking into account both biological (sex-related) and sociocultural (gender-related) features. GM is finalized to improve the appropriateness of care: from diagnostics to the therapy effectiveness. While in adults gender differences have been highlighted in many pathologies (neurological, cardiovascular, immune and infectious diseases), in pediatric age gender disparity is still poorly investigated. Although, gender can influence the possibility to develop specific pathologies even from the fetal and early life stages. In the United States, the National Institute of Health (NIH) has recommended to study gender differences from cradle in all human diseases and several research groups are nowadays being involved in the study of gender-differences worlwide and in Italy too. Specific studies and meta-analysis highlighted that differences in the prevalence of some congenital pathologies can occur in pediatric age. For instance, heart diseases occur most frequently in females (51.3% *vs* 48.7%) in which they are associated with genetic syndromes and extra-cardiac malformations, whereas some neurological disorders, such as autism (ratio 4:1) and Attention Deficit / Hyperactivity Disorder (ADHD) are more common in males. For example, in the overactive/ impulsive period (3-4 years) of ADHD, the ratio males:females observed is 10:1. In infancy, males also appear to be suffering from respiratory distress syndrome, asthma, and lung disease significantly more than females. Females are predisposed to autoimmune pathologies such as childhood multiple sclerosis (2:1 F:M), celiac disease (2:1 F:M) and systemic lupus erythematosus (5-6:1 F:M; 9:1 in adults). For many infectious diseases the incidence is greater in males. For example, viral infections are more frequent in males, but the course of the disease is worse in females. Females have a greater antibody response to vaccines and, more generally, an immune response, both humoral and cell-mediated, more pronounced and prolonged than males. This protects them from infections, but exposes them to a greater risk of developing autoimmune and inflammatory pathologies, as well as adverse reactions. Even with regard to tumors, such as Hodgkin and non-Hodgkin lymphomas or some leukemic forms, the incidence is different (often higher in males). In conclusion, a re-evaluation of the gender issue in pediatric research could be crucial and contribute to the understanding of the pathogenetic mechanisms and the improvement of diagnostic and therapeutic strategies as well as to the improvement of the appropriateness of the cure.

## A79 What is meant by "Humanization of care"

### Marina Tripodi (marinatripodi8@gmail.com)

#### Pediatrics section, Department of Medicine and Surgery “Scuola Medica Salernitana” University of Salerno, 84131, Salerno, Italy

Humanization of Care is the process that places the patient in his/her totality at the center of the care. In Pediatrics, humanization provides an assistance which is focused not only on the child as a patient, but on the whole family as well. The basics of the humanization of pediatric care have been laid with the first charter of the child's rights in the hospital, drawn up in 1988 in Leiden (Holland) by the European Association for Children in Hospital [1]. Other associations have drawn up, over the years, other rights charters, adapting them to most recent necessities [2,3]. From the literature, the main humanization programs for hospital care have been developed in the American continent (Brazil and USA), and in Europe. In Brazil, the pediatric aspect is part of the National Humanization Policy that aims to create a transversal culture of humanization, improving the reception and care of the patient of all ages and social classes and their families [4]. In USA, the current reference model for pediatric care is the Patient and Family Centered Care that according to the American Academy of Pediatrics, is “an innovative approach to the planning, delivery, and evaluation of health care that is grounded in a mutually beneficial partnership among patients, families, and providers that recognize the importance of the family in the patient’s life” [5]. In Europe, in 2011, the Committee of Ministers of the Council of Europe adopted the guidelines for “Child-friendly health care” (CFHC), defined as a health policy focusing on children's rights, needs, characteristics, activities and developmental skills, taking into account their views [6]. Still in Europe, in 2013 the “Think and Action Tank on the Child right to health”(TAT) was founded. It is supported by the European Pediatric Association with the objective of exploring theories, knowledge and experiences aiming at translating the principles of the rights of the child, social justice and equity into practice [7].

The large number of literature articles dealing with humanization issues have rarely measured the outcomes of the interventions carried out. Their real effectiveness for realizing health care programs tailored on the child and his/her family needs remains therefore often unproven. A pilot project recently started in Campania region (Italy), aims to improve aspects of reception, hospitalization and discharge of pediatric patients by networking families, hospitals and family pediatricians. All the actions have been planned after having measured baseline existing and perceived needs of all the players [8].

**Acknowledgements**

We acknowledge the "Campania Region - Program Objectives - year 2013" (Project Line 8 "Development of Humanization Processes in Medical Care Paths"/"Analysis and Implementation of the Humanization Processes in the Pediatric Care Facilities of the Campania Region").

**References**

1. EACH Charter. Available in https://www.each-for-sick-children.org/each-charter. Accessed in May 9 2017.

2. Aopi.it. AOPI - Associazione Ospedali Pediatrici Italiani. Available in http://www.aopi.it/. Accessed in May 9 2017.

3. Fondazione ABIO Italia Onlus. Available in http://www.abio.org/. Accessed in May 9 2017.

4. Moreira MA, Lustosa AM, Dutra F, Barros Ede O, Batista JB, Duarte MC. Public humanization policies: integrative literature review. Cien Saude Colet. 2015; 20:3231-42.

5. Committee on hospital care and institute for patient-and family-centered care. Patient- and Family-Centered Care and the Pediatrician’s Role. Pediatrics. 2012; 129:394-404.

6. Guidelines of the Committee of Ministers of the Council of Europe on child-friendly health care, 21sett 2011. Available in http://www.coe.int/en/web/children/child-friendly-healthcare. Accessed in 9 May 2017.

7. TAT, Think and Action Tank on the Child Right to Health. Available in http://ilariasimonelli79.wixsite.com/think-and-action. Accessed in May 9 2017.

8. Portale Umanizzazione Cure Pediatriche. Available in http://pedianetcampania.it/. Accessed in May 9 2017.

## A80 Celiac disease tomorrow

### Riccardo Troncone (troncone@unina.it)

#### Department of Pediatrics & European Laboratory for the Investigation of Food-Induced Diseases, University Federico II, Naples, Italy

The concept of celiac disease (CD) has radically changed over the last decades. In fact, nowadays CD is no more considered just as an intestinal disease, but as an immune mediated systemic disorder elicited by gluten and related prolamines of barley and rye. It is characterized by a variable combination of gluten related clinical symptoms, CD specific antibodies, HLA DQ2 and/or DQ8 haplotype and enteropathy [1].

Recently, it has emerged that the histological presentation can also include cases of minimal intestinal damage (potential celiac disease), with important problems of diagnosis and management [2].

A great heterogeneity is also appreciated in the immunological mechanisms that underlie the disease, in particular the cytokines involved. Differences could be explained by different exogenous factors (e.g. viruses) that are involved in the development of the disease [3]. The recognition of this heterogeneity will probably lead in future to a better sub-phenotyping of patients and to personalized approaches of prevention and therapy.

The development of new therapeutic strategies represents another field of research [4]. It has been demonstrated that gluten, because of its high content in prolines, is very resistant to pancreatic and brush border enzymes digestion. Consequently, the use of bacterial and fungal proteases has been proposed to induce a complete gliadin degradation and to eliminate toxic epitopes. The identification of specific peptides able to induce both a T-cell and a non T-cell mediated response has allowed to start genetic engineer programs to produce grains without these peptides. The identification of toxic epitopes can also represent the target of new immune-modulatory therapies. Other promising therapeutic perspectives are founded on the blockage of the adaptive immune response through the inhibition of the HLA mediated antigen presentation or gliadin deamination by the tissue transglutaminase.

**References**

1. Husby S, Koletzko S, Korponay-Szabó IR, Mearin ML, Phillips A, Shamir R, et al. European Society for Pediatric Gastroenterology, Hepatology, and Nutrition Guidelines for the Diagnosis of Coeliac Disease. J Pediatr Gastroenterol Nutr. 2012; 54:136-160.

2. Auricchio R, Tosco A, Piccolo E, Galatola M, Izzo V, Maglio M, et al. Potential celiac children: 9-year follow-up on a gluten-containing diet. Am J Gastroenterol. 2014; 109:913-921.

3. Kupfer SS, Jabri B. Pathophysiology of celiac disease. Gastrointest Endosc Clin N Am. 2012; 22:639-660.

4. Kaukinen K, Lindfors K, Mäki M. Advances in the treatment of coeliac disease: an immunopathogenic perspective. Nat Rev Gastroenterol Hepatol. 2014; 11:36-44.

## A81 Why a round table on the humanization of pediatric care in hospital and in primary care?

### Pietro Vajro^1^, Paolo Siani^2^

#### ^1^Pediatrics Section, Department of Medicine, Surgery, and Dentistry “Scuola Medica Salernitana, University of Salerno, Baronissi, Salerno, Italy; ^2^Pediatrics, Santobono, Pausilipon Pediatric Hospital, 80100, Naples, Italy

##### **Correspondence:** Pietro Vajro (pvajro@unisa.it); Paolo Siani (p.siani@santobonopausilipon.it)

It is implied that any effort done to implement the development of humanization processes within the pathways of health care during a pediatric hospitalization and/or in outpatient activities should aim at creating an optimal health and social-medical context placing the patient at the very center.

He/she should be considered in its entirety, taken by hand, and accompanied throughout the diagnostic-therapeutic path, by including of course his/her family as well [1, 2].

Ideally any project in this area should have the ambition to promote structural changes and improve cooperation/coordination among the major stakeholders involved in the care and hospital activities processes, also educating the behavior of all those who are revolving around the hospitalized child.

Indeed, it is still unclear what a unique definition of the term "humanization of care" should be. This may in fact vary very much depending on the latitudes and the socio-economic contexts where it is used, sometimes with an approach of a true national policy (e.g. Brazil) or –rather- of recommendations only (e.g. USA, Europe) (Fig. 1). Involvement of the child and of his/her family, recognition of his/her right to stay during hospitalization in an appropriate environment, by limiting as far as possible the trauma of illness and pain, are in any case a must.

**Fig. 1:**
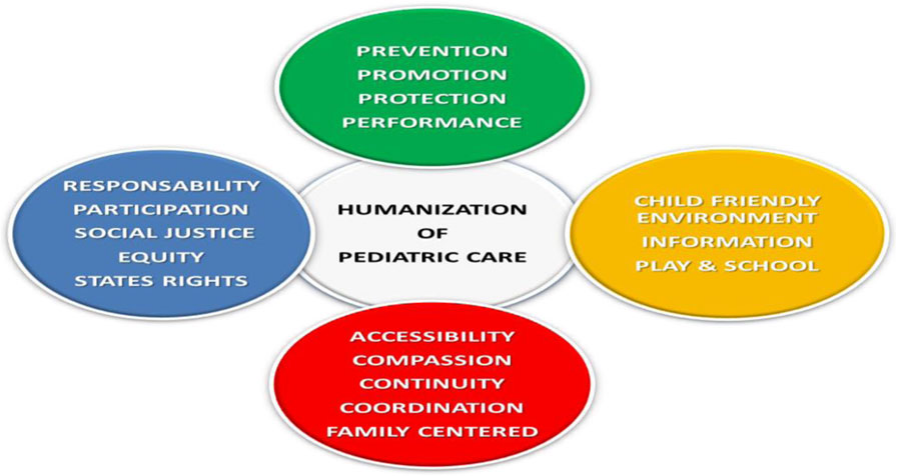
**(abstract A81).** Synopsis of the principles underlying the humanization of pediatric care

The actions taken in hospital are/should always be characterized by a minimum common denominator represented by the need to know by hand the baseline "where we are now" and "what needs to be done" information to improve the existing state of art in order to ensure accessibility and equality of care for all children, regardless of social class, nationality, and religion.

In this regard, a number of helpful specifically designed evaluation tools do exist to measure not only the existing but also the perceived humanization, sometimes revealing a number of surprising differences between the experience of the operators and the users, and -in the latter- between family and patient himself, with considerable distinction also by age, i.e. younger patients vs. teenagers.

What can/should be done to improve the degree of healthcare humanization, starting from the hospital reception to the discharge phases, and ensuring finally a virtuous dialogue with his/her family pediatrician with precise, written indication? The literature is rich in examples of interventions implemented locally by exploiting the help that can come from structural adjustments and also from issues such as technology, better pain management, pet/clown therapy, loud reading, as well. The following summaries in this issue of IJP will outline more on these specific aspects.

**References**

Committee on Hospital Care and Institute for Patient- and Family-Centered Care. Patient- and family-centered care and the pediatrician's role. Pediatrics. 2012; 129:394-404.

Guidelines of the Committee of Ministers of the Council of Europe on child-friendly health care, 2011. Available at https://rm.coe.int/168046ccef. Accessed June 22, 2017.

## A82 Arranged marriage and migration

### Giovanni G. Valtolina (giovanni.valtolina@unicatt.it)

#### Department of Psychology, Catholic University of the Sacred Hearth, Milan, Italy

The matchmaking processes of arranged marriages in the migration context need to be clarify. It is important to recognise the shift from the “consensual” arranged marriages – as from the home country of the parents – to the “forced” arranged marriage, as from the Western migration country. In Western countries, all child marriages are considered forced marriages, as it is highlighted in several international documents (Council of Europe, European Parliament, UN). Generally speaking, it is considered a “consensual” arranged marriage when the family choose the spouse, but the offspring is free to accept or not. In such a marriage, frequent in many immigrant communities, family take the lead, but the ultimate choice to marry remains with the individual. In other words, an arranged married is not necessarily a forced marriage, since the offspring can recognize the traditional authority of the family and accept it. In immigration countries, the conflict between the home country culture and the process of acculturation very often set off a stiffening of the traditional cultural practices, usually due to the fear to lose the original cultural identity. Such a defensive hardiness pushes the husband to take control over wife and daughters, imposing traditional attitudes and behaviors. In this way, a cultural practice - accepted and shared in the home country - becomes a violence and an overwhelming obligation. The Natcen study [1] shows eating disorders and self-mutilation as major clinical consequences of force marriage. Chandler [2], studying suicidal behaviour in migrant girls from South Asia countries, highlights forced marriage as one of the most relevant cause of suicide. A sensitive issue linked to psychological consequences of forced marriage is also the attitude of the specialist who get to know about such a situation regarding a patient. Research shows that specialists have to choose between two options: report the situation to the authority, starting an interethnic quarrel, or consider it as a “cultural practice” similar to others [3]. The phenomenon of forced marriage for the second generation in Europe reached relevant numbers in the last years and the trend is expected to get worst, because of the increase of the “native” second generation, without significant cultural bond with the parents’ home countries. In Italy, the phenomenon of forced marriages has grown exponentially mainly with the increasing immigration of the families coming from Arabic countries and from the Indian subcontinent. For methodological reasons, it is difficult - if not impossible – to quantify this phenomenon. The victims represent a "hidden population", and very often they are reticent and refuse to talk about their private life. In particular, there is a strong resistance in forced marriage victims to denounce members of their family or community, contributing to maintain such phenomenon invisible. So far, in Italy there are no official statistics on forced arranged marriage. Data provided by Unicef (2013) about the percentage of children married before 15 and before 18 in different countries - if combined with the data provided by Istat and Ministry of Interior about non-EU residents in Italy - can help in estimating the population at risk. The communities most at risk in Italy are: Morocco, Albania, some South-East Asian countries (Bangladesh, Pakistan, India, Sri Lanka) and some African countries (Senegal, Ghana, Nigeria, Egypt).

References

1. Natcen Social Researches, Forced marriage: Prevalence and service responses in United Kingdom, 2009. Available in http://natcen.ac.uk/our-research/research/forced-marriage. Accessed June 22, 2017.

2. Chantler K. Recognition of and intervention in forced marriage as a form of violence and abuse. Trauma Violence Abuse. 2012; 13:176-183.

3. Batsleer J, Burman E, Chantler K, Pantling K, McIntosh H, Smailes S, et al. Culture as a barrier to service provision and delivery: domestic violence services for minoritized women. 2002; Manchester, Manchester University Press.

## A83 N-3 polyunsaturated fatty acids: fish, functional foods or supplements

### Elvira Verduci, Carlotta Lassandro, Marta Brambilla, Sara Vizzuso, Benedetta Mariani, Giuseppe Banderali

#### Department of Pediatrics, San Paolo Hospital, Department of Health Sciences, University of Milan, Italy

##### **Correspondence:** Elvira Verduci (elvira.verduci@unimi.it)

Long chain n-3 polyunsaturated fatty acids (n-3 LCPUFAs) are essential for optimal neuronal development as they contribute to membrane fluidity and neuronal plasticity. As highlighted by the European Food Safety Authority (EFSA), brain accumulates large amounts of docosahexaenoic acid (C22:6 n-3, DHA) especially during the first two year of life, contributing to normal brain development [1]. Moreover, as DHA contributes also to the visual development of infants, an association between the intake of infant and follow-on formula supplemented with DHA (at least 0.3% of the total fatty acids (FA)) and visual function at 12 months has been observed [2]. Alfa-linolenic acid (C18:3 n-3, ALA), an essential dietary fatty acid, is the precursor of all n-3 LCPUFAs. Humans can convert ALA to eicosapentaenoic acid (C20:5, EPA) and DHA, but, since conversion efficiency is low, an adequate dietary intake is required [3]. Significant amounts of EPA and DHA characterize fish and derivative fish oil, especially salmon, tuna, mackerel, anchovy, and sardines, while ALA can be found in vegetable oils. Fish is an excellent source of energy, high-biological value and highly digestible proteins, minerals (iodine, selenium, calcium, iron, magnesium, phosphorus), vitamins (vitamin A, vitamin D, vitamin E and vitamins B) and n-3 LCPUFAs. Few data on fish consumption in pediatric age are present. In infants, DHA status tends to decline during the complementary period because the intake of breast milk or formula supplemented with LCPUFA decreases. However, it has been observed that each 10-g increment in fish intake is associated with a 0.3 FA% increase in DHA status. Moreover, during this period DHA-enriched egg yolk or supplemented follow-on formula may have some positive effects on short-term visual function [4]. On the market there are several EPA and DHA supplements derived from fish oils or microalgae. Results from a randomized, double-blind, placebo-controlled trial, on 1160 healthy neonates randomized to receive supplementation with DHA or placebo during the first year of life, showed that the time to achievement of sitting without support was shorter in infants who received DHA while no persistent later motor development milestones was observed [5]. Considering that an adequate intake of 100 mg/die of DHA is recommended during the first 2 years of life [6], if the diet alone or including functional foods does not permit to reach this intake level, a specific DHA supplementation may be considered.

References

1. European Food Safety Authority. Scientific Opinion on the substantiation of a health claim related to DHA and contribution to normal brain development pursuant to Article 14 of Regulation (EC) No 1924/2006. EFSA Journal 2014; 12:3840.

2. European Food Safety Authority. DHA and ARA and visual development - Scientific substantiation of a health claim related to docosahexaenoic acid (DHA) and arachidonic acid (ARA) and visual development pursuant to Article14 of Regulation (EC) No 1924/2006. EFSA Journal 2009; 941:1-14.

3. Abedi E, Sahari MA. Long-chain polyunsaturated fatty acid sources and evaluation of their nutritional and functional properties. Food Sci Nutr. 2014; 2:443–463.

4. Fewtrell M, Bronsky J, Campoy C, Domellöf M, Embleton N, Fidler Mis N, et al. Complementary Feeding: A Position Paper by the European Society for Paediatric Gastroenterology, Hepatology, and Nutrition (ESPGHAN) Committee on Nutrition. J Pediatr Gastroenterol Nutr. 2017; 64:119-132.

5. Agostoni C, Zuccotti GV, Radaelli G, Besana R, Podestà A, Sterpa A, et al. Docosahexaenoic acid supplementation and time at achievement of gross motor milestones in healthy infants: a randomized, prospective, double-blind, placebo-controlled trial. Am J Clin Nutr. 2009; 89:64-70.

6. European Food Safety Authority. Scientific Opinion on Dietary Reference Values for fats, including saturated fatty acids, polyunsaturated fatty acids, monounsaturated fatty acids, trans fatty acids, and cholesterol. EFSA Journal 2010; 8:1461.

## A84 Impact on hospitalization for rotavirus gastroenteritis and intussusception four years after introduction of universal Rotavirus vaccination in Sicily

### Francesco Vitale, Fabio Tramuto, Claudio Costantino, Vincenzo Restivo

#### Department of Science for Health Promotion and Mother to Child Care “G. D’Alessandro”, University of Palermo, Palermo, 90127, Italy

##### **Correspondence:** Francesco Vitale (francesco.vitale@unipa.it)

**Background**

Sicily was the first Italian administrative region to introduce, as free and active offer, rotavirus vaccination (RV) to all newborns into regional immunizations schedule in 2013. Despite a vaccination coverage (VC) of 31% after the first year, a reduction in hospitalizations due to rotavirus gastroenteritis (RVGE) of 41% among children aged 0-59 months was recorded [1]. Furthermore, a stable trend of intussusception compared to before anti-RV introduction period was observed [2]. Notwithstanding, fear of adverse reactions was showed to be main barrier to reach higher VC [3].The aim of this study was to analyze the impact of anti-RV on RVGE and intussusception hospitalizations by Sicilian provinces after (2015-2016) and before (2009-2012) universal mass vaccination.

**Materials and methods**

A pre-post study according to European Centre for Disease Prevention and Control protocol [4] was conducted by analyzing data from vaccination registries and hospital discharge records occurred between 1st January 2009 - 31st December 2016. Data during transitional period (1st January 2013 and 31st December 2014) were excluded from the analysis due to a progressive anti-RV availability [4]. Cases of RVGE and intussusception were defined as all hospitalizations with an ICD-9-CM diagnosis code on any position of 008.61 and 560.0, respectively.

**Results**

In Sicily, overall anti-RV coverage was 41% between 2015 and 2016. During the same period there was a reduction in RVGE hospitalization rate of 46% compared with 2009-2012 (213 Vs 393 per 100,000). As expected, RVGE hospitalization rate dropped among provinces with VC higher than 50% (Palermo, Trapani and Agrigento) during 2015-2016 (Fig. 1 and Fig. 2). On the other hand provinces with lower VC (Fig. 1) had lower (Messina) or medium (Enna and Caltanissetta) RVGE hospitalization rate reduction (Fig. 2). Moreover, intussusception hospitalization rate (2015-2016 Vs. 2009-2012) showed a trend not related to VC (Fig. 3), especially in the province of Messina, where to a low VC corresponded a relevant increase in intussusception hospitalization rate.

**Fig. 1:**
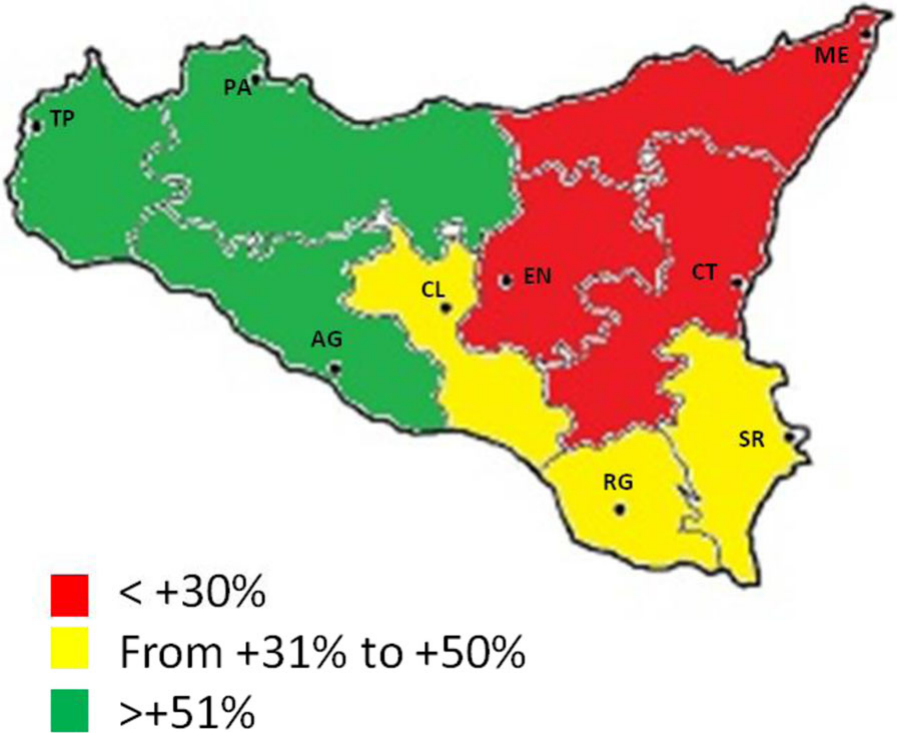
**(abstract A84).** Anti-RV vaccination coverage from 2015 to 2016 in Sicily

**Fig. 2:**
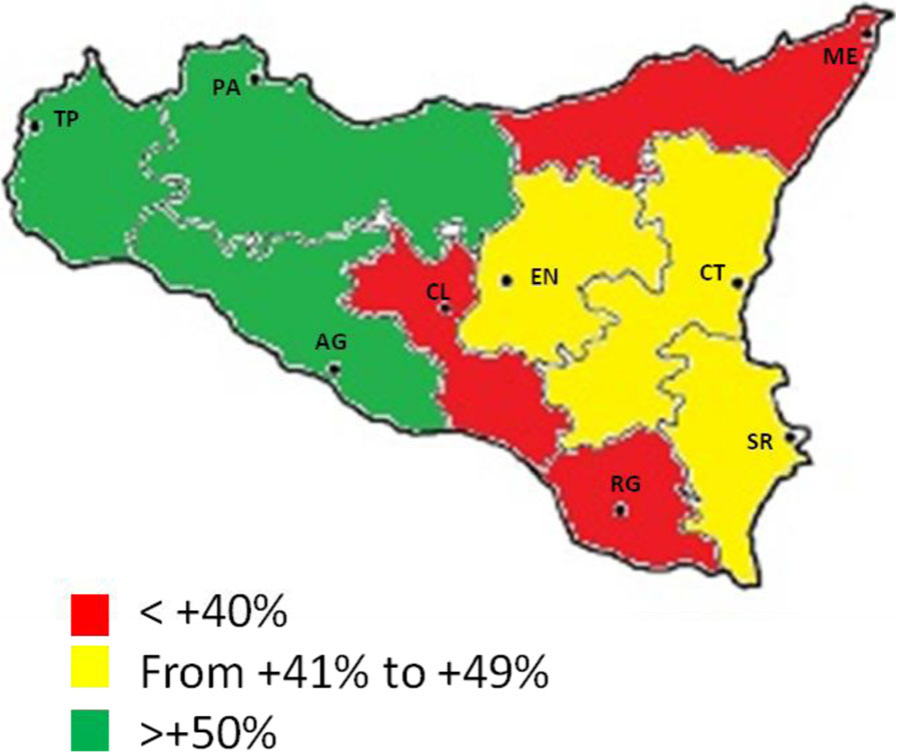
**(abstract A84).** RVGE hospitalization reductionafter (2015-2016) and before (2009-2012)anti-RV vaccine implementation in Sicily

**Fig. 3:**
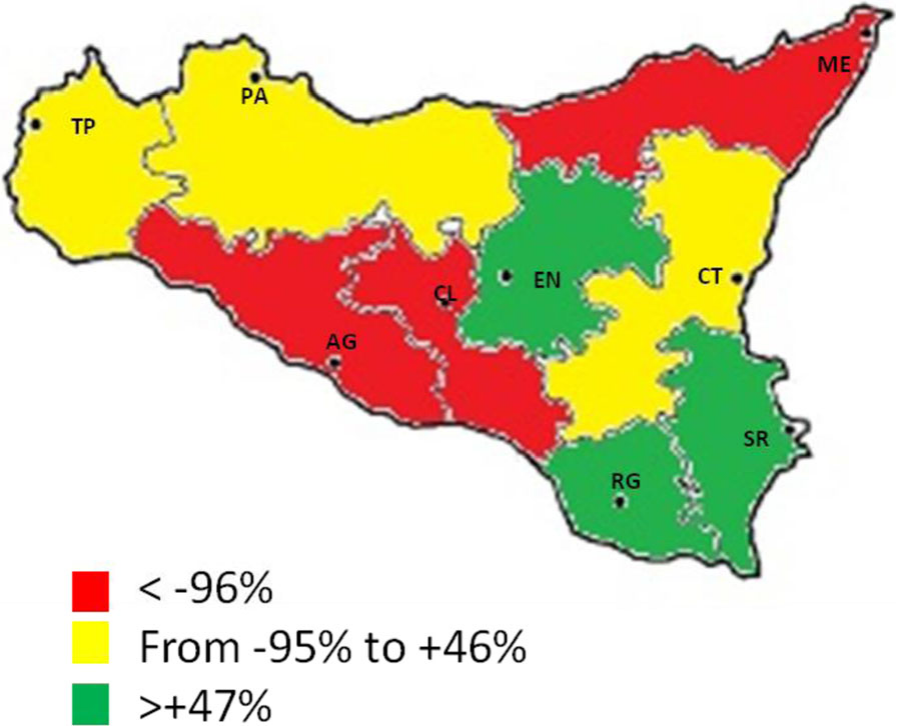
**(abstract A84).** Intussusception hospitalization reduction after (2015-2016) and before (2009-2012) anti-RV vaccine implementation in Sicily

**Conclusions**

This study showed high impact of RV on RVGE rate reduction among Sicilian provinces with VC higher than 50%. Moreover, the trend of intussusception hospitalizations unrelated to VC by province but similar to before anti-RV Sicilian data allows us to confirm the security profile of available vaccine [2]. Furthermore the comparison between impact data before and after RV introduction will be useful to monitor safety and effectiveness of vaccine [5].

**Acknowledgments**

The Authors are grateful to Sergio Buffa, Franco Belbruno, Gaspare Canzoneri, Nicolò Casuccio, Maria Lia Contrino, Mario Cuccia, Giuseppe Ferrera, Gaetano Geraci, Francesco Iacono, Mario Palermo and Giovanni Puglisi for providing data useful to carry out the analysis.

**References**

1. Vitale F, Tramuto F, Amodio E, Restivo V, Costantino C. Results after one year of rotavirus universal mass vaccination in Sicily. Ital J Pediatr. 2015; 41:77.

2. Costantino C, Restivo V, Cuccia M, Furnari R, Amodio E, Vitale F. Analysis of hospitalizations due to intussusception in Sicily in the pre-rotavirus vaccination era (2003-2012). Ital J Pediatr. 2015; 41:52.

3. Dubé E, Gilca V, Sauvageau C, Bradet R, Bettinger JA, Boulianne N, et al. Canadian paediatricians' opinions on rotavirus vaccination. Vaccine. 2011; 29:3177-82.

4. European Centre for Disease Prevention and Control. Impact of rotavirus vaccination – Generic study protocol. Available at http://ecdc.europa.eu/en/publications/Publications/Rotavirus-impact-vaccination-April-2013.pdf. Accessed June 22, 2017.

5. Restivo V, Costantino C, Tramuto F, Vitale F. Hospitalization rates for intussusception in children aged 0-59 months from 2009 to 2014 in Italy. Hum Vaccin Immunother. 2017; 13:445-449.

## A85 Problems of adolescence, a research carried out by the Italian Paediatric Society in collaboration with MIUR

### Jose Gonzalez^1^, Giovanni Vitali Rosati^2^

#### ^1^Pediatric Department USL Toscana Centro, Florence, Italy; ^2^President of Società Italiana di Pediatria Toscana, Florence, Italy

##### **Correspondence:** Giovanni Vitali Rosati (presidenza.siptoscana@gmail.co)

**Background**

The survey aims at the necessity to encourage both paediatricians and teachers to apply an holistic vision during the practice of their role, promoting not only physical but also relational, emotional and social well-being of future adults.

**Materials and methods**

The leading project has been carried out in Tuscany first, and then broadened to the whole nation.

The anonymous questionnaire handed out was a multiple answers’ test of 60 questions (likert 5) in digital format. The national surveyed sample consists of 9784 teenager individuals.

The fields investigated are: vital and socio-demographic data of the sample, diet and sport, perception to attention paid, psyco-emotional distress, bullying and violence, sexuality, addictions, use of internet and social media, traditional family, divorces and same-sex parents.

**Results**

Fifty-one (51.2%) percent of the surveyed sample report a lack of attention from teachers to their problems outside of the educational setting. Fifty (50%) percent state of having experimented a strong psycho-emotional distress and 15.3% declare of self-inflicting physical injuries because of it. Fifty (50.9%) percent have felt the need of a psychological support but only 15.8% turned to a scholastic support service. Twenty-seven (27.9%) percent of teenagers see themselves overweight, more than 22% have not breakfast at home and 36.4% buy food at school, 52.5% practice some sport. On average, they receive their first smartphone at 11 years old. 24.9% of them prefer online social interactions to live ones. Seventy (79.2 %) percent state that the parents’ divorce is a negative influence on a teenager’s development. 72.4% think that divorce is preferable to a conflicting cohabitation. Sixty (60.1%) percent of divorced parents’ sons agree on feeling not listened to during the divorce. Sixty-six (66.7%) percent are favourable to families composed by same-sex parents. Thirty-three (33.3%) percent declare of being bullied at school, and 12.4% have been victim of cyber-bullying on social media at least once. Thirteen (13.9%) percent of the kids assert that there are aggressive behaviours within their families. Forty-three (43.7%) percent witness to arguments between parents regularly. Sixty-two (62.2%) percent of the surveyed sample declare of not having received any sexual education within the family. 34.1% do not use contraceptive methods during intercourse. Fourteen (14.6%) percent admit of having received sexual offers from adults through dating apps and 3.2% declare of having had intercourse for an economic profit.

**Conclusions**

This study highlights some themes which deserve to be examined in greater depth: the need of a larger attention and openness to dialogue from adults. Adolescents not listened to find their points of reference in friends and social media. An altered perception of their own body does exist in a high percentage. Some final considerations: paediatricians and teachers should be enabled to have a specific and up-to-date training on new family dynamics.

## A86 Transition from pediatric to adult care for youth diagnosed with type 1 diabetes (T1DM): when and how

### Stefano Zucchini (stefano.zucchini@aosp.bo.it)

#### Pediatric Diabetology Centre, Department of Pediatrics, S.Orsola-Malpighi Hospital, Bologna, 40138, Italy

The transition of adolescents and young adults with T1DM from the pediatric centre to the adult centre has been carefully described in the consensus statement of the 3 Italian Scientific Society dealing with subjects with T1DM, i.e. ISPED (Italian Society for Pediatric Endocrinology), ISD (Italian Society of Diabetology) and AMD (Association of Medical Diabetology) [1]. Both the Consensus and the National Programme on the Diabetic Disease agree on the fact that transition is a critical period in the life of the adolescents with T1DM with significant psychosocial issues and risks of poor outcome. The possibility of a poor adherence with the subsequent increased risk of bad metabolic control and future complications has been well documented [2]: previous studies have shown that physician continuity and intensive care coordination can help improve patient transition to adult care [3]. In the US leaving pediatric care is associated with a 2.5-fold increase in the odds of being in poor glycemic control at the follow-up visit compared with those who stay in pediatric care [4]. Young adults should be followed separately from the older patients with T2DM who may present clinical complications discouraging the young patients to follow-up. A questionnaire sent to all members of the SIEDP showed that the transition process is not homogeneous in the country. Although there is general consensus on the transition age, i.e. 18 years, the pediatric diabetologist may modulate the timing depending on the subject and on local practice of adult care. In view of the known difficulties, both pediatricians and diabetologists should agree on general principles of the process that must be properly announced, gradually carried out and shared by patients and care givers. The Italian Consensus has clearly defined how during the first 6 months both pediatrician and diabetologist should attend the clinics and monitor outcome. All phases of the process should take place in a collaborative climate with sufficient time to discuss all the aspects of the past and present disease. Telemedicine should be considered when the adult care is not in the same hospital.

The challenge for the future will be the monitoring of all transition processes throughout the country in order to improve it and create shared strategies between pediatricians and diabetologists.

References

1. Documento di consenso Gruppo di Studio SIEDP- AMD-SID. Transizione dei giovani con diabete mellito verso l’età adulta. Passaggio dal pediatra al medico dell’adulto. Una proposta operativa nazionale. Il Giornale di AMD. 2010; 13:159-168.

2. Bryden KS, Dunger DB, Mayou RA, Peveler RC, Neil HA. Poor prognosis of young adults with type 1 diabetes: a longitudinal study. Diabetes Care. 2003; 26:1052–1057.

3. Cadario F, Prodam F, Bellone S, Trada M, Binotti M, Trada M, et al. Transition process of patients with type 1 diabetes (T1DM) from paediatric to the adult health care service: a hospital-based approach. Clin Endocrinol. 2009; 71:346–350.

4. Garvey KC, Telo GH, Needleman JS, Forbes P, Finkelstein JA, Laffel LM. Health care transition in young adults with type 1 diabetes: perspectives of adult endocrinologists in the U.S. Diabetes Care. 2016; 39:190-7.

## A87 Meningococcal B vaccine

### Gian Vincenzo Zuccotti, Chiara Mameli

#### Department of Pediatrics, V. Buzzi Hospital, University of Milan, Milan, Italy

##### **Correspondence:** Gian Vincenzo Zuccotti (gianvincenzo.zuccotti@unimi.it)

In recent years Neisseria meningitidis serogroup (MenB) has been highlighted as the main cause of meningococcal invasive diseases not only in most temperate countries but in many parts of the world [1]. Many efforts were done to develop a safe and effective vaccine against this serogroup in the last century. Capsular polysaccharide and outer membrane vesicles (OMVs) vaccines were tested in clinical trials and during outbreaks however they were not introduced into routine immunization practice because of safety issues, protection largely restricted to the vaccine strain and limited efficacy in young children [2]. Due to the failure of a conventional approach to develop a universal vaccine against serogroup B, a new approach, termed reverse vaccinology and based on genomics, has been employed since 2000. This approach was used to identify novel antigens for the development of a new multicomponent vaccine (4CMenB) [3]. 4CMenB is composed by four antigenic components, two of which are presented as fusion proteins: Neisseria adhesin A (NadA), factor H-binding protein (fHbp) fused with GNA2091, Neisseria heparin-binding antigen (NHBA) fused with GNA1030 and OMVs from the New Zealand strain NZ98/254. Since 2013 4CMenB has been licensed in Europe, Australia, Canada and USA with different immunization schedules.

Clinical trials involving adults, adolescents, children and infants showed 4CMenB has a good immunogenicity and safety profile as well as a good acceptability among parents and heath care workers [4,5]. Coverage estimates are similar to or better than other recently approved vaccines, ranging from 66% in Canada to 91% in Unites States. 4CMenB was also introduced during meningococcal outbreaks in USA [6,7]. Some points still remain to be clarified such as the best immunization strategy, the effect of 4CMenB on carriage, the long term persistence of protective bactericidal antibodies titers, long term safety outcomes, the possible emergence of N. meningitidis escape mutants and the vaccine cost effectiveness.

**References**

1. Jafri RZ, Ali A, Messonnier NE, Tevi-Benissan C, Durrheim D, Eskola J, et al. Global epidemiology of invasive meningococcal disease. Popul Health Metr. 2013; 11:17.

2. Sadarangani M, Pollard AJ. Serogroup B meningococcal vaccines-an unfinished story. Lancet Infect Dis. 2010; 10:112-124.

3. Mameli C, Galli E, Mantegazza C, Fabiano V, Zuccotti GV. The multicomponent meningococcal serogroup B vaccine (4CMenB): origin, composition, health impact and unknown aspects. Future Microbiol. 2015; 10:1579-98.

4. Gossger N, Snape MD, Yu LM, Finn A, Bona G, Esposito S, et al. Immunogenicity and tolerability of recombinant serogroup B meningococcal vaccine administered with or without routine infant vaccinations according to different immunization schedules: a randomized controlled trial. JAMA. 2012; 307:573-582.

5. Mameli C, Faccini M, Mazzali C, Colella G, Duca PG, Zuccotti GV. Acceptability of meningococcal serogroup B vaccine among parents and health care workers in Italy: a survey. Hum Vaccin Immunother. 2014; 10:3004-3010.

6. Vogel U, Taha MK, Vazquez JA, Findlow J, Claus H, Stefanelli P et al. Predicted strain coverage of a meningococcal multicomponent vaccine (4CMenB) in Europe: a qualitative and quantitative assessment. Lancet Infect Dis. 2013; 13:416-425.

7. Medini D, Stella M, Wassil J. MATS: Global coverage estimates for 4CMenB, a novel multicomponent meningococcal B vaccine. Vaccine. 2015; 33:2629-2636.

